# Type S Non‐Ribosomal Peptide Synthetases for the Rapid Generation of Tailormade Peptide Libraries**

**DOI:** 10.1002/chem.202103963

**Published:** 2022-03-29

**Authors:** Nadya Abbood, Tien Duy Vo, Jonas Watzel, Kenan A. J. Bozhueyuek, Helge B. Bode

**Affiliations:** ^1^ Max-Planck-Institute for Terrestrial Microbiology Department of Natural Products in Organismic Interactions 35043 Marburg Germany; ^2^ Molecular Biotechnology Institute of Molecular Biosciences Goethe University Frankfurt 60438 Frankfurt am Main Germany; ^3^ Senckenberg Gesellschaft für Naturforschung 60325 Frankfurt am Main Germany

**Keywords:** combinatorial biosynthesis, natural products, NRPS engineering, peptides, SYNZIPs

## Abstract

Bacterial natural products in general, and non‐ribosomally synthesized peptides in particular, are structurally diverse and provide us with a broad range of pharmaceutically relevant bioactivities. Yet, traditional natural product research suffers from rediscovering the same scaffolds and has been stigmatized as inefficient, time‐, labour‐ and cost‐intensive. Combinatorial chemistry, on the other hand, can produce new molecules in greater numbers, cheaper and in less time than traditional natural product discovery, but also fails to meet current medical needs due to the limited biologically relevant chemical space that can be addressed. Consequently, methods for the high throughput generation of new natural products would offer a new approach to identifying novel bioactive chemical entities for the hit to lead phase of drug discovery programs. As a follow‐up to our previously published proof‐of‐principle study on generating bipartite type S non‐ribosomal peptide synthetases (NRPSs), we now envisaged the de novo generation of non‐ribosomal peptides (NRPs) on an unreached scale. Using synthetic zippers, we split NRPSs in up to three subunits and rapidly generated different bi‐ and tripartite NRPS libraries to produce 49 peptides, peptide derivatives, and *de novo* peptides at good titres up to 145 mg L^−1^. A further advantage of type S NRPSs not only is the possibility to easily expand the created libraries by re‐using previously created type S NRPS, but that functions of individual domains as well as domain‐domain interactions can be studied and assigned rapidly.

## Graphical abstract

**Following a** previously published proof‐of‐principle study on generating bipartite type S non‐ribosomal peptide synthetases (NRPSs), the de novo generation of non‐ribosomal peptide libraries with unprecedented simplicity was envisaged. By using synthetic zippers, NRPSs were split in up to three subunits to produce 49 peptides at titres up to 145 mg L^−1^.

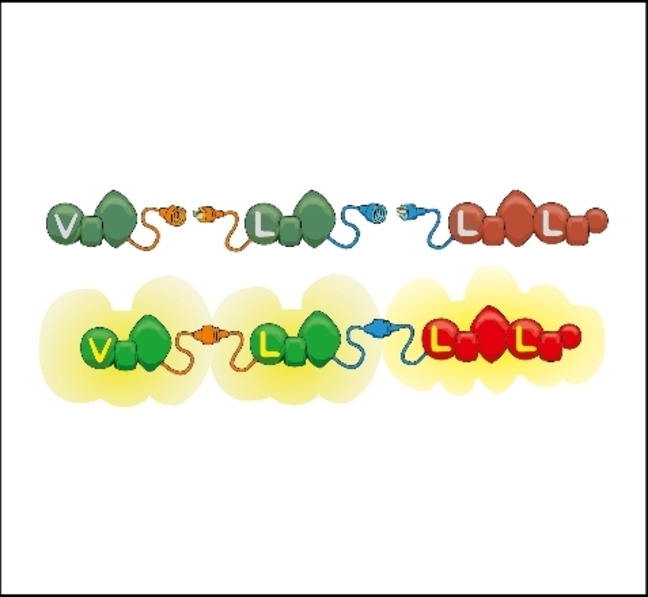

## Introduction

Natural products (NPs) have been used throughout the ages for the treatment of a wide range of medical conditions^[1]^ and still continue to be of particular importance in drug development today.^[2]^ Especially bacterial NPs derived from modular megasynth(et)ases, such as non‐ribosomal peptides (NRPs) and polyketides (PKs), made a major contribution to modern pharmacotherapy, *inter alia*, for tackling infectious diseases and cancer.^[3]^ Nevertheless, although NPs are structurally diverse and bioactive with advantageous properties beyond Lipinski's rule of five’,^[4]^ like higher molecular mass and a greater molecular rigidity which can be valuable in tackling protein‐protein interactions,^[5]^ they also pose challenges for drug discovery. These challenges are mainly due to technical barriers in the identification, characterisation, isolation, screening and optimisation of natural products, which remain time, labour and cost intensive.[^2a^, ^6^] As a result, and due to the lack of adequate solutions, the pharmaceutical industry withdrew from traditional natural product research. With the rapid emergence of antimicrobial resistance (AMR)[^2^, ^7^] and recent technological advances addressing the challenges, such as advances in cultivation,^[8]^ DNA sequencing,[^9^, ^10^] bioinformatics,^[11]^ and synthetic biology^[12]^ interest in natural product research has been reignited.[^6^, ^13^]

Despite the progress made, the complexity of NP structures make it difficult to generate synthetic NP derivatives, for example to explore structure‐activity relationships and develop hits to leads. Thus, many clinical derivatives have been created by means of semi‐synthesis,^[2c]^ i. e. azithromycin^[14]^ and cephalosporin.^[15]^ Due to technical and chemical limitations, such modifications are often limited to a few synthetically accessible functional groups, leaving the actual peptide backbone or amino acid sequence untouched. A commonly stressed solution to this problem is bioengineering, as it provides access to a wider range of structural diversity beyond the limitations of synthetic chemistry.^[16]^ But as rational reprogramming efforts have been met with limited success, progress in the synthetic biology of NPs is of great importance. Therefore, several labs develop tools to enable the reproducible, rapid, and simple genetic manipulation of biosynthetic gene clusters (BGCs) for the biosynthesis of NRP derivatives and even new peptides.[^13^, ^17^]

At the heart of our research are BGCs, encoding multi‐functional enzymatic protein machines that enable the biosynthesis of peptides independently of the ribosome. These machines, denoted as non‐ribosomal peptide synthetases (NRPSs) are in fact assembly lines, and are, *inter alia*, responsible for the synthesis of many antibiotic drug scaffolds in current clinical use, such as penicillin G, vancomycin, and daptomycin.^[18]^ In a NRPS assembly line, multiple repeating modules are responsible for selection, activation, programmed functional group modifications, and coupling of an amino acid to the growing peptide chain. An archetypal minimal module consists of three core domains: an adenylation (A) domain, which selects and activates a substrate, a thiolation (T) domain, on which the activated amino acid as well as all peptide intermediates are covalently attached to, and a condensation (C) domain, which catalyses peptide bond formation between the bound amino‐acyl‐ and peptidyl‐thioester intermediate of the downstream T‐domain. Additionally, several optional *in cis* (as part of the actual NRPS enzyme) or *in trans* (as part of separate enzymes) acting modification domains can be present, introducing structurally complex motives into the peptide chain, for instance epimerisation, methylation, hydroxylation, and glycosylation patterns.^[18]^

Most recently, we developed a novel synthetic type of NRPSs (type S)^[17c]^ with reduced structural complexity compared to wildtype (WT) type A NRPSs. Type S NRPSs are characterised by ‘small’ individually expressible chimeric NRPS protein subunits with attached synthetic leucine zippers, referred to as SYNZIPs (SZs).^[19]^ Type S subunits can be co‐expressed and are quickly interchangeable to generate new assembly lines and peptide derivatives, respectively, quickly and with only a minimum of lab work involved. We were able to showcase how type S NRPSs can be created via splitting one protein NRPSs into two individually expressible proteins (subunits) in between eXchange Unit (XU) building blocks (A−T−C tri‐domain units)^[17c]^ by leveraging the established splicing position within the C−A di‐domain linker region (W][NATE)^[17a]^ to introduce SZs. Due to the high bio‐combinatorial potential of type S NRPSs and the possibility to reuse formerly cloned type S subunits in new combinations, they bear the great chance to accelerate NRPS research and NRP based early drug discovery efforts.

Here, we show the potential of type S NRPSs beyond the limitations of the XU concept. We not only sought to demonstrate (I) the possibility to create a bipartite type S NRP library by using the building blocks of only one single NRPS system, but (II) to introduce SZs within all possible NRPS linker regions. Eventually, (III) to further increase the bio‐combinatorial potential of type S NRPSs, we aimed at dividing the NRPS component of distinct BGCs into three individually expressible subunits to create tripartite NRPS libraries.

## Results

### Bipartite type S NRPS library

**Figure 1:**
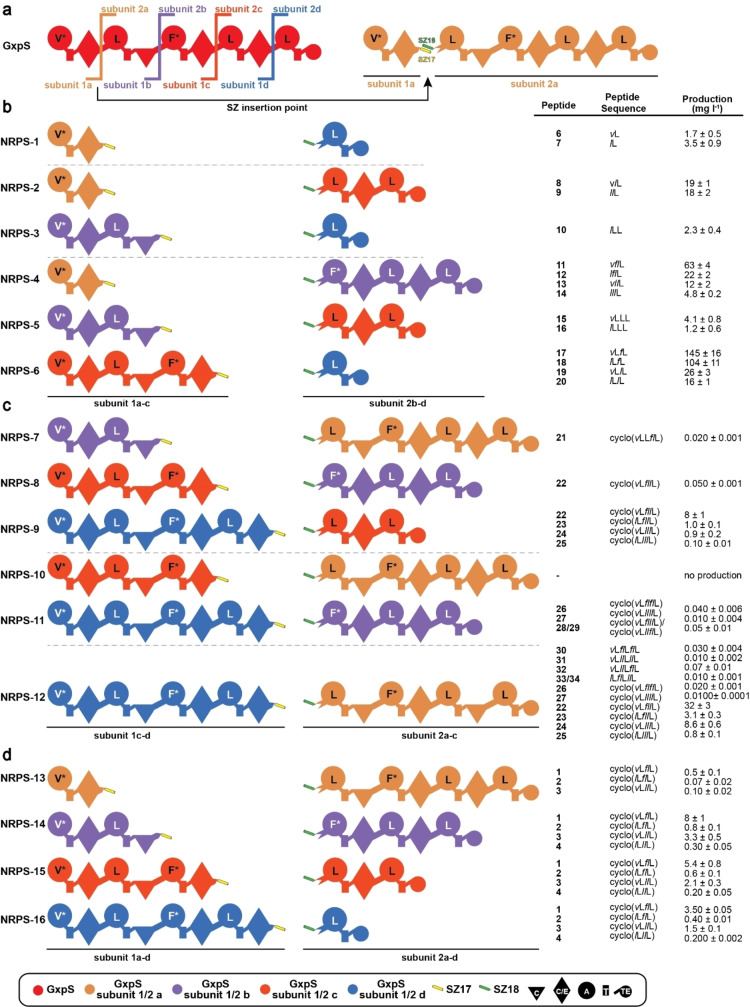
Bipartite GxpS Library. a) SZ 17 : 18 insertion between XUs 1 & 2, 2 & 3, 3 & 4 and 4 & 5 for the generation of four initiating (subunit‐1a, ‐1b, ‐1c, ‐1d) and four terminating building blocks (subunit‐2a, ‐2b, ‐2c, ‐2d). b) Generated truncated type S GxpS systems (NRPS‐1 to ‐6), c) elongated type S GxpS systems (NRPS‐7 to ‐12), and d) wild type length type S GxpS systems (NRPS‐13 to ‐16) are shown. Corresponding peptide yields (mg/L) and standard deviations are obtained from biological triplicate experiments. For domain assignment the following symbols are used: (A, large circles), (T, rectangle), (C, triangle), (C/E, diamond), (TE, small circle); substrate specificities are assigned for all A domains and indicated by capital letters; superscripted asterisks (*) indicate promiscuous A domains that activate leucine as a minor substrate.

To create a bipartite type S NRPS^[17c]^ library from only one single starting NRPS, we chose the GameXPeptide synthetase (GxpS) from *Photorhabdus luminescens* TT01.^[20]^ GxpS, which is responsible for the biosynthesis of four cyclic GameXPeptides (GXP) A−D (**1**–**4**, Supporting Information Figure S1), was split into all possible subunits in between individual XUs. These subunits differed in the number of XUs ranging from 1–4 XUs, both from the *N*‐ and *C*‐terminus. In detail, we split GxpS between XUs 1 & 2, 2 & 3, 3 & 4, and 4 & 5, resulting in four initiating (subunit‐1a, ‐1b, ‐1c, and ‐1d) and four terminating building blocks (subunit‐2, ‐2a, ‐2b, ‐2c, and ‐2d) (Figure 1a). To functionally create type S NRPS systems, each individual initiating subunit was heterologously co‐expressed in *E. coli* DH10B::mtaA^[21]^ together with each of the terminating subunits. With this procedure, 16 unique type S NRPSs were generated from a single WT NRPS.

Notably, HPLC‐MS analysis revealed that all but one type S NRPSs (Figure 1c, NRPS‐10) showed catalytic activity, producing detectable amounts of overall 34 unique linear and cyclic peptides (**1**–**34**, Figure 1) of varying length at titres up to 145 mg L^−1^ (Figure 1). Throughout the present work, the resulting peptides (Table S2) and yields were confirmed by HPLC‐MS/MS and comparison of retention times with synthetic standards (*c.f*. Supporting Information Table S1 and Figures S1–49).

An additional strength of this approach lies in the possibility to study and characterise individual domains (i. e., C & TE) as well as domain‐domain (i. e., C−A) interface interactions with respect to their substrate specificity or compatibility. NRPS domain‐domain interfaces are thought to form flexible and changing domain‐domain contacts during the course of the catalytic cycle, which help the NRPS machinery to run and carry out catalytic reactions in an orchestrated manner.[^18^, ^22^] Traditionally, such characterisations were only done in vitro.^[23]^ However, here, the presented GxpS derived NRPS set (NRPS‐1 to −16) not only enables interesting conclusions concerning the compatibility of differing C−A interface types, but also to quickly deduce the GxpS_TE‐domains’ capacity to cyclise peptides differing in length from the wild type (WT) products.

At a glance, the chimeric set of 16 GxpS derived type S NRPSs consists of six truncated (NRPS‐1 to ‐6, Figure 1b), five elongated (NRPS‐7 to ‐12, Figure 1c), and five WT‐length assembly‐lines (NRPS‐13 to 16, Figure 1d). From the subset of truncated NRPSs 1 to 6 we detected linear di‐ (**6** & **7**), tri‐ (**8**–**10**), and tetra‐ (**11**–**20**) peptides, showing the expected range of derivatives due to the substrate promiscuity of GxpS_A1 (Val, Leu) and A3 (Phe, Leu). While cyclic dipeptides like diketopiperazines are found in nature^[24]^ or as truncated NRPs^[25]^ but mainly are generated by cyclodipeptide synthases using tRNA‐activated amino acids,^[26]^ the generation of tri‐peptides is at least also conceivable ‐ although we are not aware of any cyclic tri‐peptides generated by NRPSs in nature but chemically synthesized examples exist.^[27]^ Cyclic tetra‐peptides are common and numerous NRPS‐based examples exist (e. g. fungisporin,^[28]^ tentoxin,^[29]^ azumamides A−E,^[30]^ xenotetrapeptide^[31]^). However, according to our data, the GxpS‐TE does not seem to be able to cyclise the synthesised tri‐ and tetra‐peptides, suggesting that a length of five consecutive building blocks is its lower limit for cyclisation (NRPS‐13 to ‐16).

Looking at the upper boundary, NRPS‐7 to ‐9 were able to produce and cyclise hexa‐peptides (**21**–**25**) and NRPS‐11 even synthesised cyclic hepta‐peptides (**26**–**29**). However, the TE‐domain seems to have reached its capacity to efficiently cyclise peptides at a length of 7 consecutive amino acid building blocks. Although we also detected cyclic peptides (**22**–**27**) from production cultures of the 8‐modular NRPS‐12, these cyclic peptides are presumably autocatalytically cyclised shunt products of enzyme‐bound hexa‐ and heptapeptides. NRPS‐12 only was capable to hydrolyse the synthesised octapeptides (**30**–**34**), resulting in linear octapeptide derivatives (Figure 1d).

Overall, through this simple and quick experimental procedure, we found that the TE domain of GxpS is quite versatile, accepting a range of peptides from two to at least eight building blocks, but is only able to effectively catalyse cyclisation within a narrow range of five to seven building blocks. In turn, information about TE domains’ substrate specificities and preferences, respectively, gained via generating a series of type S NRPSs will help to guide future engineering projects in identifying suitable termination domains. This is of particular interest when it comes to large scale NRPS engineering campaigns, as TE‐domains that are more flexible with respect to peptide length and amino acid sequence have a broader range of application. The approach shown is not only superior to in vitro characterisation in terms of workload, but also much cheaper (no SNAC peptides need to be synthesised), more robust (less spontaneous or autocatalytic side effects), scalable, can be performed in high throughput, and does not suffer from the in vitro bias known for excised NRPS proteins as recently described.^[32]^

Type S NRPSs can also be used to study C domain specificities and C−A interface compatibilities. Since in the case of GxpS (NRPS‐1 to ‐16), the respective activated and incorporated amino acids are too similar to each other to draw valuable conclusions on C domain specificities ‐ but this was shown previously[^17c^, ^32^] – we focused on characterising the compatibility of different C−A interface types with each other. In general, the C domains are classified into five different groups based on the reactions they catalyse: ^L^C_L_, ^D^C_L_, dualC (C/E), C_start_, and C_term_.^[18]^ However, in our example the type of C−A interfaces depend on whether there is a C or C/E domain directly upstream of the A domain, resulting in C−A or C/E−A type interfaces – as confirmed by phylogentic analysis (Supporting Information Figure S50).

In brief, depending on whether or not the interface type naturally occurring at a specific site has been altered, we observed major differences in the production titres between NRPSs of similar length and amino acid composition, suggesting that C−A interface types indeed play an important role when it comes to (re‐)designing NRPSs. For instance, the four‐modular type S NRPSs (NRPS‐4 to ‐6, Figure 1b) produced tetra‐peptides (**11**‐**20**) with yields varying widely, ranging from 4.1 mg/L to 144.5 mg/L. As could be expected, the best producing system, NRPS‐6 (144.5 mg L^−1^), has the same interface type (C/E−A) and an interface most similar to that of the WT C_5_‐A_5_ interface (Identity 89.3 %, Supporting Information Table S7). For NRPS‐4, in which the interface type changed from C−A to C/E−A, we observed high (63 mg/L) but still significant lower yields than for NRPS‐6. Eventually, expression of NRPS‐5 resulted in the lowest titre (4.1 mg/L), indicating that changes from C/E−A to C−A have a greater impact on production than changes from C−A to C/E−A. These observations further are supported by NRPS‐2 and ‐3 and by NRPS‐7 to ‐9. NRPS‐2 and ‐3 synthesised **8**, **9** and **10**. While NRPS‐2 with the same interface type as the WT produced **8** and **9** at titres of 19 mg/L and 18 mg/L, respectively (Figure 1b), the switch from C/E−A type to C−A type in NRPS‐3 resulted in a sharp drop in production of **10** to 2.3 mg/L. Accordingly, comparing the titres of the hexa‐peptide (**21**–**25**) producing NRPSs (NRPS‐7 to ‐9), the WT‐like C−A interface harbouring NRPS‐9 showed ∼40 to ∼20‐fold higher titres than NRPS‐7 and ‐8, respectively (Figure 1c).

In combination with the previously described extended gatekeeping function,[^32^, ^33^] describing the influence of C domains and the particular formed C−A interface on the catalytic activity and substrate selectivity of A domains, our data helps to refine the NRPS design principles published.[^17a^, ^17b^, ^17c^] We assume that altering the C domain type directly upstream of an A domain of interest substantially impairs C−A di‐domain contacts, resulting in reduced catalytic activity of the A domain and therefore overall productivity of the respective NRPS protein. The observed reduced catalytic activity might be due to an altered non‐beneficial spatial arrangement of the chimeric C−A interface,^[22a]^ disturbing the rotation^[34]^ of the A domain's *C*‐terminal subdomain during a catalytic cycle. In retrospect, this might also explain why some of our previously published recombinant NRPS systems showed reduced production titres ‐ while others showed no impairment or even increased catalytic activity.[^17a^, ^17c^]

### Other SZ insertion sites

**Figure 2:**
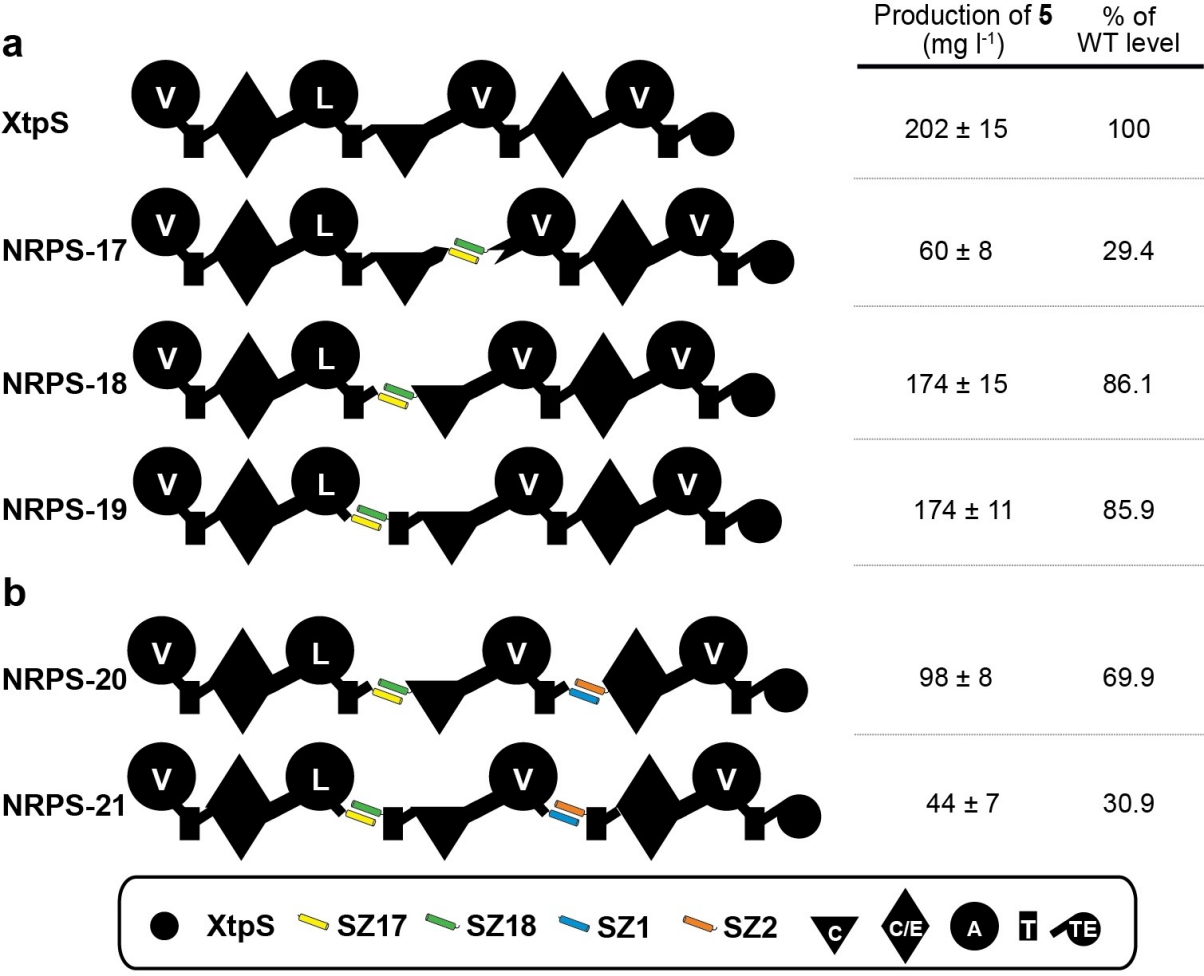
Other splicing positions and Tripartite type S XtpS for the production of cyclo(vLvV) (**5**). a) SZ17 : 18 insertion within the C−A (NRPS‐17), T−C (NRPS‐18) and A−T (NRPS‐19) linker XtpS split into three subunits by the insertion of the SZ1 : 2 pair. b) SZ17 : 18 and SZ1 : 2 pairs were inserted within T−C (NRPS‐20) and A−T (NRPS‐21). Corresponding yields (in mg/L) and standard deviations of **5** are determined from biological triplicate experiments.

To further explore and extend the applicability of SZs for the construction of type S NRPSs beyond the borders of the XU concept,^[17a]^ we decided to introduce SZ17 : 18 within the T−C (NRPS‐18) and A−T (NRPS‐19) linker regions of the cyclic xenotetrapeptide (**5**; cyclo(vLvV)) producing synthetase (XtpS) from *Xenorhabdus nematophila* ATCC 19061^[31]^ (Supporting Information Figure S51) – and compared resulting peptide yields with WT XtpS and NRPS‐17 (Figure 2), which was constructed previously.^[17c]^ Both, NRPS‐18 and ‐19 synthesized **5** at ∼86 % compared to WT XtpS and ∼280 % compared to NRPS‐17.

While the catalytic activity of NRPS‐18 was not surprising, as the introduced SZs are mimicking natural DDs,^[35]^ the observed good activity of NRPS‐19 was unexpected. During a catalytic cycle of a module, especially the A−T interaction is considered as highly dynamic. After the adenylation reaction, the A_sub_‐domain must fulfil a torsion of 140° in respect to the A_core_‐domain such that the *holo*‐T‐domain can meet the distance to the activated amino acid (thiolation reaction).^[18]^ Thus, it was assumed that the additional rigidity, inserted by the structured α‐helical amino acid stretches of the SYNZIPs, would result in loss of function. The recently gathered structural data of large constructs of the linear gramicidin synthesising NRPS (LgrA),^[36]^ might serve as an explanation for the observed activity. There a very high structural flexibility was reported, potentially bringing closely together domains that are far apart in protein sequence and therefore facilitating synthetic cycles with inserted tailoring domains, unusual domain arrangements like A−C−T,^[37]^ module skipping,^[38]^ and presumably also SZs.

More bipartite type S NRPSs (NRPS‐40 to ‐45), split in between (T][C) and within modules (A][T) as well as within C domains (C_Dsub_][C_Asub_) are depicted in Supporting Information Figures S52 and S53.

### Tripartite type S NRPS library

The potential of bipartite type S NRPSs to generate bio‐combinatorial libraries from a small set of NRPS subunits was shown previously^[17c]^ and above (Figures 1 and 2a). But, the bio‐combinatoric potential could further be increased if it were possible to split NRPS systems into three or more subunits (c.f. Supporting Information S54 and S55).

For a first proof of concept, we inserted a second SZ pair (SZ1 : 2) into both NRPS‐18 and NRPS‐19 (Figure 2a) to establish an orthogonal interaction network (Figure 2b). The resulting tripartite type S NRPSs‐20 and ‐21 are split in between modules 2–3 & 3–4 (NRPS‐20) and within the A−T linker regions of modules 2 & 3 (NRPS‐21), respectively (Figure 2b). Both, NRPS‐20 and ‐21, produced **5** with 69.9 % and 30.9 % compared to WT XtpS but also with decreased yields compared to their bipartite counterparts (NRPS‐18 & ‐19). In addition to the cumulative effect of inserted impairments, caused by a higher degree of engineering, we assume that SZ1 : 2 also contributed to the reduced production titre of **5** since the SZ1 : 2 pair is significantly longer than SZ17 : 18 (Table S8) and probably disturbs catalytic efficiency of the tripartite type S XtpS variants by the inserted additional rigidity. Although NRPS‐20 produced **5** at slightly higher titres than NRPS‐21, in a next step we decided to use the A−T splicing position (NRPS‐19 & ‐21) for the construction of a small but diverse tripartite NRPS library with subunits derived from various *Photorhabdus* and *Xenorhabdus* strains, because T−C−A tri‐domains as catalytically active units to reprogram NRPSs are underrepresented in the available literature.

**Figure 3:**
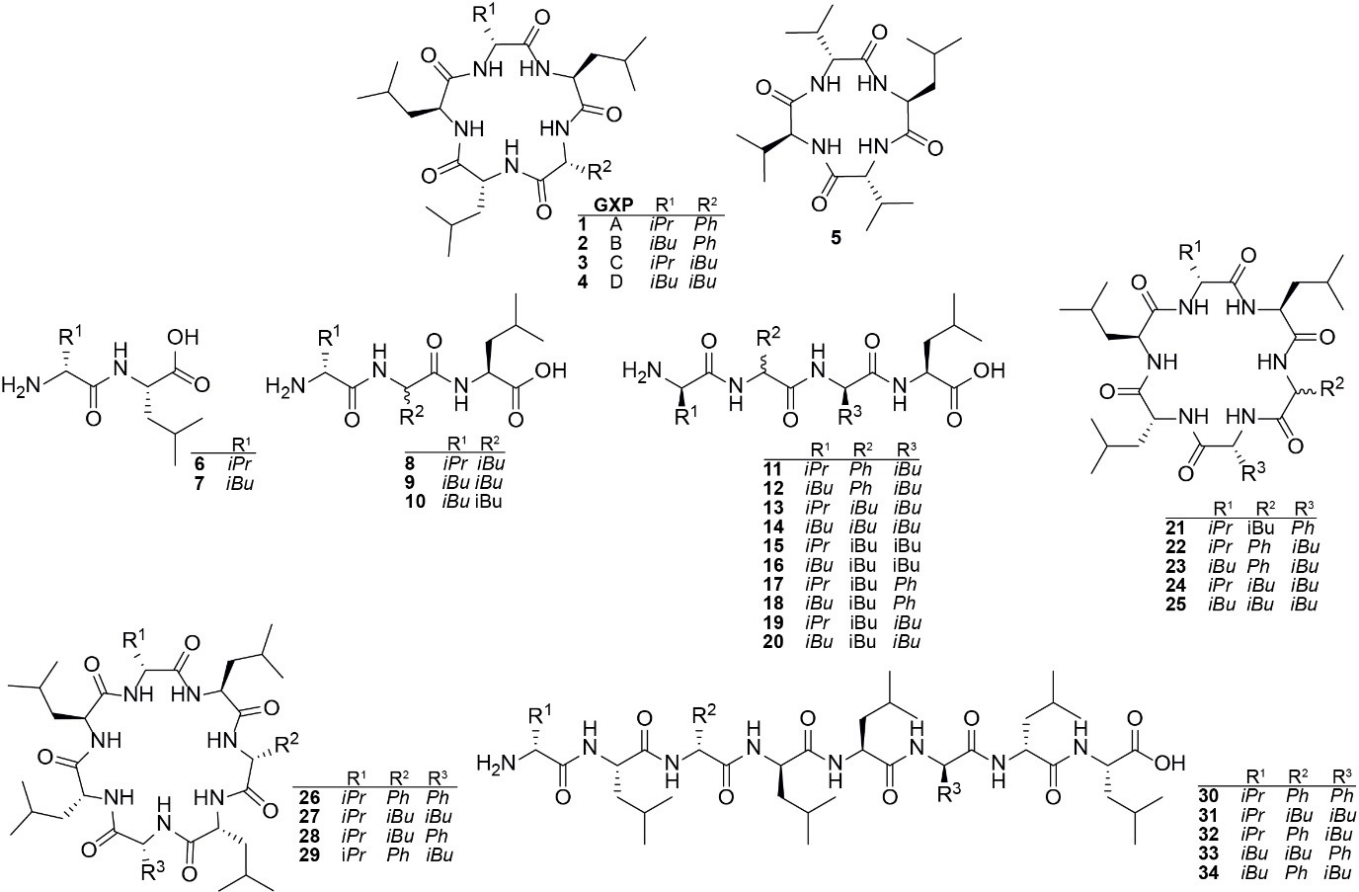
Structures of produced compounds. GameXPeptide A−D (**1**–**4**), xenotetrapeptide (**5**) and GXP derivatives (**6**–**34**) are depicted.

Eleven NRPS subunits with attached SZs were extracted from five different BGCs, namely from GxpS, XtpS as well as from the gargantuanin (GarS), xenolindicin (XldS),^[21]^ and the szentiamide (SzeS)^[39]^ producing synthetases. Overall 18 (NRPS‐22 to ‐38) from 45 possible co‐expressions of three plasmids each yielded detectable amounts (0.1–38 mg L^−1^) of 18 different peptides, 13 of which were new (Figure 3, Supporting Information Figures S32–S49). Despite the method's general simplicity, the overall efficacy or recombination potential of T−C−A units compared to XUs appears to be more restricted.^[17c]^ For example, neither co‐expression of all type S subunits to reconstitute SzeS, nor any combination involving the Ser and Thr specifying subunits from XldS and GarS, yielded any detectable peptide, respectively. These results probably indicate an incompatibility of formed chimeric A−T interfaces or substrate incompatibilities at the respective C domains donor site. Yet, in light of previous results concerning C domain specificities,[^23^, ^32^, ^40^] the latter seems to be unlikely. Especially as we were not even able to reconstitute catalytic activity of the tripartite SzeS, we concluded that the respective subunits have lost their functionality. Due to the sequence and structural flexibility of the targeted A−T linker regions, key interactions within protein‐protein interfaces that must be maintained are hard to predict. Therefore, it is likely that the insertion of SZ pairs structurally affected these subunits, resulting in a loss of function or their ability to ‘communicate’ with downstream subunits – as was already expected for NRPS‐21 (Figure 2b).

Furthermore, for some tripartite NRPSs (NRPS‐33, ‐35, ‐36, ‐37 and ‐38), we were able to detect peptides (**41**–**44**, **46** and **47**) only in very low amounts, which might be explained by the aforementioned impairment within the A−T domain interface and/or the mere length of the chosen SZ1 : 2 pair. Taking these points into consideration, we assume that productivity can significantly be increased when another fusion site or another SZ pair is chosen, or, if possible, SZ1 : 2 is truncated. Nevertheless, the amount of peptides produced at this early stage after introducing SZs to enable straightforward NRPS‐based biocombinatorics should not distract from the overall strength and the future potential of this method, in particular if it is possible to optimize it further: *The ability to generate an enormous variety of* new recombinant *NRPSs in an unprecedented short time and with a minimum of lab work involved*.

## Conclusion

Although Nature still bears an enormous variety of natural products only waiting to be discovered,[^2a^, ^41^] traditional methods for the identification and characterisation of new scaffolds from Nature are far from providing enough new chemical entities to meet the increasing demand for innovative bioactive scaffolds, i. e. to treat infectious diseases and cancer. For this reason, the entire natural product community is interested in finding new and efficient ways to unlock the hidden treasures that Nature has in store for us.[^17d^, ^17g^, ^42^] Recent examples are the CRISPR‐Cas9‐based NRPS engineering^[13]^ or DNA‐templated NRPSs.^[17e]^ For easy and fast repurposing of biosynthetic modular assembly line pathways, we recently introduced the concept of type S NRPSs.^[17c]^ In brief, SZs^[19b]^ were leveraged to split single protein NRPSs of Gram‐negative and ‐positive origin within C−A di‐domain linker regions^[17c]^ to biosynthesise linear‐, cyclic‐, lipo‐, formyl‐, and thiazoline containing peptides in a bio‐combinatorial manner.

**Figure 4:**
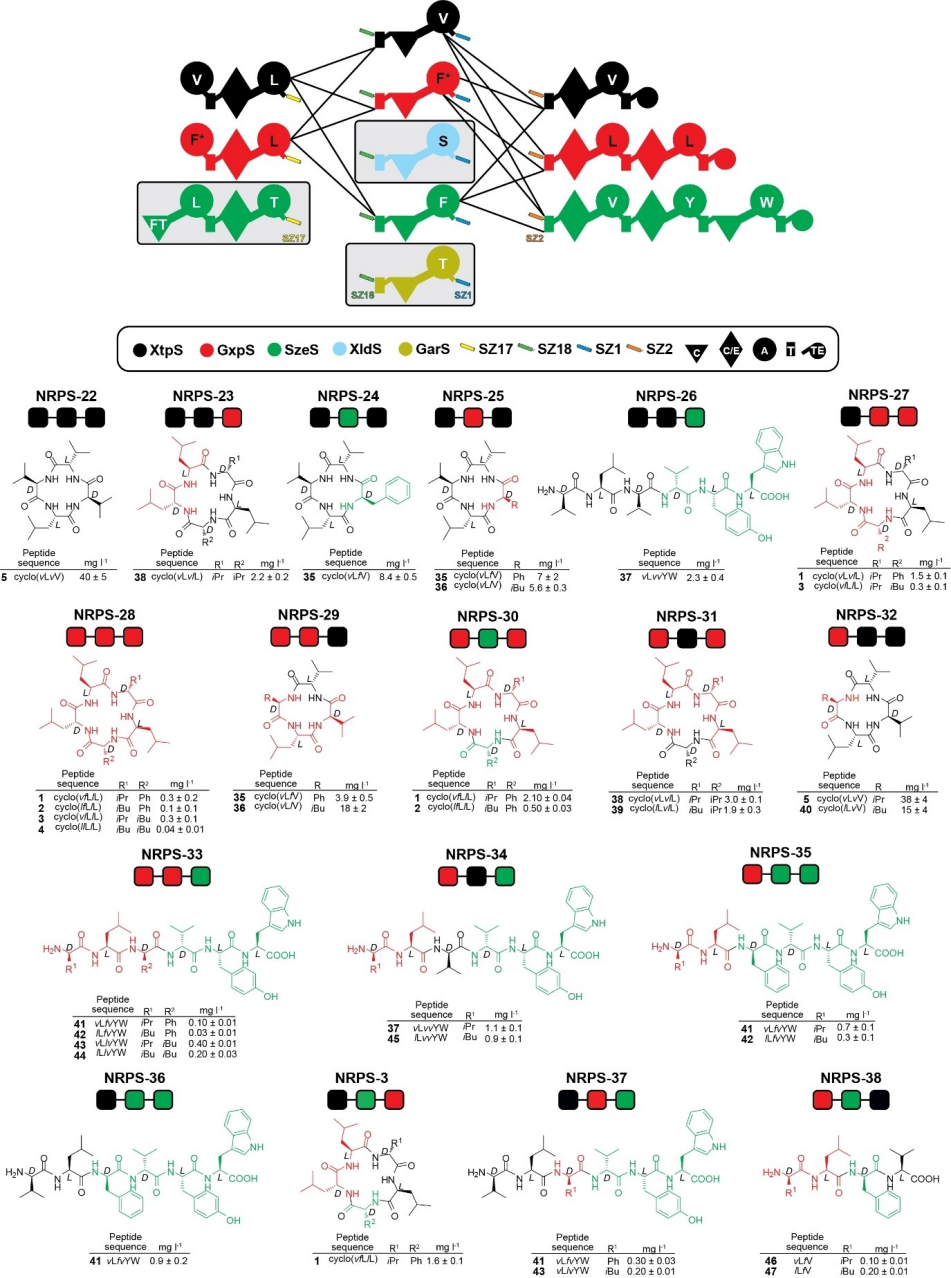
Tripartite type S NRPS library. In the upper section, generated building blocks, depicted in the symbol assignment as introduced previously, are illustrated. Solid lines represent functional combinations. In the lower section, building blocks were simplified and illustrated as boxes representing subunit 1 to 3. From 11 generated and 8 functional building blocks, a total of 18 type S NRPS were confirmed as functional by HPLC‐MS. Corresponding peptide yields (mg/L) and standard deviations were obtained from biological triplicate experiments.

In our present follow up work, we expanded this concept and successfully demonstrated that SZs can even be used to functionally turn single‐protein NRPSs into two and three individually expressible NRPS subunits (Figures 2b and 4), respectively ‐ not only by splitting one protein NRPS between C−A di‐domains (Figure 1) but also between T−C (Figure 2) and A−T (Figures 2 and 4) di‐domains. We generated two small NRPS libraries, which in total yielded 34 recombinant type S NRPS and 47 unique peptides in yields ranging from 0.1 to 145 mg L^−1^.

Although the yields currently achieved in the case of tripartite NRPSs (Figure 4) still require further optimisation of the method, the striking advantage of type S NRPSs is that generated libraries can be expanded continuously and at any time, since generated subunits are not covalently linked. Therefore, this method has the potential to generate not only dozens but also hundreds or even thousands of artificial NRPSs in a short time with little effort. To illustrate this better, under ideal conditions and by using tripartite type S NRPSs, a library of 1.000 recombinant NRPSs can be created from only 30 individually expressible type S NRPS building blocks: 10 catalytically active building blocks for each subunit (10 subunit A)×(10 subunit B)×(10 subunit C)=1.000 type S NRPSs).

Consequently, being able to split known NRPSs into individually expressible subunits and to recombine them simply by attaching SZs and co‐expressing a variety of unrelated NRPS subunits in a high‐throughput manner puts us in a position to easily enlarge the known structural diversity, and to outcompete traditional natural product discovery approaches, which suffer from the frequent (re‐) discovery of already known natural products.^[43]^ For the discovery of novel antimicrobials, the advantage of the high‐throughput generation of antimicrobial peptides might be its direct coupling to bioactivity testing, i. e., via nanoFleming,^[44]^ a miniaturized and parallelized high‐throughput inhibition assay.

In summary, a great variety of type S NRPSs can already be achieved from a small set of NRPSs, as exemplified by turning GxpS into 16 artificial type S NRPSs (Figure 1). Since a typical *Photorhabdus* and *Xenorhabdus sp*. genome may comprise more than 20 NRPSs with an average size of 7–9 modules, a large number of type S NRPSs can already be produced from highly related, experimentally validated, and compatible NRPS building blocks (as it would be also the case for any other well‐known natural product producer).

1

## Supporting information

As a service to our authors and readers, this journal provides supporting information supplied by the authors. Such materials are peer reviewed and may be re‐organized for online delivery, but are not copy‐edited or typeset. Technical support issues arising from supporting information (other than missing files) should be addressed to the authors.

Supporting Information

## Data Availability

The data that support the findings of this study are available in the supplementary material of this article.
