# Supplementary material for: Type S Non‐Ribosomal Peptide Synthetases for the Rapid Generation of Tailormade Peptide Libraries
Source: Chemistry. 2022 Mar 29;28(26):e202103963. doi: 10.1002/chem.202103963 (PMC9315016; doi:10.1002/chem.202103963)
Supplement: Supplementary file 1 — Supporting Information [file CHEM-28-0-s001.pdf]

Chemistry–A European Journal

Supporting Information

Type S Non-Ribosomal Peptide Synthetases for the Rapid Generation of Tailormade Peptide Libraries

Nadya Abbood, Tien Duy Vo, Jonas Watzel, Kenan A. J. Bozhueyuek,* and Helge B. Bode*

Table of Contents

1. Material and Methods 4
1.1 Cultivation of Strains 4
1.2 Cloning of biosynthetic gene clusters 4
1.2.1 Bi-partite plasmid library 4
1.2.2 Other splicing positions 5
1.2.2 Tri-partite NRPS library 5
1.3 Heterologous expression of NRPS templates and HPLC-MS analysis 6
1.4 Peptide quantification 6
1.5 Chemical synthesis 6
2. Supplementary tables 7
Table S1. ESI-MS data of all produced peptides. 7
Table S2. HR-MS data of all synthetic standards. 8
Table S3. Theoretical mass-to-charge ratio of non-produced peptides. 9
Table S4. Strains used in this work. 9
Table S5. Plasmids used in this work. 9
Table S6. Oligonucleotides used in this work. 11
Table S7. C-A interface identity. 15
Table S8. Peptide sequences of applied SZ pairs SZ17:18 and SZ1:2. 16
3. Supplementary Figures 16
Figure S1. Schematic representation of the GameXPeptide (1-4) producing type A NRPS (GxpS). 17
Figure S2. Fragmentation pattern of synthetic 1. 18
Figure S3. Fragmentation pattern of synthetic 5. 19
Figure S4. Fragmentation pattern of synthetic 6. 19
Figure S5. Fragmentation pattern of synthetic 8. 20
Figure S6. Fragmentation pattern of synthetic 10. 20
Figure S7. Fragmentation pattern of synthetic 11. 21
Figure S8. Fragmentation pattern of synthetic 15. 21
Figure S9. Fragmentation pattern of synthetic 17. 22
Figure S10. Fragmentation pattern of synthetic 22. 22
Figure S11. Fragmentation pattern of synthetic 26. 23
Figure S12. Fragmentation pattern of synthetic 32. 23
Figure S13. Fragmentation pattern of synthetic 37. 24
Figure S14. Fragmentation pattern of synthetic 41. 24
Figure S15. HPLC/MS data (Figure 1b) of compounds 6 and 7 produced in *E. coli* DH10B::*mtaA*. 25
Figure S16. HPLC/MS data (Figure 1b) of compounds 8 and 9 produced in *E. coli* DH10B::*mtaA*. 25
Figure S17. HPLC/MS data (Figure 1b) of compound 10 produced in *E. coli* DH10B::*mtaA*. 26

Figure S18. HPLC/MS data (Figure 1b) of compounds 11, 12, 13 and 14 produced in *E. coli* DH10B::*mtaA*. 28

Figure S19. HPLC/MS data (Figure 1b) of compounds 15 and 16 produced in *E. coli* DH10B::*mtaA*. 29

Figure S20. HPLC/MS data (Figure 1b) of compounds 17, 18, 19 and 20 produced in *E. coli* DH10B::*mtaA*. 31

Figure S21. HPLC/MS data (Figure 1c) of compound 21 produced in *E. coli* DH10B::*mtaA*. 31

Figure S22. HPLC/MS data (Figure 1c) of compound 22 produced in *E. coli* DH10B::*mtaA*. 32

Figure S23. HPLC/MS data (Figure 1c) of compounds 22, 23, 24 and 25 produced in *E. coli* DH10B::*mtaA*. 33

Figure S24. HPLC/MS data (Figure 1c) of compounds 26, 27 and 28/29 produced in *E. coli* DH10B::*mtaA*. 34

Figure S25. HPLC/MS data (Figure 1c) of compounds 30, 31, 32 and 33/34 produced in *E. coli* DH10B::*mtaA*. 35

Figure S26. HPLC/MS data (Figure 1d) of compounds 1, 2 and 3 produced in *E. coli* DH10B::*mtaA*. 36

Figure S27. HPLC/MS data (Figure 1d) of compounds 1, 2, 3 and 4 produced in *E. coli* DH10B::*mtaA*. 37

Figure S28. HPLC/MS data (Figure 1d) of compounds 1, 2, 3 and 4 produced in *E. coli* DH10B::*mtaA*. 38

Figure S29. HPLC/MS data (Figure 1d) of compounds 1, 2, 3 and 4 produced in *E. coli* DH10B::*mtaA*. 39

Figure S30. HPLC/MS data refers to Figure 2 (NRPS-18, NRPS-19) of compound 5 produced in *E. coli* DH10B::*mtaA*. 40

Figure S31. HPLC/MS data refers to Figure 2 (NRPS-20, NRPS-21) of compound 5 produced in *E. coli* DH10B::*mtaA*. 41

Figure S32. HPLC/MS data refers to Figure 4 (NRPS-22) of compound 5 produced in *E. coli* DH10B::*mtaA*. 41

Figure S33. HPLC/MS data refers to Figure 4 (NRPS-23) of compound 38 produced in E. coli DH10B::mtaA. 42

Figure S34. HPLC/MS data refers to Figure 4 (NRPS-24) of compound 35 produced in *E. coli* DH10B::*mtaA*. 42

Figure S35. HPLC/MS data refers to Figure 4 (NRPS-25) of compounds 35 and 36 produced in *E. coli* DH10B::*mtaA*. 43

Figure S36. HPLC/MS data refers to Figure 4 (NRPS-26) of compound 37 produced in *E. coli* DH10B::*mtaA*. 43

Figure S37. HPLC/MS data refers to Figure 4 (NRPS-27) of compounds 1 and 3 produced in *E. coli* DH10B::*mtaA*. 44

Figure S38. HPLC/MS data refers to Figure 4 (NRPS-28) of compounds 1, 2, 3 and 4 produced in *E. coli* DH10B::*mtaA*. 45

Figure S39. HPLC/MS data refers to Figure 4 (NRPS-29) of compounds 35 and 36 produced in *E. coli* DH10B::*mtaA*. 46

Figure S40. HPLC/MS data refers to Figure 4 (NRPS-30) of compounds 1 and 2 produced in *E. coli* DH10B::*mtaA*. 46

Figure S41. HPLC/MS data refers to Figure 4 (NRPS-31) of compounds 38 and 39 produced in *E. coli* DH10B::*mtaA*. 47

Figure S42. HPLC/MS data refers to Figure 4 (NRPS-32) of compounds 40 and 5 produced in *E. coli* DH10B::*mtaA*. 47

Figure S43. HPLC/MS data refers to Figure 4 (NRPS-33) of compounds 41, 42, 43 and 44 produced in *E. coli* DH10B::*mtaA*. 48

Figure S44. HPLC/MS data refers to Figure 4 (NRPS-34) of compounds 37 and 45 produced in *E. coli* DH10B::*mtaA*. 49

Figure S45. HPLC/MS data refers to Figure 4 (NRPS-35) of compounds 41 and 42 produced in *E. coli* DH10B::*mtaA*. 50

Figure S46. HPLC/MS data refers to Figure 4 (NRPS-36) of compound 41 produced in *E. coli* DH10B::*mtaA*. 50

Figure S47. HPLC/MS data refers to Figure 4 (NRPS-37) of compound 1 produced in *E. coli* DH10B::*mtaA*. 51

Figure S48. HPLC/MS data refers to Figure 4 (NRPS-38) of compounds 43 and 41 produced in *E. coli* DH10B::*mtaA*. 51

Figure S49. HPLC/MS data refers to Figure 4 (NRPS-39) of compounds 46 and 47 produced in *E. coli* DH10B::*mtaA*. 52

Figure S50. Phylogenetic tree of extracted C-A and C/E-A didomains from GxpS. 52

Figure S51. Alignments of XtpS linker sequences. 53

Figure S52. Additional examples of bipartite type S NRPS split in between and within RtpS modules[1]. 53

Figure S53. Additional examples of bipartite type S NRPS split within modules of xefoampeptide-producing NRPS (XfpS)[8] and between XfpS and XtpS. 54

Figure S54. Extended comparison of workflows to generate chimeric NRPSs. 55

Figure S55. Comparing the throughput between di- and tripartite NRPSs. Number of hybrids increases exponentially with the number of building blocks per subunit for both di- and tripartite NRPSs. 56

Figure S56. Remaining possible tripartite typeS NRPSs and their predicted peptide sequences. 57

Figure S57. HPLC/MS data refers to Supplementary Figure 40 (NRPS-40) of compounds 48, 49, 50 and 51 produced in *E. coli* DH10B::*mtaA*. 58

Figure S58. HPLC/MS data refers to Supplementary Figure 41 (NRPS-41) of compound 55 produced in *E. coli* DH10B::*mtaA*. 58

Figure S59. HPLC/MS data refers to Supplementary Figure 41 (NRPS-44) of compounds 52 and 53 produced in *E. coli* DH10B::*mtaA*. 59

Figure S60. HPLC/MS data refers to Supplementary Figure 41 (NRPS-45) of compound 54 produced in *E. coli* DH10B::*mtaA*. 59

4. Reference 60

1. Material and Methods

1.1 Cultivation of Strains

All *E. coli*, *Photorhabdus* and *Xenorhabdus* strains were cultivated in LB (10 g/L Trypton, 5 g/L yeast extract, 10 g/L NaCl, pH 7,5) or TB liquid medium (12 g/L Tryton, 24 g/L yeast extract, 0.4% (v/v) Glycerin, 10% (v/v), 17 mM $KH2PO4$, 72 mM $K2HPO4$, pH 6.5) at 37°C (*E. coli*) or 30°C (*Photorhabdus*, *Xenorhabdus*) for 16-18 h at 160-200 rpm. 1% (w/v) agar was added for growth on solid LB. If necessary, medium was supplemented 1:1000 with kanamycin- (50 µg/mL in sterile ddH2O), chloramphenicol- (34 µg/mL in ethanol) and/or spectinomycin stock solution (50 mg/mL in sterile ddH2O). For short-time storage LB agar plates were stored either at 4°C (*E. coli*) or 18°C (*Photorhabdus*, *Xenorhabdus*). For permanent storage, liquid cultures were supplemented with 20% (v/v) glycerol and frozen at -80°C.

1.2 Cloning of biosynthetic gene clusters

1.2.1 Bi-partite plasmid library

Basic vectors pCK_0402 and pCOLA_ara/tacI containing SZs 17 and 18 (pENTR-SYNZIP17/18 were a gift from Amy Keating, Addgene plasmids #80671/80672; RRID:Addgene_80671/80672) were already construed before. In brief, cloning (of both vectors) was as follow: primers KB_pACYC_FW/ KB_pACYC_RV and KB_pCOLA_FW/ KB_pCOLA_FW were used to insert SZ encoding sequences of SZ17 and 18 into plasmids pCK_0402 and pCOLA_ara/tacI, respectively. Obtained pCK_0402_SZ17 and pCOLA_ara/tacI_SZ18 were linearized using primers KB_pACYC_II_FW/ KB_pACYC_II_RV and KB_pCOLA_II_FW/KB_pCOLA_II_RV. Plasmids pJW75 and pJW76 were already constructed before and remaining plasmids pNA104-pNA109 were generated by amplifying GxpS subunits 1a, 1c, 1d and 2a, 2c, 2d and cloning building blocks into plasmid BBs and pCK_0402_SZ17 and pCOLA_ara/tacI_SZ18, respectively. Therefore, genomic DNA (gDNA) from *Photorhabuds luminescens* TT01 was isolated using the Qiagen Gentra Puregene Yeast/Bact kit and subunits were amplified by applying primers (na194/na208 (pNA104), na209/na56 (pNA105), na194/na210 (pNA106), na211/na56 (pNA107), na194/na212 (pNA108), na213/na56 (pNA109)) obtained from eurofins genomics containing homologous ends to the plasmid backbones. PCR products were purified using Spin DNA Extraction-Kit (Stratec) and DNA fragments were assembled into linearized BBs by either HotFusion or HiFi cloning. Obtained plasmids were verified by plasmid digest using type II restriction endonucleases (specifically cutting the insert sequence). Correctly, verified plasmids were additionally sanger sequenced by eurofins genomics. Plasmids pNA104, pJW75, pNA106 or

pNA108 (containing subunits 1 fragments) were co-expressed with pNA105, pJW76, pNA107 or pNA109 (containing subunit 2 fragments) into *E. coli*::mtaA in different combinations for the construction of GxpS derivatives.

1.2.2 Other splicing positions

pJW61 and pJW62 were already constructed. Splicing positions within the T-C and A-T linker were determined by aligning XtpS T_2-C_3/T_3-C/E_4 and A_2-T_2/A_3-T_3 linker sequences with linker regions excised from Dhbf crystal structure (Protein Database ID: 5U89) to draw conclusions on secondary structures. Splicing positions were depicted within the sequence motives RV/LP (T-C linker) and VY/AAP (A-T linker) and are illustrated in Fig. S19. For the construction of plasmids pNA2, pNA3 and pNA4, pNA5, XtpS TCA and ATC building blocks were obtained from *Xenorhabdus nematophila* gDNA and building block were cloned into pCOLA_ara/tacI and pCK_0402. Verification of correctly assembled plasmids was done as described before. pNA2, pNA3 and pNA4, pNA5 were co-expressed into *E. coli*::mtaA, respectively.
For the construction of tripartite XtpS systems a second SZ pair was introduced. Therefore, SZs 1 (pQLinkHD-SYNZIP1 was a gift from Amy Keating, Addgene plasmid #80647; RRID:Addgene_80647) and 2 (pQLinkHD-SYNZIP2 was a gift from Amy Keating, Addgene plasmid #80658; RRID:Addgene_80658) were inserted into the T_3-C/E_4 and A_3-T_3 linker and plasmids pNA15/pNA16 (A-T split) and pNA17/pNA18 (T-C split) were constructed. Primers na28/na30 (A-T and T-C split) and na34/na32 (A-T and T-C split) were used to insert the first half of SZ1 and 2 into pCOLA_ara/tacI_SZ18 and pCDF_ara/tacI, respectively. In a second polymerase chain reaction (PCR), the second half of both SZs was introduced by applying primers na29/na30 (A-T and T-C split) and na34/na33 (A-T and T-C split) to obtain pCOLA_ara/tacI_SZ18_SZ1 and pCDF_ara/tacI_SZ2. Plasmids pNA4, pNA15, pNA16 (A-T split) and pNA2, pNA17, pNA18 (T-C split) were transformed into *E. coli*::*mtaA* respectively to reconstitute XtpS.

1.2.3 Tri-partite NRPS library

ATC building blocks for the construction of plasmids pNA24, pNA26, pNA27, pNA28, pNA29, pNA30, pNA31 and pNA35 were obtained from *Photorhabdus luminescens*, *Xenorhabdus szentirmaii* and *Xenorhabdus bovenii* gDNA. Subunits were cloned into pCK_0402_SZ17 (pNA26, pNA29), pCOLA_ara/tacI_SZ18_SZ1 (pNA24, pNA27, pNA30 and pNA35) and pCDF_ara/tacI_SZ2 (pNA28, pNA31) using HotFusion cloning. Verification of correctly assembled plasmids was done as described before. Plasmids pNA4, pNA26 or pNA29 (containing subunits 1 fragments) were co-expressed with pNA15, pNA24, pNA27, pNA30 or pNA35 (containing subunit 2 fragments) and pNA16, pNA28 or pNA31 (containing subunit 3 fragments) into *E. coli*::mtaA in different combinations for the construction of artificial NRPS systems.

1.3 Heterologous expression of NRPS templates and HPLC-MS analysis

After plasmid transformation into *E. coli DH10B*::mtaA, cells were grown overnight in LB medium containing all necessary antibiotics (50 µg/ml kanamycin, 34 µg/ml chloramphenicol, 50 µg/ml spectinomycin). 100 µl of an overnight culture were used to inoculate 10 ml LB medium containing antibiotics, 0.002 mg/mL L-arabinose and 2 % (v/v) XAD-16. After incubation for 72 h at 22 °C, XAD-16 beads were harvested and one culture volume methanol was added. Methanol extraction was conducted for 60 min at 22 °C. The organic phase was filtrated, cleared extract were centrifuged for 20 min at 17000g. and methanol extracts were used for LC-MS analysis. Liquid chromatography was performed on an UltiMate 3000 LC system (Dionex) with an installed C18 column (ACQUITY UPLCTM BEH, 130 Å, 2.1 mm x 100 mm, Waters). Separation was conducted at a flow rate of 0.4 mL/min using acetonitrile (ANC) and water containing 0.1% formic acid (v/v) in a 5-95% gradient over 16 min. Mass spectrometric analyses were performed using an ESI ion-trap mass spectrometer (AmaZon X, Bruker). ESI-MS spectra were recorded in the positive-ion-mode with the mass range from 100-1200 *m/z* and ultraviolet (UV) at 200-600 nm. Evaluation was performed using DataAnalysis 4.3 software (Bruker).

1.4 Peptide quantification

The absolute production titers of were calculated as previously described.[1] Synthetic standards **6** (for quantification of **6** and **7**), **8** (for quantification of **8** and **9**), **10** (for quantification of **10**), **11** (for quantification of **11**-**14**), **15** (for quantification of **15** and **16**), **17** (for quantification of **17**-**20**), **22** (for quantification of **21**-**25**), **26** (for quantification of **26**-**29**), **32** (for quantification of **30**-**34**), **1** (for quantification of **1**-**4** and **38**-**39**) and **5** (for quantification of **5, 35, 36** and **40**) were synthesized in house. **37** (for quantification of **37** and **45**) and **41** (for quantification of **41**-**44** and **40**) were obtained by Synpeptide.

1.5 Chemical synthesis

Chemical synthesis of all peptides was performed as described previously[2]: Linear peptides were synthesized on preloaded resins (H-AA-2ClTrt PS resin, Sigma Aldrich, Germany) on a 25 µM scale with a Syro Wave peptide synthesizer (Biotage, Sweden) by using standard Fmoc/t-Bu chemistry. Fmoc-amino acids were purchased from Carbolution Chemicals (Germany), Iris Biotech (Germany) or Bachem (Switz). Therefore, the resin was placed in a plastic reactor vessel with a Teflon frit and an amount of 6 eq. of amino acid derivative (c = 0.2 M) was activated in situ at room temperature with 6 eq. of O-(6-chlorobenzotriazol-1-yl)-N,N,N′,N′-tetramethyluronium hexafluorophosphate (HCTU, Carl Roth, Germany, c = 0.6 M) in dimethylformamide (DMF, Carl Roth, Germany) in the presence of 12 eq. N,N-

diisopropylethylamine (DIPEA, Iris Biotech, c = 2.4 M) in Nmethylpyrrolidone (NMP, Iris Biotech) for 50 min. Fmoc-protecting groups were removed with a solution of 40 % piperidine (Iris Biotech) in NMP (v/v %) for 5 min and followed by a second deprotection step with 20 % piperidine in NMP (v/v %) for 10 min. After each coupling and deprotection step, the resin was washed with NMP (4 ×). After addition of the final amino acid and deprotection step, the resin was washed with NMP (5 ×), DMF (5 ×) and DCM (5 ×).

For subsequent cyclization, the protected peptide was cleaved with 20 % hexafluoroisopropanol (HFIP, Carbolution Chemicals) for 1 h. The resin was removed byfiltration and the cleavage cocktail was evaporated. The residue was dissolved in DMF (c = 1 mM) and cyclized in solution (micro wave, Discover CEM system, 25 W, 75 °C, 20 min) using 2 eq. HATU, 2 eq. 1-hydroxy-7-azabenzotriazole (HOAt, Carbolution Chemicals) and 4 eq. DIPEA. After evaporation under reduced pressure, the residue was dissolved in DCM and the solution was washed with saturated NaHCO3. The organic layer was dried over MgSO4 and concentrated in vacuo.[2]

2. Supplementary tables

Table S1. ESI-MS data of all produced peptides. The red labeled AAs show a different configuration than expected probably due to a non-functional epimerization domain.

Peptide (#)	mass-to-charge ratio (*m/z*)	Molecular formula	AA sequence	Reference
**1**	586.40	$C_{32}H_{51}O_5N_5$	cyclo(*v*L*fl*L)	[3]
**2**	600.41	$C_{33}H_{53}O_5N_5$	cyclo(*l*L*fl*L)	[3]
**3**	552.41	$C_{29}H_{53}O_5N_5$	cyclo(*v*L*ll*L)	[3]
**4**	566.43	$C_{30}H_{55}O_5N_5$	cyclo(*l*L*ll*L)	[3]
**5**	411.29	$C_{21}H_{38}O_4N_4$	cyclo(*v*L*v*V)	[4]
**6**	231.17	$C_{11}H_{22}O_3N_2$	*v*L	this study
**7**	245.19	$C_{12}H_{24}O_3N_2$	*l*L	this study
**8**	344.25	$C_{17}H_{33}O_4N_3$	*vl*L	this study
**9**	358.28	$C_{18}H_{35}O_4N_3$	*ll*L	this study
**10**	358.28	$C_{18}H_{35}O_4N_3$	*l*LL	this study
**11**	491.32	$C_{26}H_{42}O_5N_4$	*vfl*L	this study
**12**	505.34	$C_{27}H_{44}O_5N_4$	*lfl*L	this study
**13**	457.34	$C_{23}H_{44}O_5N_4$	*vll*L	this study
**14**	471.35	$C_{24}H_{46}O_5N_4$	*lll*L	this study
**15**	457.34	$C_{23}H_{44}O_5N_4$	*v*LLL	this study
**16**	471.35	$C_{24}H_{46}O_5N_4$	*l*LLL	this study
**17**	491.32	$C_{26}H_{42}O_5N_4$	*v*L*f*L	this study
**18**	505.34	$C_{27}H_{44}O_5N_4$	*l*L*f*L	this study
**19**	457.34	$C_{23}H_{44}O_5N_4$	*v*L*l*L	this study
**20**	471.35	$C_{24}H_{46}O_5N_4$	*l*L*l*L	this study
**21**	699.48	$C_{38}H_{62}O_6N_6$	cyclo(*v*LL*fl*L)	this study
**22**	699.48	$C_{38}H_{62}O_6N_6$	cyclo(*v*L*fll*L)	this study
**23**	713.50	$C_{39}H_{64}O_6N_6$	cyclo(*l*L*fll*L)	this study
**24**	665.50	$C_{35}H_{64}O_6N_6$	cyclo(*v*L*lll*L)	this study

**25**	679.51	$C_{36}H_{66}O_6N_6$	cyclo(*l*L*ll*L)	this study
**26**	846.60	$C_{47}H_{71}O_7N_7$	cyclo(*v*L*flfl*L)	this study
**27**	778.58	$C_{41}H_{75}O_7N_7$	cyclo(*v*L*llll*L)	this study
**28**	812.56	$C_{44}H_{73}O_7N_7$	cylco(*v*L*flll*L)	this study
**29**	812.56	$C_{44}H_{73}O_7N_7$	cyclo(*v*L*llfl*L)	this study
**30**	977.64	$C_{53}H_{84}O_9N_8$	*v*L*fl*L*fl*L	this study
**31**	909.67	$C_{47}H_{88}O_9N_8$	*v*L*ll*L*ll*L	this study
**32**	943.66	$C_{50}H_{86}O_9N_8$	*v*L*ll*L*fl*L	this study
**33**	957.67	$C_{51}H_{88}O_9N_8$	*l*L*fl*L*ll*L	this study
**34**	957.67	$C_{51}H_{88}O_9N_8$	*l*L*lf*L*ll*L	this study
**35**	459,30	$C_{25}H_{38}N_4O_4$	cylco(*v*L*f*V)	this study
**36**	425,31	$C_{22}H_{40}N_4O_4$	cyclo(*v*L*l*V)	this study
**37**	778,45	$C_{41}H_{59}N_7O_8$	*v*L*vv*YW	this study
**38**	538.40	$C_{28}H_{51}N_5O_5$	cyclo(*v*L*vl*L)	this study
**39**	552.41	$C_{29}H_{53}N_5O_5$	cyclo(*l*L*vl*L)	this study
**40**	425.31	$C_{22}H_{40}N_4O_4$	cyclo(*l*L*v*V)	this study
**41**	826.45	$C_{45}H_{59}N_7O_8$	*v*L*fv*YW	this study
**42**	840.47	$C_{46}H_{61}N_7O_8$	*l*L*fv*YW	this study
**43**	792.47	$C_{42}H_{61}N_7O_8$	*v*L*lv*YW	this study
**44**	806.48	$C_{43}H_{63}N_7O_8$	*l*L*lv*YW	this study
**45**	792.47	$C_{42}H_{61}N_7O_8$	*l*L*vv*YW	this study
**46**	476.30	$C_{25}H_{40}N_4O_5$	*v*L*f*V	this study
**47**	490.32	$C_{26}H_{42}N_4O_5$	*l*L*f*V	this study
**48**	314.27	$C_{18}H_{35}NO_3$	12:0-L	this study
**49**	328.29	$C_{19}H_{37}NO_3$	13:0-L	this study
**50**	342.20	$C_{20}H_{39}NO_3$	14:0-L	this study
**51**	455.38	$C_{26}H_{50}N_2O_4$	14:0-L*l*	this study
**52**	510.39	$C_{28}H_{51}N_3O_5$	10:0(β-hydroxy)-*l*LL	this study
**53**	496.37	$C_{27}H_{49}N_3O_5$	10:0(β-hydroxy)-*l*LV	this study
**54**	500.20	$C_{26}H_{49}N_3O_6$	10:0(β-hydroxy)-L*v*V	this study
**55**	556.35	$C_{27}H_{49}N_5O_5S$	*l*C*L*l*L	[1]

Table S2. HR-MS data of all synthetic standards.

Peptide (#)	ion formula [H+]	AA sequence	measured m/z	calculated m/z	Δppm
**1**	$C_{32}H_{52}O_5N_5$	cyclo(*v*L*fl*L)	586.3955	586.3963	1.4
**5**	$C_{21}H_{39}O_4N_4$	cyclo(*v*L*v*V)	411.2959	411.2966	1.7
**6**	$C_{11}H_{23}O_3N_2$	*v*L	231.1701	231.1703	0.9
**8**	$C_{17}H_{34}O_4N_3$	*vl*L	344.2540	344.2544	1.0
**10**	$C_{18}H_{36}O_4N_3$	*l*LL	358.2697	358.2700	1.1
**11**	$C_{26}H_{43}O_5N_4$	*vfl*L	491.3228	491.3228	0.1
**15**	$C_{23}H_{45}O_5N_4$	*v*LLL	457.3379	457.3384	1.3
**17**	$C_{26}H_{43}O_5N_4$	*v*L*f*L	491.3231	491.3228	-0.7
**22**	$C_{38}H_{63}O_6N_6$	cyclo(*v*L*fll*L)	699.4793	699.4804	1.5
**26**	$C_{47}H_{72}O_7N_7$	cyclo(*v*L*flfl*L)	846.5464	846.5488	2.8
**32**	$C_{50}H_{87}O_9N_8$	*v*L*ll*L*fl*L	943.6558	943.6518	3.5
**37**	$C_{41}H_{60}N_7O_8$	*v*L*vv*YW	778.4482	778.4498	2.1
**41**	$C_{45}H_{60}N_7O_8$	*v*L*fv*YW	826.4478	826.4498	2.4

Table S3. Theoretical mass-to-charge ratio of non-produced peptides.

Peptide (#)	Theoretical mass-to-charge ratio (*m/z*)	Molecular formula	AA sequence
**56**	473.32	$C_{26}H_{40}N_4O_4$	cyclo(*vfl*L)
**57**	487.32	$C_{27}H_{42}N_4O_4$	cyclo(*lfl*L)
**58**	439.33	$C_{23}H_{42}N_4O_4$	cyclo(*vll*L)
**59**	453.34	$C_{24}H_{44}N_4O_4$	cyclo(*lll*L)
**60**	439.33	$C_{23}H_{42}N_4O_4$	cyclo(*v*LLL)
**61**	453.32	$C_{24}H_{44}N_4O_4$	cyclo(*l*LLL)
**62**	473.32	$C_{26}H_{40}N_4O_4$	cyclo(*v*L*f*L)
**63**	487.32	$C_{27}H_{42}N_4O_4$	cyclo(*l*L*f*L)
**64**	439.33	$C_{23}H_{42}N_4O_4$	cyclo(*v*L*l*L)
**65**	453.34	$C_{24}H_{44}N_4O_4$	cyclo(*l*L*l*L)
**66**	959.64	$C_{53}H_{82}N_8O_8$	cyclo(*v*L*flfl*L)
**67**	891.72	$C_{47}H_{86}N_8O_8$	cyclo(*v*L*llll*L)
**68**	925.63	$C_{50}H_{84}N_8O_8$	cyclo(*v*L*flll*L)
**69**	939.64	$C_{51}H_{86}N_8O_8$	cyclo(*v*L*llfl*L)

Table S4. Strains used in this work.

Strain	Genotype/ NRPS	Reference
E. coli DH10B	F_mcrA (*mrr-hsd*RMS-*mcr*BC), 80*lac*ZΔ, M15, Δ*lac*X74 *rec*A1 *end*A1 *ara*D 139Δ(*ara*, *leu*)7697 *gal*U *gal*K λ *rps*L (*Strr*) *nup*G / -	[5]
E. coli DH10B::*mta*A	DH10B with *mtaA* from pCK_*mtaA*Δ*entD* / -	[6]
P. luminescens TTO1	- / *gxpS*	DSMZ
X. bovienii SS-2004	- / *garS*	[7]
X. nematophila ATCC 19061	- / *xtpS*	ATCC
X. szentirmaii DSM16338	- / *szeS*	DSMZ
X. indica DSM 17382	- / *xldS*	DSMZ

Table S5. Plasmids used in this work.

Plasmids	Genotype	Reference
pCOLA_ara/tacI	ori ColA, kan^R, *araC-P$_{BAD}$* and *tac*I	unpublished
pCK_0402	ori p15A, cm^R, *araC-P$_{BAD}$* and *tac*I-*araE*	[1]
pCOLA_ara_xtpS_tacI_JW	ori ColA, kan^R, *araC-P$_{BAD}$ xtpS* and *tac*I	[1]
pCOLA_ara_gxpS_tacI_JW	ori ColA, kan^R, *araC-P$_{BAD}$ gxpS* and *tac*I	[1]
pNA2	ori p15A, cm^R, *araC-P$_{BAD}$ xtpS*_ $A_1T_1C/E_2A_2T_2$-SYNZIP17 und *tac*I-*araE*	this study

pNA3	ori ColA, kan^R, *araC-P_{BAD}* SYNZIP18-*xtpS*_ $C_3A_3T_3C/E_4A_4T_4TE$ und *tac*I	this study
pNA4	ori p15A, cm^R, *araC-P_{BAD} xtpS*_ A_1T_1C/E_2A_2-SYNZIP17 und *tac*I-*araE*	this study
pNA5	ori ColA, kan^R, *araC-P_{BAD}* SYNZIP18-*xtpS*_ $T_2C_3A_3T_3C/E_4A_4T_4TE$ und *tac*I	this study
pNA15	ori ColA, kan^R, *araC-P_{BAD}* SYNZIP18-*xtpS*_$T_2C_3A_3$-SYNZIP1 and *tac*I	this study
pNA16	ori CloDF13, $spec^R$, *araC-P_{BAD}* SYNZIP2-*xtpS*_$T_3C/E_4A_4T_4TE$ and *tac*I	this study
pNA17	ori ColA, kan^R, *araC-P_{BAD}* SYNZIP18-*xtpS*_$C_3A_3T_4$-SYNZIP1 and *tac*I	this study
pNA18	ori CloDF13, $spec^R$, *araC-P_{BAD}* SYNZIP2-*xtpS*_$C/E_4A_4T_4TE$ and *tac*I	this study
pNA24	ori ColA, kan^R, *araC-P_{BAD}* SYNZIP18-*xldS*_$T_2C_3A_3$-SYNZIP1 and *tac*I	this study
pNA26	ori p15A, cm^R, *araC-P_{BAD} gxpS*_A_1T_1C/E_2A_2-SYNZIP17 and *tac*I-*araE*	this study
pNA27	ori ColA, kan^R, *araC-P_{BAD}* SYNZIP18-*gxpS*_$T_2C_3A_3$-SYNZIP1 and *tac*I	this study
pNA28	ori CloDF13, $spec^R$, *araC-P_{BAD}* SYNZIP2-*gxpS*_$T_3C/E_4A_4T_4$ $C/E_5A_5T_5TE$ and *tac*I	this study
pNA29	ori p15A, cm^R, *araC-P_{BAD} sze*S_ $C_1A_1T_1C/E_2A_2$-SYNZIP17 and *tac*I-*araE*	this study
pNA30	ori ColA, kan^R, *araC-P_{BAD}* SYNZIP18-*szeS*_$T_2C_3A_3$-SYNZIP1 and *tac*I	this study
pNA31	ori CloDF13, $spec^R$, *araC-P_{BAD}* SYNZIP2-*szeS*_$T_3C/E_4A_4T_4$ $C/E_5A_5T_5C_6A_6T_6TE$ and *tac*I	this study
pNA35	ori ColA, kan^R, *araC-P_{BAD}* SYNZIP18-*garS*_$T_2C_3A_3$-SYNZIP1 and *tac*I	this study
pNA104	ori p15A, cm^R, *araC-P_{BAD} gxpS*_ A_1T_1C/E_2-SYNZIP17 und *tac*I-*araE*	this study
pNA105	ori ColA, kan^R, *araC-P_{BAD}* SYNZIP18-*gxpS*_ $A_2T_2C_3A_3T_3C/E_4A_4T_4C/E_5A_5T_5TE$ und *tac*I	this study
pNA106	ori p15A, cm^R, *araC-P_{BAD} gxpS*_ $A_1T_1C/E_2A_2T_2C_3A_3T_3C/E_4$-SYNZIP17 und *tac*I-*araE*	this study
pNA107	ori ColA, kan^R, *araC-P_{BAD}* SYNZIP18-*gxpS*_ $A_4T_4C/E_5A_5T_5TE$ und *tac*I	this study
pNA108	ori p15A, cm^R, *araC-P_{BAD} gxpS*_ $A_1T_1C/E_2A_2T_2C_3A_3T_3C/E_4A_4T_4C/E_5$-SYNZIP17 und *tac*I-*araE*	this study
pNA109	ori ColA, kan^R, *araC-P_{BAD}* SYNZIP18-*gxpS*_A_5T_5TE und *tac*I	this study
pNA59	ori p15A, cm^R, *araC-P_{BAD} xfpS*_C_1A_1-SYNZIP17 and *tac*I-*araE*	this study
pNA60	ori ColA, kan^R, *araC-P_{BAD}* SYNZIP18-*xfpS*_$T_1E_1C_2A_2T_2C_3A_3T_3TE$ and *tac*I	this study
pJW61	ori p15A, cm^R, *araC-P_{BAD} xtpS*_$A_1T_1C/E_2A_2T_2C_3$-SYNZIP17 and *tac*I-*araE*	[1]
pJW62	ori ColA, kan^R, *araC-P_{BAD}* SYNZIP18-*xtpS*_$A_3T_3C/E_4A_4T_4TE$ and *tac*I	[1]
pJW118	ori p15A, cm^R, *araC-P_{BAD} bacA*_A1T1CyA2T2C3A3-SYNZIP17 and *tac*I-*araE*	this study

pJW120	ori ColA, kan^R, *araC-*P_{BAD} SYNZIP18-*bacA*_T3C$_{Dsub}$4-*sfrA-BC*_C$_{Asub}$6A6T6E6C7A7T7TE and *tacI*	this study
pJW122	ori p15A, cm^R, *araC-*P_{BAD} *bacA*_A1T1CyA2T2C3A3T3-SYNZIP17 and *tacI*-*araE*	this study
pJW124	ori ColA, kan^R, *araC-*P_{BAD} SYNZIP18-*bacA*_C$_{Dsub}$4-*sfrA-BC*_C$_{Asub}$6A6T6E6C7A7T7TE and *tacI*	this study
pJW126	ori p15A, cm^R, *araC-*P_{BAD} *bacA*_A1T1CyA2T2C3A3T3 C$_{Dsub}$4-SYNZIP17 and *tacI*-*araE*	this study
pJW128	ori ColA, kan^R, *araC-*P_{BAD} SYNZIP18-*sfrA-BC*_C$_{Asub}$6A6T6E6C7A7T7TE and *tacI*	this study
pJW141	ori p15A, cm^R, *araC-*P_{BAD} *xldS*_C$_1$-SYNZIP17 and *tacI*-*araE*	this study

Table S6. Oligonucleotides used in this work.

Plasmids	Oligo-nucleotides	Sequence (5' →3'; overlapping ends)	Template
pNA2	KB-pACYC-II-FW	AACGAGAAGGAGGAATTAAAATCG	pJW61
	KB-pACYC-II-RV	CATGGAATTCCTCCTGTTAGC	pJW61
	na03_FW	TGGGCTAACAGGAGGAATTCCATGAAAGATAGCATGGCTAAAAAGGG	*X. nematophila* ATCC 19061
	na05_RV	CGATTTTAATTCCTCCTTCTCGTTAACACGATCACGGGATATTG	*X. nematophila* ATCC 19061
pNA3	KB-pCOLA-II-FW	TGACAATTAATCATCGGCTCG	pJW62
	KB-pCOLA-II-RV	TGAGATAGCTGCAGTCAGCTCG	pJW62
	na06_FW	AACGAGCTGACTGCAGCTATCTCATTGCCTTTATCGTTTGGTCAAC	*X. nematophila* ATCC 19061
	na07_RV	CGAGCCGATGATTAATTGTCACAGCGCCTCCACTTCG	*X. nematophila* ATCC 19061
pNA4	KB-pACYC-II-FW	AACGAGAAGGAGGAATTAAAATCG	pJW61
	KB-pACYC-II-RV	CATGGAATTCCTCCTGTTAGC	pJW61
	na03_FW	TGGGCTAACAGGAGGAATTCCATGAAAGATAGCATGGCTAAAAAGGG	*X. nematophila* ATCC 19061
	na13_RV	CGATTTTAATTCCTCCTTCTCGTTATAAATCTGGCGGGCGAA	*X. nematophila* ATCC 19061
pNA5	KB-pCOLA-II-FW	TGACAATTAATCATCGGCTCG	pJW62
	KB-pCOLA-II-RV	TGAGATAGCTGCAGTCAGCTCG	pJW62
	na14_FW	AACGAGCTGACTGCAGCTATCTCAGTTGCGCCACAAGGAGAA	*X. nematophila* ATCC 19061
	na07_RV	CGAGCCGATGATTAATTGTCACAGCGCCTCCACTTCG	*X. nematophila* ATCC 19061
pNA15	na28_FW	CACAAAAAAGACCTGATCGCGTACCTGGAGAAAGAAATCGCGA ATCTGCGTAAGAAAATCGAAGAATGACAATTAATCATCGGCTCG	pJW62
	na29_FW	AACCTGGTTGCGCAGCTCGAAAACGAAGTTGCGTCTCTGGAAAATGAGA ACGAAACCCTGAAGAAAAAGAACCTGCACAAAAAAGACCTGATCGCGTAC	pCOLA_ara/tacI_SZ18_ halfSZ1
	na30_RV	TGAGATAGCTGCAGTCAGCTCG	pJW62/ pCOLA_ara/tacI_SZ18_ halfSZ1
	na14_FW	GGCTAACAGGAGGAATTCCATGGTTGCGCCACAAGGAGAA	*X. nematophila* ATCC 19061
	na31_RV	AGCTGCGCAACCAGGTTATAGACCTGCCGGGCAAAC	*X. nematophila* ATCC 19061
pNA16	na32_RV	GTTCTGTTCATCACGTTCCAGCTGCAGGTTGTCTTTTTTCAGACGTGCGA TTTTCTTACGCAGATACGCGTTACGCGCCATGGAATTCCTCCTGTTAGCC	pCDF_ara/tacI
	na33_RV	CTGTTCGTGAGACGCAACTTCGTTTTCGAGACGCGCGATTTCGTCAC GCAGGTTCGCGATGATTTTTTCCAGGTTCTGTTCATCACGTTCCAGC	pCDF_ara/tacI_halbSZ2
	na34_FW	TGACAATTAATCATCGGCTCG	pCDF_ara/tacI/ pCDF_ara/tacI_halbSZ2

	na35_FW	AAACGAAGTTGCGTCTCACGAACAGGCGGCTCCGCAGGG	*X. nematophila* ATCC 19061
	na7_RV	CGAGCCGATGATTAATTGTCACAGCGCCTCCACTTCG	*X. nematophila* ATCC 19061
pNA17	na28_FW	CACAAAAAAGACCTGATCGCGTACCTGGAGAAAGAAATCGCGA ATCTGCGTAAGAAAATCGAAGAATGACAATTAATCATCGGCTCG	pJW62
	na29_FW	AACCTGGTTGCGCAGCTCGAAAACGAAGTTGCGTCTCTGGAAAATGAGA ACGAAACCCTGAAGAAAAAGAACCTGCACAAAAAAGACCTGATCGCGTAC	pCOLA_ara/tacI_SZ18_ halfSZ1
	na30_RV	TGAGATAGCTGCAGTCAGCTCG	pJW62/ pCOLA_ara/tacI_SZ18_ halfSZ1
	na36_FW	AACGAGCTGACTGCAGCTATCTCATTGCCTTTATCGTTTGGTCAACAG	*X. nematophila* ATCC 19061
	na37_RV	CGTTTTCGAGCTGCGCAACCAGGTTCATGGCTGGCGTTAGTACCG	*X. nematophila* ATCC 19061
pNA18	na32_RV	GTTCTGTTCATCACGTTCCAGCTGCAGGTTGTCTTTTTTCAGACGTGCGA TTTTCTTACGCAGATACGCGTTACGCGCCATGGAATTCCTCCTGTTAGCC	pCDF_ara/tacI
	na33_RV	CTGTTCGTGAGACGCAACTTCGTTTTCGAGACGCGCGATTTCGTCAC GCAGGTTCGCGATGATTTTTTCCAGGTTCTGTTCATCACGTTCCAGC	pCDF_ara/tacI_halbSZ2
	na34_FW	TGACAATTAATCATCGGCTCG	pCDF_ara/tacI/ pCDF_ara/tacI_halbSZ2
	na38_FW	AAACGAAGTTGCGTCTCACGAACAGTTGCCGCTGATTGATCTCAC	*X. nematophila* ATCC 19061
	na7_RV	CGAGCCGATGATTAATTGTCACAGCGCCTCCACTTCG	*X. nematophila* ATCC 19061
pNA24	na28_FW	CACAAAAAAGACCTGATCGCGTACCTGGAGAAAGAAATCGCGA ATCTGCGTAAGAAAATCGAAGAATGACAATTAATCATCGGCTCG	pJW62
	na29_FW	AACCTGGTTGCGCAGCTCGAAAACGAAGTTGCGTCTCTGGAAAATGAGA ACGAAACCCTGAAGAAAAAGAACCTGCACAAAAAAGACCTGATCGCGTAC	pCOLA_ara/tacI_SZ18_ halfSZ1
	na30_RV	TGAGATAGCTGCAGTCAGCTCG	pJW62/ pCOLA_ara/tacI_SZ18_ halfSZ1
	na47_FW	AACGAGCTGACTGCAGCTATCTCAGAAGCGCCCATTGGCAAA	*X. indica* DSM 17382
	na48_RV	CGTTTTCGAGCTGCGCAACCAGGTTATAGCCACGTGTAACAACCGCTG	*X. indica* DSM 17382
pNA26	KB-pACYC-II-FW	AACGAGAAGGAGGAATTAAAATCG	pJW61
	KB-pACYC-II-RV	CATGGAATTCCTCCTGTTAGC	pJW61
	na51	GCTAACAGGAGGAATTCCATGAAAGATAGCATGGCTAAAAAGGAAAT	*P. luminescens* TT01
	na52	CGATTTTAATTCCTCCTTCTCGTTATAAATTTGGCGAGCAAAAGC	*P. luminescens* TT01
pNA27	na28_FW	CACAAAAAAGACCTGATCGCGTACCTGGAGAAAGAAATCGCGA ATCTGCGTAAGAAAATCGAAGAATGACAATTAATCATCGGCTCG	pJW62
	na29_FW	AACCTGGTTGCGCAGCTCGAAAACGAAGTTGCGTCTCTGGAAAATGAGA ACGAAACCCTGAAGAAAAAGAACCTGCACAAAAAAGACCTGATCGCGTAC	pCOLA_ara/tacI_SZ18_ halfSZ1
	na30_RV	TGAGATAGCTGCAGTCAGCTCG	pJW62/ pCOLA_ara/tacI_SZ18_ halfSZ1
	na53	CGAGCTGACTGCAGCTATCTCAGTCGCGCCACAGGGAG	*P. luminescens* TT01
	na534	CGTTTTCGAGCTGCGCAACCAGGTTGTAAGCTTGGCGAGCAAAGG	*P. luminescens* TT01
pNA28	na32_RV	GTTCTGTTCATCACGTTCCAGCTGCAGGTTGTCTTTTTTCAGACGTGCGA TTTTCTTACGCAGATACGCGTTACGCGCCATGGAATTCCTCCTGTTAGCC	pCDF_ara/tacI
	na33_RV	CTGTTCGTGAGACGCAACTTCGTTTTCGAGACGCGCGATTTCGTCAC GCAGGTTCGCGATGATTTTTTCCAGGTTCTGTTCATCACGTTCCAGC	pCDF_ara/tacI_halbSZ2
	na34_FW	TGACAATTAATCATCGGCTCG	pCDF_ara/tacI/ pCDF_ara/tacI_halbSZ2
	na55	GAAGTTGCGTCTCACGAACAGCAAGCGCCACAAGGGGA	*P. luminescens* TT01
	na56	CGAGCCGATGATTAATTGTCACAGCGCCTCCGCTTCAC	*P. luminescens* TT01
pNA29	KB-pACYC-II-FW	AACGAGAAGGAGGAATTAAAATCG	pJW61
	KB-pACYC-II-RV	CATGGAATTCCTCCTGTTAGC	pJW61
	na57	GCTAACAGGAGGAATTCCATGAAAGGTAGTATTGCTAAAAAGGGAGATG	*X. szentirmaii* DSM16338
	na58	CGATTTTAATTCCTCCTTCTCGTTATAATGCTGACGGGCAAATG	*X. szentirmaii* DSM16338
pNA30	na28_FW	CACAAAAAAGACCTGATCGCGTACCTGGAGAAAGAAATCGCGA ATCTGCGTAAGAAAATCGAAGAATGACAATTAATCATCGGCTCG	pJW62
	na29_FW	AACCTGGTTGCGCAGCTCGAAAACGAAGTTGCGTCTCTGGAAAATGAGA ACGAAACCCTGAAGAAAAAGAACCTGCACAAAAAAGACCTGATCGCGTAC	pCOLA_ara/tacI_SZ18_ halfSZ1
	na30_RV	TGAGATAGCTGCAGTCAGCTCG	pJW62/ pCOLA_ara/tacI_SZ18_ halfSZ1

	na59	CGAGCTGACTGCAGCTATCTCAGAATCCCCACAAGGGGAGA	*X. szentirmaii* DSM16338
	na60	TCGAGCTGCGCAACCAGGTTATAATGCTGACGGGCAAACG	*X. szentirmaii* DSM16338
pNA31	na32_RV	GTTCTGTTCATCACGTTCCAGCTGCAGGTTGTCTTTTTTCAGACGTGCGA TTTTCTTACGCAGATACGCGTTACGCGCCATGGAATTCCTCCTGTTAGCC	pCDF_ara/tacI
	na33_RV	CTGTTCGTGAGACGCAACTTCGTTTTCGAGACGCGCGATTTCGTCAC GCAGGTTCGCGATGATTTTTTCCAGGTTCTGTTCATCACGTTCCAGC	pCDF_ara/tacI_halbSZ2
	na34_FW	TGACAATTAATCATCGGCTCG	pCDF_ara/tacI/ pCDF_ara/tacI_halbSZ2
	na61	AAAACGAAGTTGCGTCTCACGAACAGGAGTTGCCACAAGGTGAAA	*X. szentirmaii* DSM16338
	na62	CGAGCCGATGATTAATTGTCAAATATTATTTACTATATTGTATTCCTCTGTACCA	*X. szentirmaii* DSM16338
pNA35	na28_FW	CACAAAAAAGACCTGATCGCGTACCTGGAGAAAGAAATCGCGA ATCTGCGTAAGAAAATCGAAGAATGACAATTAATCATCGGCTCG	pJW62
	na29_FW	AACCTGGTTGCGCAGCTCGAAAACGAAGTTGCGTCTCTGGAAAATGAGA ACGAAACCCTGAAGAAAAAGAACCTGCACAAAAAAGACCTGATCGCGTAC	pCOLA_ara/tacI_SZ18_ halfSZ1
	na30_RV	TGAGATAGCTGCAGTCAGCTCG	pJW62/ pCOLA_ara/tacI_SZ18_ halfSZ1
	na68	TAACGAGCTGACTGCAGCTATCTCAGAAACTCCGGTCGGTAAAGTAG	*X. bovienii* SS-2004
	na69	TTCGTTTTCGAGCTGCGCAACCAGGTTATAGCCGCGCACCACTAC	*X. bovienii* SS-2004
pNA59	KB-pACYC-FW	GAACAGTTAAAACAGAAGCGTGAACAATTAAAGCAAAAGATCGCCAATC TGCGTAAGGAGATCGAAGCCTACAAGTGACAATTAATCATCGGCTCG	pCK_0402
	KB-pACYC-RV	TTCACGCTTCTGTTTTAACTGTTCGATGCGATTACGCAATTCAGCCTTT TTCGATTTTAATTCCTCCTTCTCGTTCATGGAATTCCTCCTGTTAGC	pCK_0402
	na125_FW	GCTAACAGGAGGAATTCCATGGATAACATTCTGGCCTCG	*X. bovienii* SS2004
	na126_RV	CAGATTTTAAACAGAGCCGCTATGTTTATAACGAGAAGGAGGAATTAAAATCG	*X. bovienii* SS2004
pNA60	KB-pCOLA-FW	CATTGACAAAGAGCTGCGTGCCAACGAAAACGAACTTCGCGCCCTTGA TAACGAGCTGACTGCAGCTATCTCATGACAATTAATCATCGGCTCG	pCOLA_ara/tacI
	KB-pCOLA-RV	TTGGCACGCAGCTCTTTGTCAATGGCATTTAACTCGCGGTCCAAGGCT TTCAGTTCACGCTCTTCAGCATAGAACATGGAATTCCTCCTGTTAGC	pCOLA_ara/tacI
	na127_FW	AACGAGCTGACTGCAGCTATCTCACGTGCCGCAGAAACGG	*X. bovienii* SS2004
	na128_RV	ATACGAGCCGATGATTAATTGTCAATCCACCAGCTCCAACAC	*X. bovienii* SS2004
pNA104	KB-pACYC-II-FW	AACGAGAAGGAGGAATTAAAATCG	pJW61
	KB-pACYC-II-RV	CATGGAATTCCTCCTGTTAGC	pJW61
	na194_FW	GGCTAACAGGAGGAATTCCATGAAAGATAGCATGGCTAAAAAGG	*P. luminescens* TT01
	na208_RV	CGATTTTAATTCCTCCTTCTCGTTCCACGTTTCCAGCAACAAC	*P. luminescens* TT01
pNA105	KB-pCOLA-II-FW	TGACAATTAATCATCGGCTCG	pJW62
	KB-pCOLA-II-RV	TGAGATAGCTGCAGTCAGCTCG	pJW62
	na209_FW	CGAGCTGACTGCAGCTATCTCAAACGCGACAGAAACCCC	*P. luminescens* TT01
	na56_RV	CGAGCCGATGATTAATTGTCACAGCGCCTCCGCTTCAC	*P. luminescens* TT01
pNA106	KB-pACYC-II-FW	AACGAGAAGGAGGAATTAAAATCG	pJW61
	KB-pACYC-II-RV	CATGGAATTCCTCCTGTTAGC	pJW61
	na194_FW	GGCTAACAGGAGGAATTCCATGAAAGATAGCATGGCTAAAAAGG	*P. luminescens* TT01
	na210_RV	CGATTTTAATTCCTCCTTCTCGTTCCAAGTTTTTAACAACAATGTGCG	*P. luminescens* TT01
pNA107	KB-pCOLA-II-FW	TGACAATTAATCATCGGCTCG	pJW62
	KB-pCOLA-II-RV	TGAGATAGCTGCAGTCAGCTCG	pJW62
	na211_FW	CGAGCTGACTGCAGCTATCTCAAACGCCACTGAAACAGCCTATC	*P. luminescens* TT01
	na56_RV	CGAGCCGATGATTAATTGTCACAGCGCCTCCGCTTCAC	*P. luminescens* TT01
pNA108	KB-pACYC-II-FW	AACGAGAAGGAGGAATTAAAATCG	pJW61
	KB-pACYC-II-RV	CATGGAATTCCTCCTGTTAGC	pJW61
	na194_FW	GGCTAACAGGAGGAATTCCATGAAAGATAGCATGGCTAAAAAGG	*P. luminescens* TT01

	na212_RV	CGATTTTAATTCCTCCTTCTCGTTGCCGACTTTCAGTAACAGTTTCC	*P. luminescens* TT01
pNA109	KB-pCOLA-II-FW	TGACAATTAATCATCGGCTCG	pJW62
	KB-pCOLA-II-RV	TGAGATAGCTGCAGTCAGCTCG	pJW62
	na213_FW	CGAGCTGACTGCAGCTATCTCAAATGGCCCGCAAACGG	*P. luminescens* TT01
	na56_RV	CGAGCCGATGATTAATTGTCACAGCGCCTCCGCTTCAC	*P. luminescens* TT01
pJW118	KB-pACYC-II-FW	AACGAGAAGGAGGAATTAAAATCG	pJW61
	KB-pACYC-II-RV	CATGGAATTCCTCCTGTTAGC	pJW61
	jw208_FW	GCTAACAGGAGGAATTCCATGGTTGCTAAACATTCATTAGAAAATGGG	pFF1_NRPS_6 (*3*)
	jw0214_RV	CGATTTTAATTCCTCCTTCTCGTTGTAGCGGCGATCCATTGT	pFF1_NRPS_6 (*3*)
pJW120	KB-pCOLA-II-FW	TGACAATTAATCATCGGCTCG	pJW62
	KB-pCOLA-II-RV	TGAGATAGCTGCAGTCAGCTCG	pJW62
	jw0216_FW	CGAGCTGACTGCAGCTATCTCAGAAGCGCCGCGGG	pFF1_NRPS_6 (*3*)
	jw0212_RV	CGAGCCGATGATTAATTGTCATGAAACCGTTACGGTTTGTGTATTA	pFF1_NRPS_6 (*3*)
pJW122	KB-pACYC-II-FW	AACGAGAAGGAGGAATTAAAATCG	pJW61
	KB-pACYC-II-RV	CATGGAATTCCTCCTGTTAGC	pJW61
	jw208_FW	GCTAACAGGAGGAATTCCATGGTTGCTAAACATTCATTAGAAAATGGG	pFF1_NRPS_6 (*3*)
	jw0218_RV	CGATTTTAATTCCTCCTTCTCGTTTCGGTAATATGGTTTTTCTTCGG	pFF1_NRPS_6 (*3*)
pJW124	KB-pCOLA-II-FW	TGACAATTAATCATCGGCTCG	pJW62
	KB-pCOLA-II-RV	TGAGATAGCTGCAGTCAGCTCG	pJW62
	jw0220_FW	CGAGCTGACTGCAGCTATCTCA TTGTCTTCAGCGCAAAAAAGG	pFF1_NRPS_6 (*3*)
	jw0212_RV	CGAGCCGATGATTAATTGTCA TGAAACCGTTACGGTTTGTGTATTA	pFF1_NRPS_6 (*3*)
pJW126 (NRPS-42)	KB-pACYC-II-FW	AACGAGAAGGAGGAATTAAAATCG	pJW61
	KB-pACYC-II-RV	CATGGAATTCCTCCTGTTAGC	pJW61
	jw208_FW	GCTAACAGGAGGAATTCCATGGTTGCTAAACATTCATTAGAAAATGGG	pFF1_NRPS_6 (*3*)
	jw0222_RV	CGATTTTAATTCCTCCTTCTCGTTGGCATGGCTATTTTCCCATT	pFF1_NRPS_6 (*3*)
pJW128	KB-pCOLA-II-FW	TGACAATTAATCATCGGCTCG	pJW62
	KB-pCOLA-II-RV	TGAGATAGCTGCAGTCAGCTCG	pJW62
	jw0224_FW	CGAGCTGACTGCAGCTATCTCACAAAAAGAACGGATGAAGGAGC	pFF1_NRPS_6 (*3*)
	jw0212_RV	CGAGCCGATGATTAATTGTCATGAAACCGTTACGGTTTGTGTATTA	pFF1_NRPS_6 (*3*)
pJW141	KB-pACYC-II-FW	AACGAGAAGGAGGAATTAAAATCG	pJW61
	KB-pACYC-II-RV	CATGGAATTCCTCCTGTTAGC	pJW61
	jw0166_FW	GCTAACAGGAGGAATTCCATGAAACTTTGGAACTATAAAATGAATATGAC	*X. indica* DSM 17382
	jw0254_RV	CGATTTTAATTCCTCCTTCTCGTTAAAATCTACCAATAGTTTCTGGCGC	*X. indica* DSM 17382

Table S7. C-A interface identity. WT interface was sequenced aligned with the artificial interface of NRPS-1-16. Identity is depicted in [%].

NRPS	WT interface	Artificial interface	Identity [%]
NRPS-1	C/E_5-A_5	C/E_2-A_5	89.3
NRPS-2	C/E_4-A_4	C/E_2-A_4	86.1
NRPS-3	C/E_5-A_5	C/E_3-A_5	59.5
NRPS-4	C_3-A_3	C/E_2-A_4	57.9
NRPS-5	C/E_4-A_4	C_3-A_4	59.8
NRPS-6	C/E_5-A_5	C/E_4-A_5	89.3
NRPS-7	C/E_2-A_2	C_3-A_2	58.7
NRPS-8	C_3-A_3	C/E_4-A_3	58.7
NRPS-9	C/E_4-A_4	C/E_5-A_4	89.3
NRPS-10	C/E_2-A_2	C/E_4-A_2	85.2
NRPS-11	C_3-A_3	C/E_5-A_3	59.5
NRPS-12	C/E_5-A_2	C/E_2-A_2	88.5

Table S8. Peptide sequences of applied SZ pairs SZ17:18 and SZ1:2

**SYNZIP17:18**	
heptad position	fg abcdefg abcdefg abcdefg abcdefg abcdefg abcde
SZ17	NE KEELKSK KAELRNR IEQLKQK REQLKQK IANLRKE IEAYK
SZ18reverse	FY AEERELK ALDRELN AIDKELR ANENELR ALDNELT AAIS
**SYNZIP1:2**	
heptad position	fg abcdefg abcdefg abcdefg abcdefg abcdefg abcdefg abcdef
SZ1	NL VAQLENE VASLENE NETLKKK NLHKKDL IAYLEKE IANLRKK IEE
SZ2	AR NAYLRKK IARLKKD NLQLERD EQNLEKI IANLRDE IARLENE VASHEQ

3. Supplementary Figures

Peptide	Peptide Sequence	Production (mg l^{-1})
**1**	cyclo(vfLlL)	6.0 ± 0.5
**2**	cyclo(lfLlL)	0.7 ± 0.1
**3**	cyclo(vlLlL)	2.4 ± 0.3
**4**	cyclo(llLlL)	0.20 ± 0.04

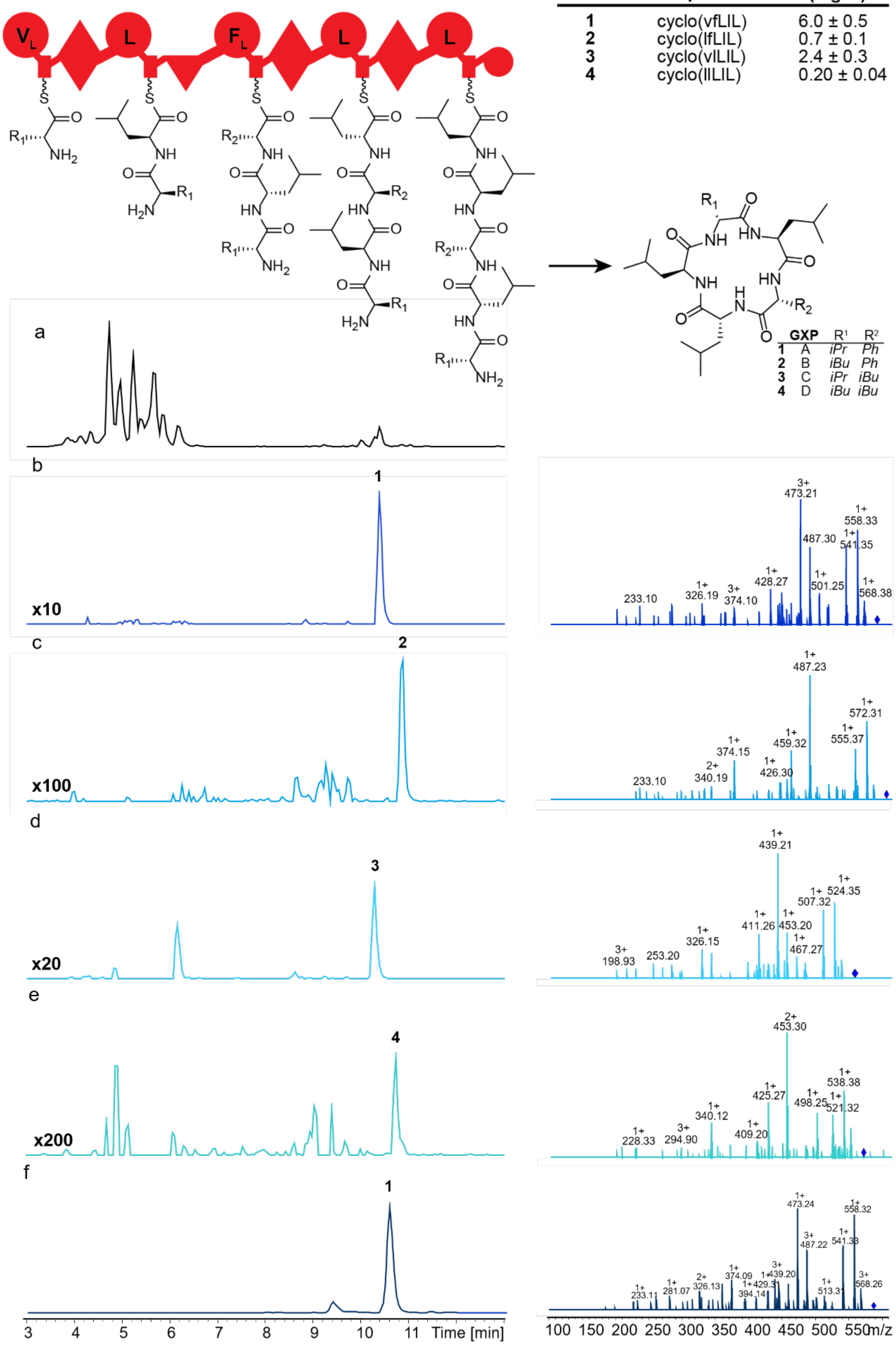

Figure S1. Schematic representation of the GameXPeptide (1-4) producing type A NRPS (GxpS). For domain assignment the following symbols are used: A, adenylation domain, large circles, T, thiolation domain, rectangle, C, condensation domain, triangle, C/E, dual condensation/epimerization

domain, diamond, TE, thioesterase domain, small circle. (a) Base Peak Chromatogram (BPC) of an exemplary culture extract (b) Extracted ion chromatogram (EIC)/MS^2 of **1** (*m/z* $[M+H]^+$ = 586.40). (c) EIC data of **2** (*m/z* $[M+H]^+$ = 600.41). (d) EIC data of **3** (*m/z* $[M+H]^+$ = 552.41). (e) EIC data of **4** (*m/z* $[M+H]^+$ = 566.43). (f) EIC/MS^2 of synthetic **1** (*m/z* $[M+H]^+$ = 586.40).

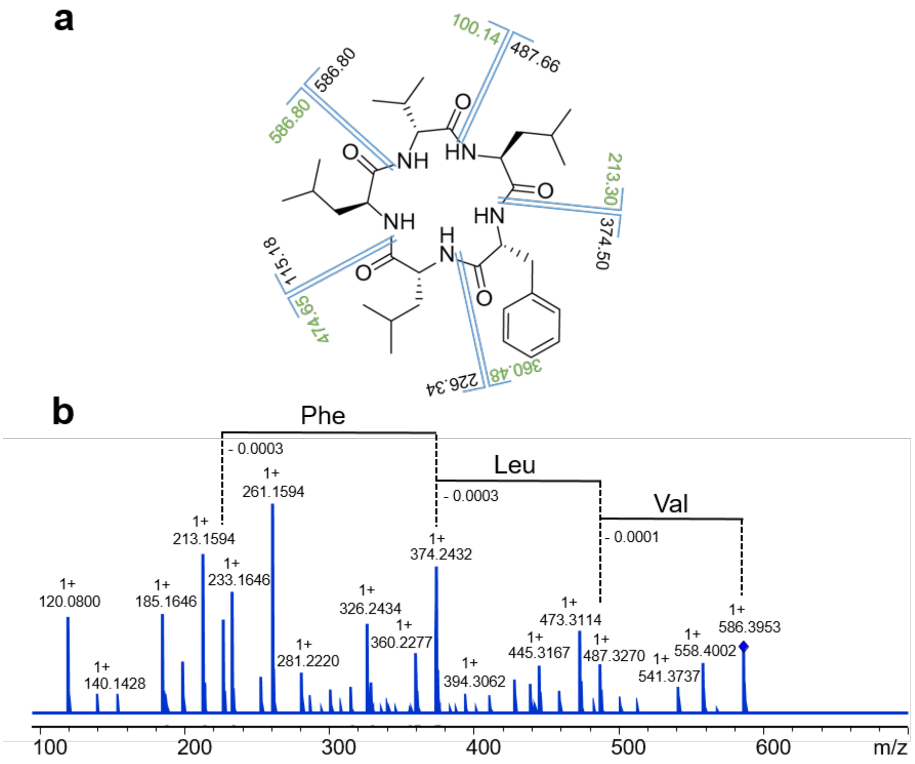

Figure S2. Fragmentation pattern of synthetic 1. (a) Structure of **1** with indicated fragment ions. (b) Fragmentation pattern of **1.**

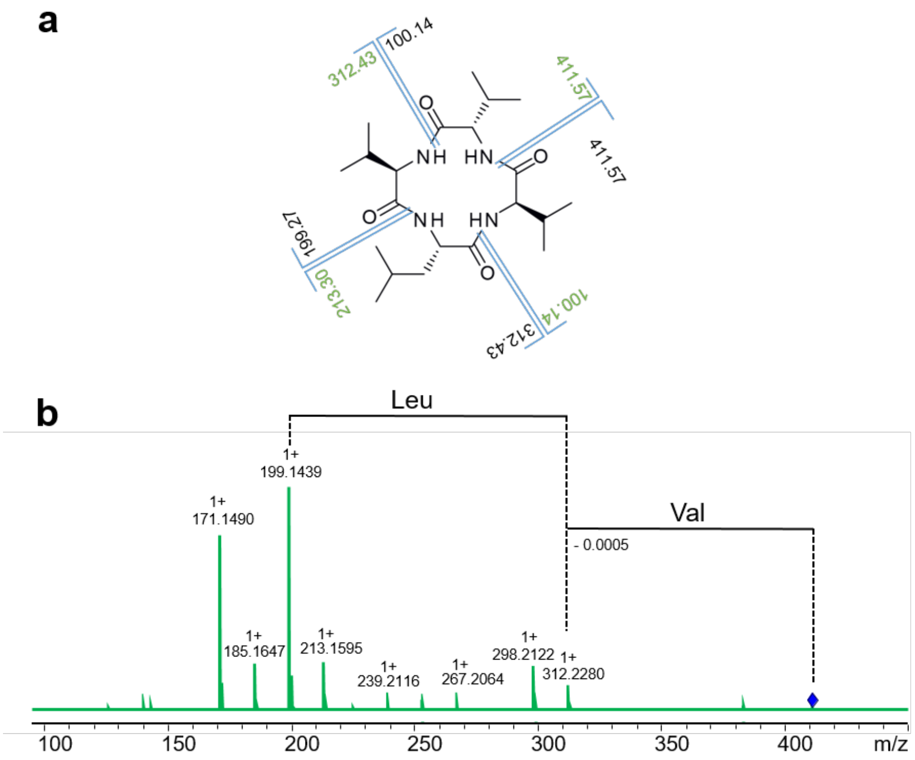

Figure S3. Fragmentation pattern of synthetic 5. (a) Structure of **5** with indicated fragment ions. (b) Fragmentation pattern of **5.**

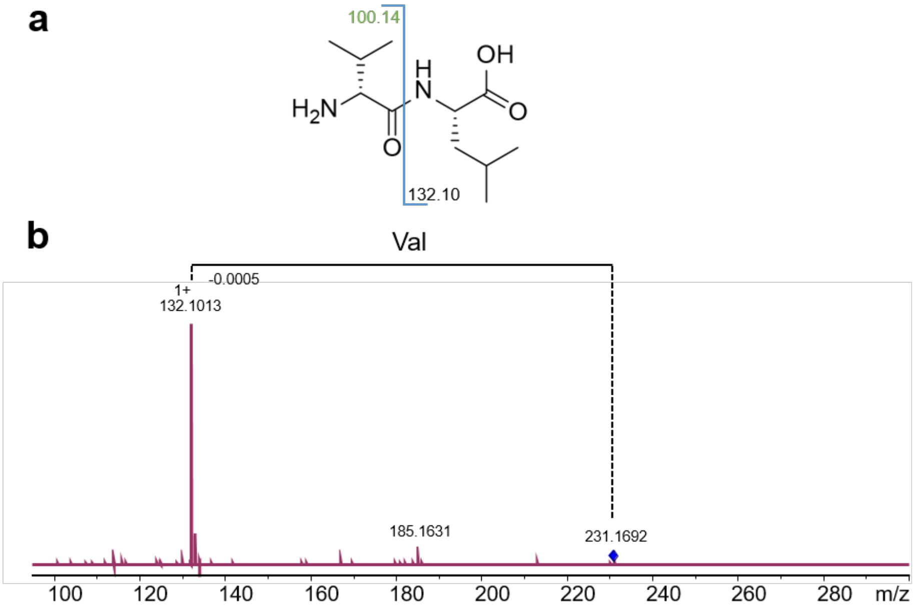

Figure S4. Fragmentation pattern of synthetic 6. (a) Structure of **6** with indicated fragment ions. (b) Fragmentation pattern of **6.**

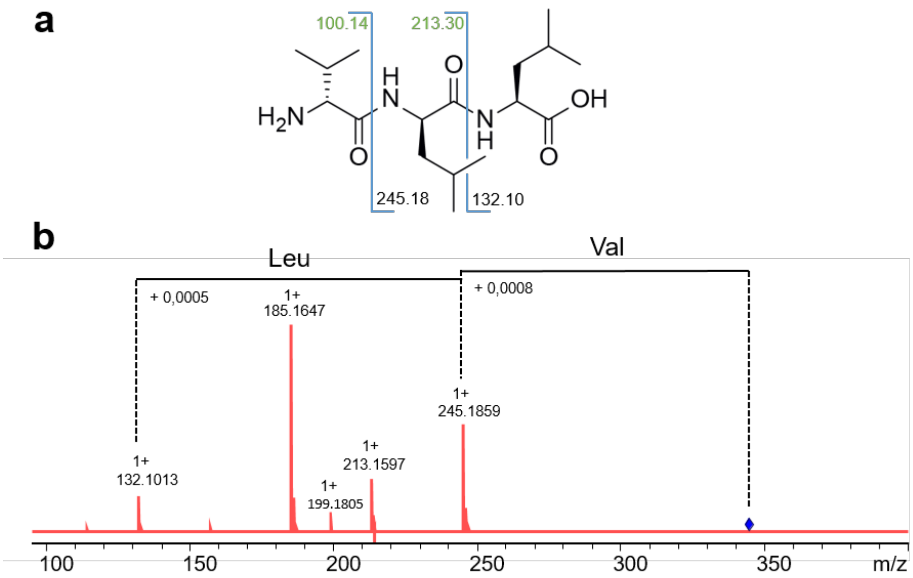

Figure S5. Fragmentation pattern of synthetic 8. (a) Structure of **8** with indicated fragment ions. (b) Fragmentation pattern of **8.**

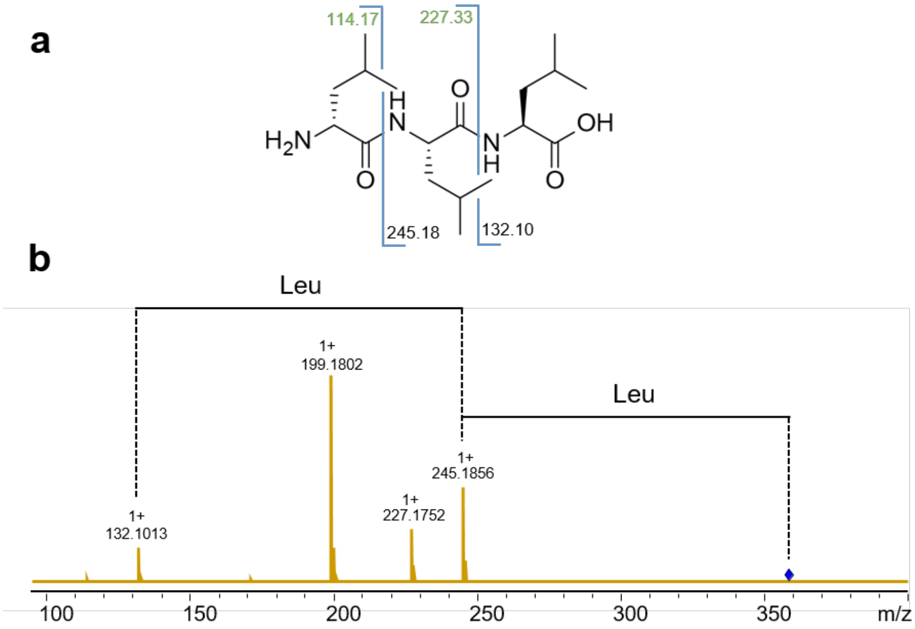

Figure S6. Fragmentation pattern of synthetic 10. (a) Structure of **10** with indicated fragment ions. (b) Fragmentation pattern of **10.**

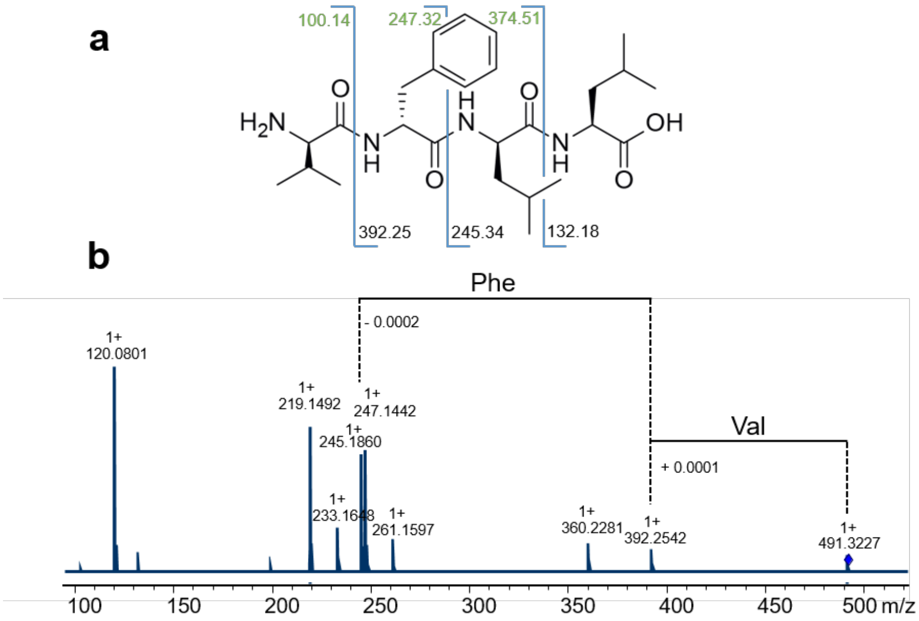

Figure S7. Fragmentation pattern of synthetic 11. (a) Structure of **11** with indicated fragment ions. (b) Fragmentation pattern of **11.**

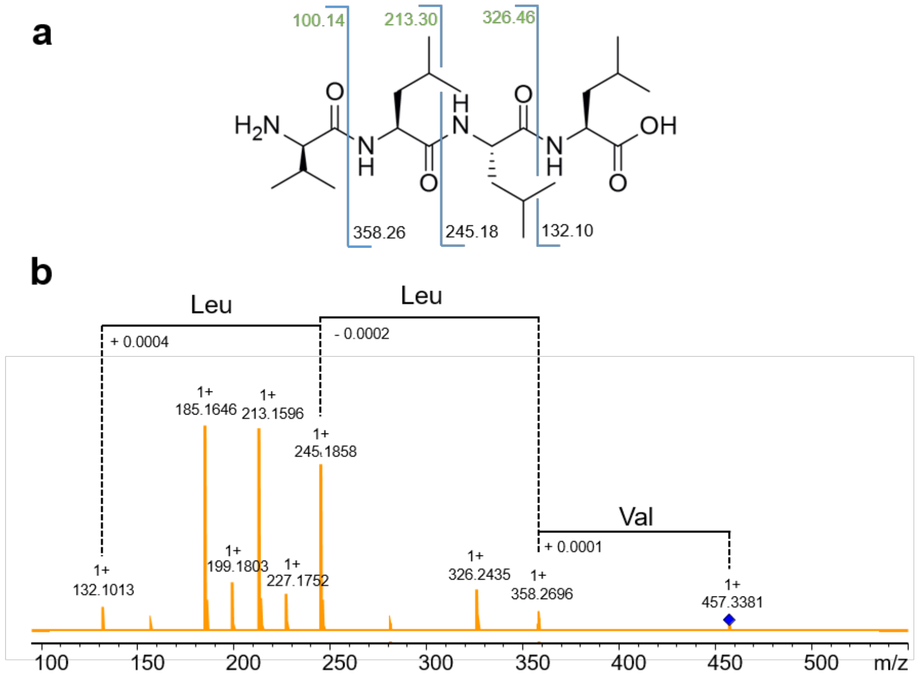

Figure S8. Fragmentation pattern of synthetic 15. (a) Structure of **15** with indicated fragment ions. (b) Fragmentation pattern of **15.**

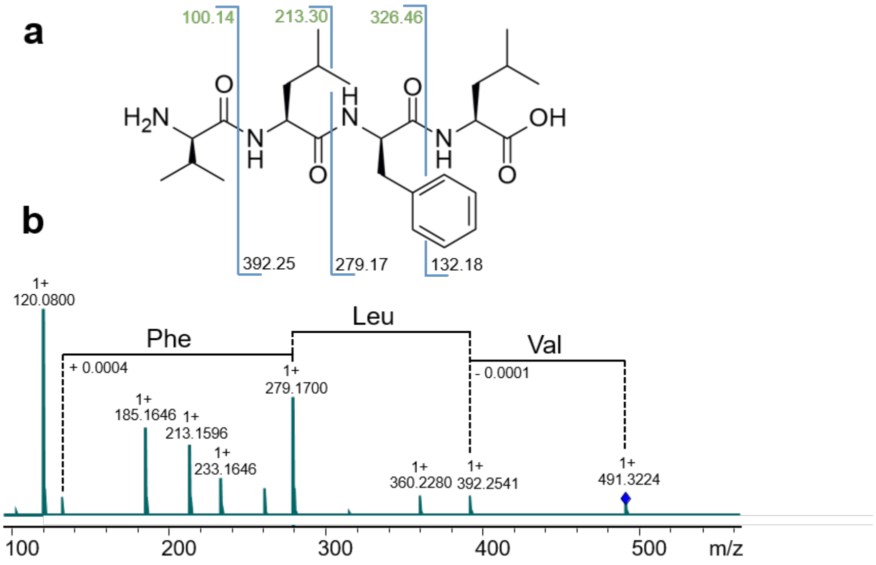

Figure S9. Fragmentation pattern of synthetic 17. (a) Structure of **17** with indicated fragment ions. (b) Fragmentation pattern of **17.**

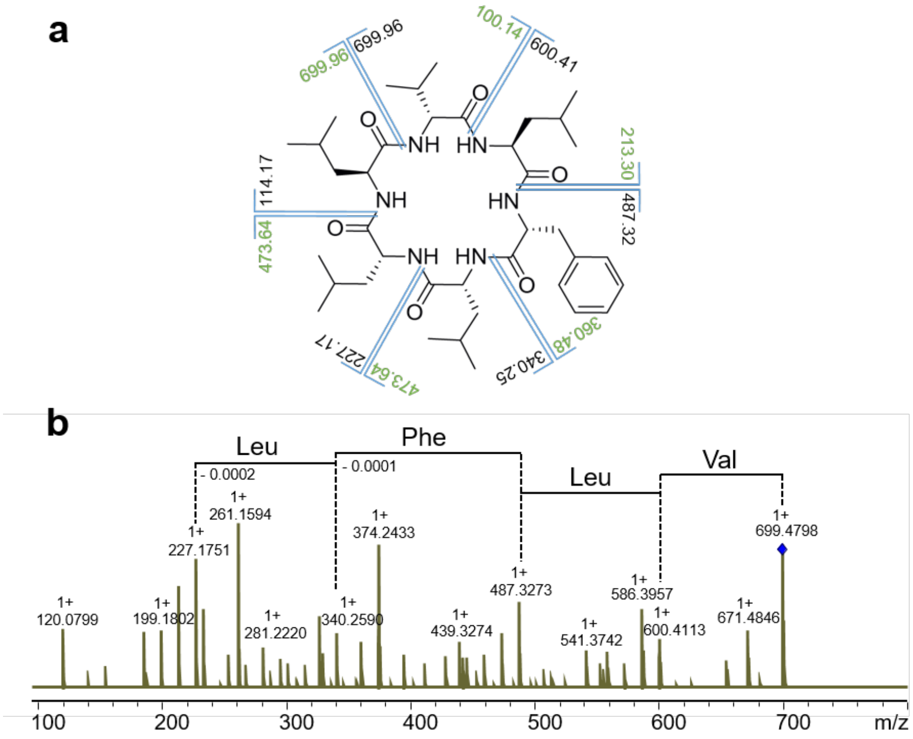

Figure S10. Fragmentation pattern of synthetic 22. (a) Structure of **22** with indicated fragment ions. (b) Fragmentation pattern of **22.**

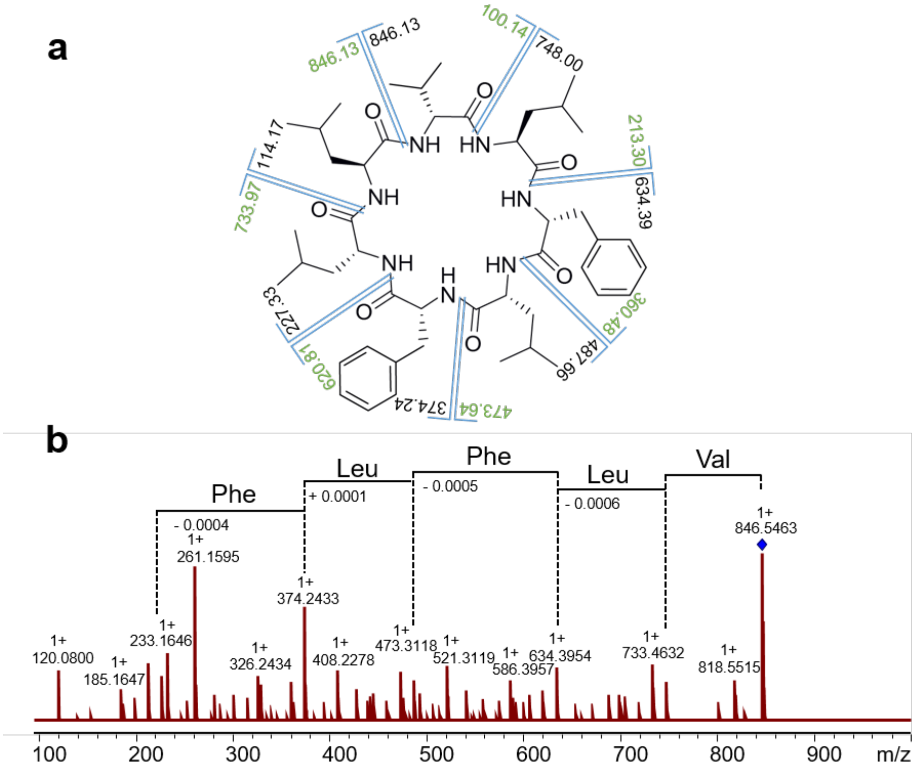

Figure S11. Fragmentation pattern of synthetic 26. (a) Structure of **26** with indicated fragment ions. (b) Fragmentation pattern of **26.**

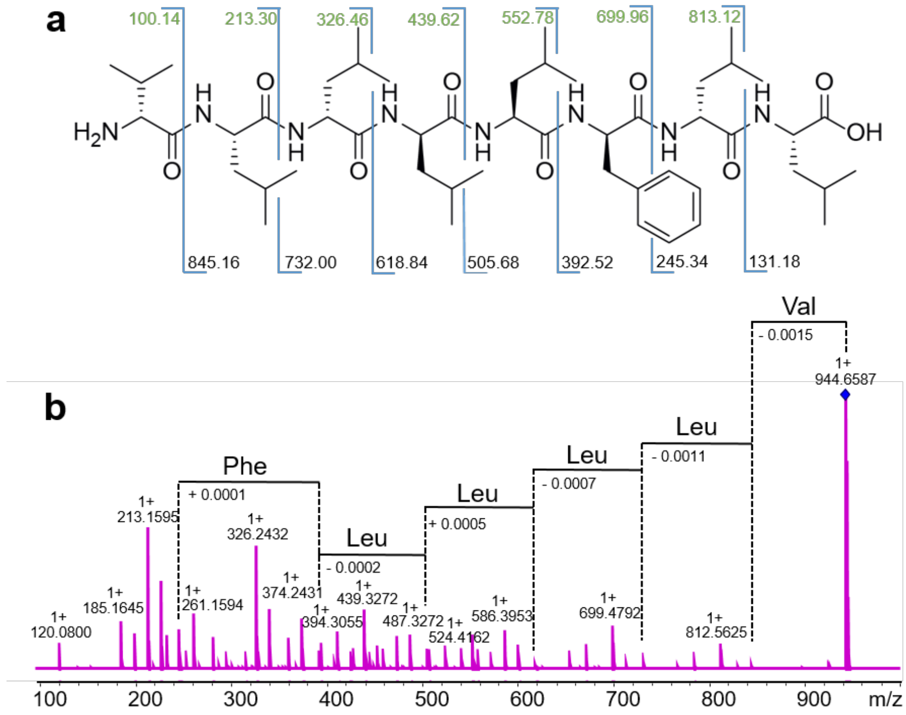

Figure S12. Fragmentation pattern of synthetic 32. (a) Structure of **32** with indicated fragment ions. (b) Fragmentation pattern of **26.**

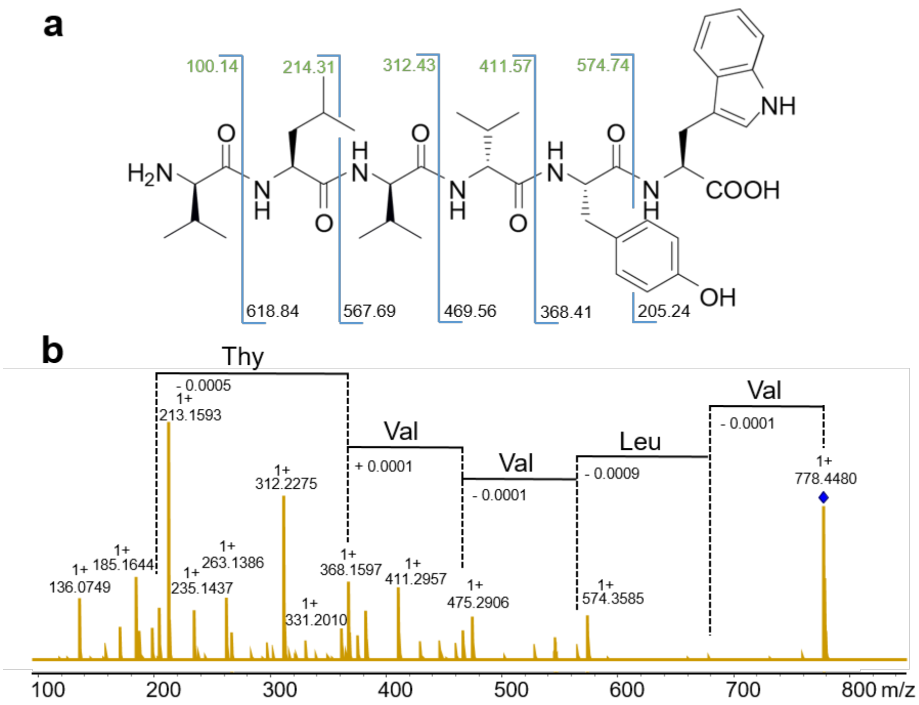

Figure S13. Fragmentation pattern of synthetic 37. (a) Structure of **37** with indicated fragment ions. (b) Fragmentation pattern of **37.**

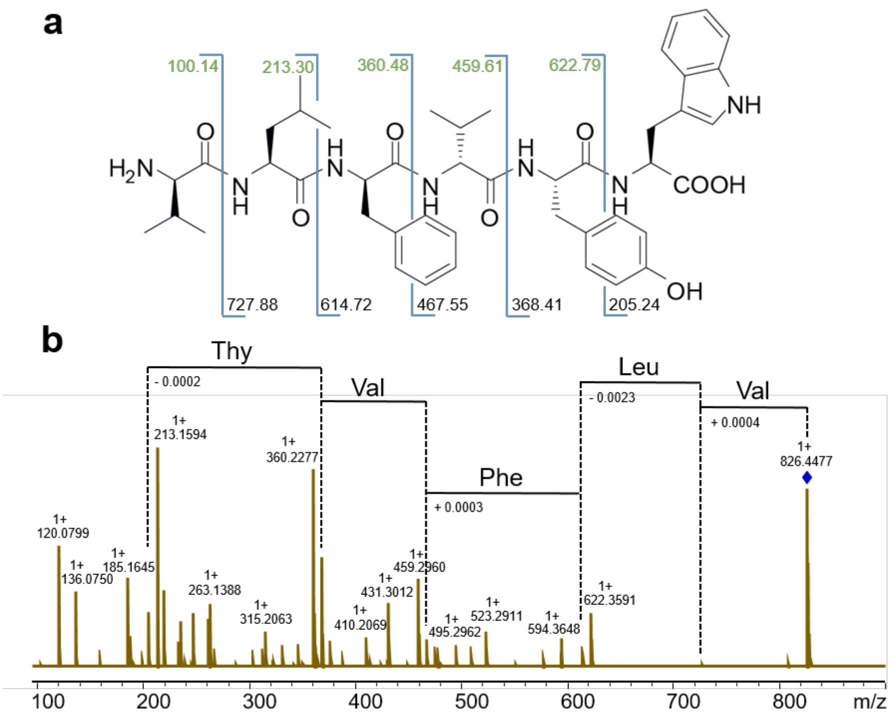

Figure S14. Fragmentation pattern of synthetic 41. (a) Structure of **41** with indicated fragment ions. (b) Fragmentation pattern of **41.**

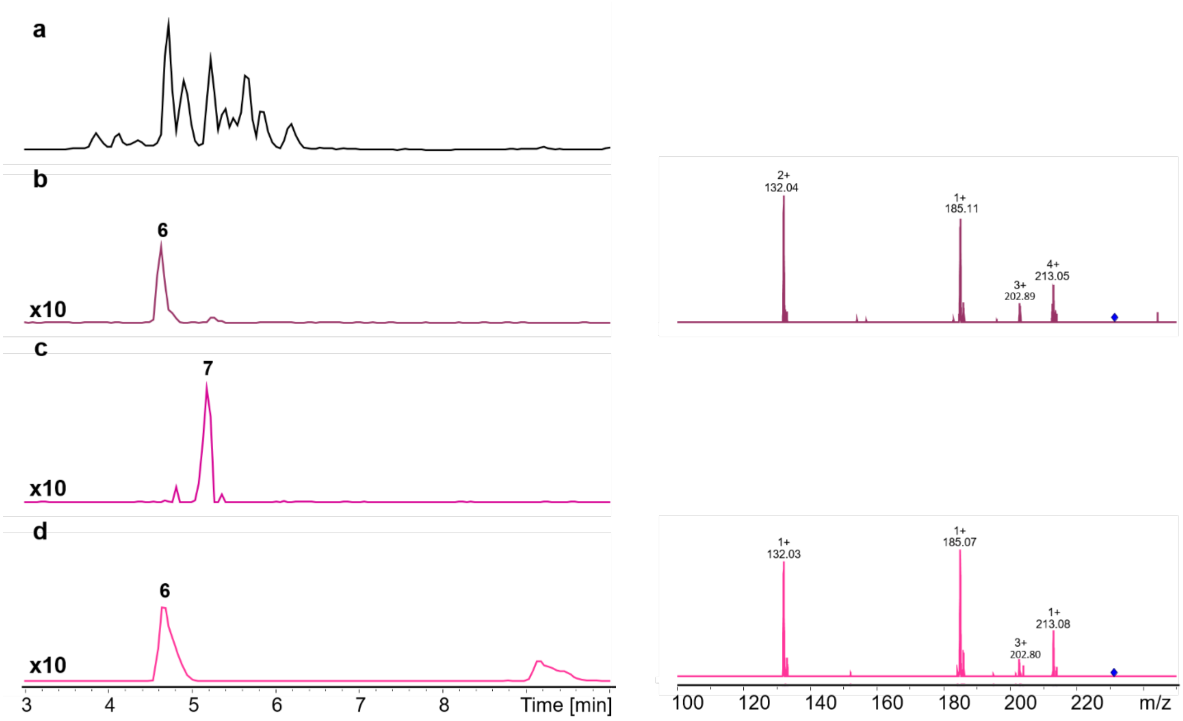

Figure S15. HPLC/MS data (Figure 1b) of compounds 6 and 7 produced in *E. coli* DH10B::*mtaA*. (a) BPC of an exemplary culture extract (b) EIC/MS^2 of **6** (*m/z* [M+H]$^+$ = 231.17, **NRPS-1**). (c) EIC of **7** (*m/z* [M+H]$^+$ = 245.19; **NRPS-1**) (d) EIC/MS^2 data of synthetic **6** (*m/z* [M+H]$^+$ = 231.17).

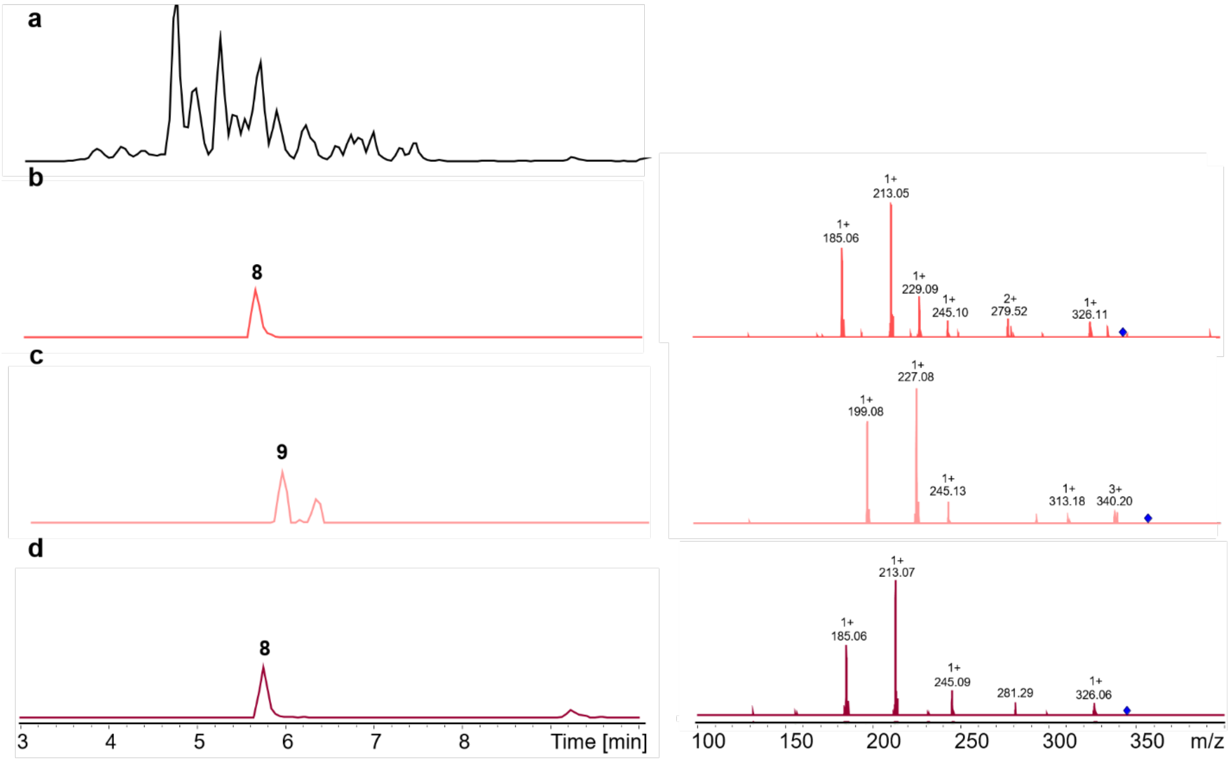

Figure S16. HPLC/MS data (Figure 1b) of compounds 8 and 9 produced in *E. coli* DH10B::*mtaA*. (a) BPC of an exemplary culture extract. (b) EIC/MS^2 of **8** (*m/z* [M+H]$^+$ = 344.25, **NRPS-2**). (c) EIC/MS^2 of **9** (*m/z* [M+H]$^+$ = 358.28; **NRPS-2**). (d) EIC/MS^2 data of synthetic **8** (*m/z* [M+H]$^+$ = 344.25).

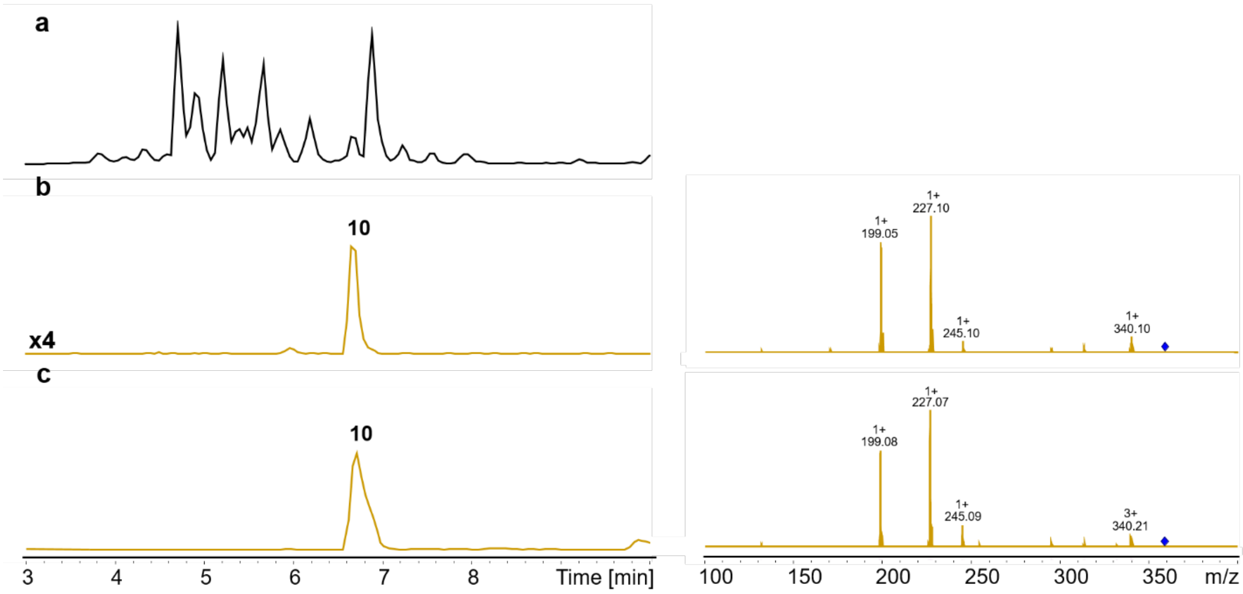

Figure S17. HPLC/MS data (Figure 1b) of compound 10 produced in *E. coli* DH10B::*mtaA*. (a) BPC of an exemplary culture extract. (b) EIC/MS^2 of **10** (*m/z* $[M+H]^+$ = 358.28, **NRPS-3**). (c) EIC/MS^2 data of synthetic **10** (*m/z* $[M+H]^+$ = 358.25).

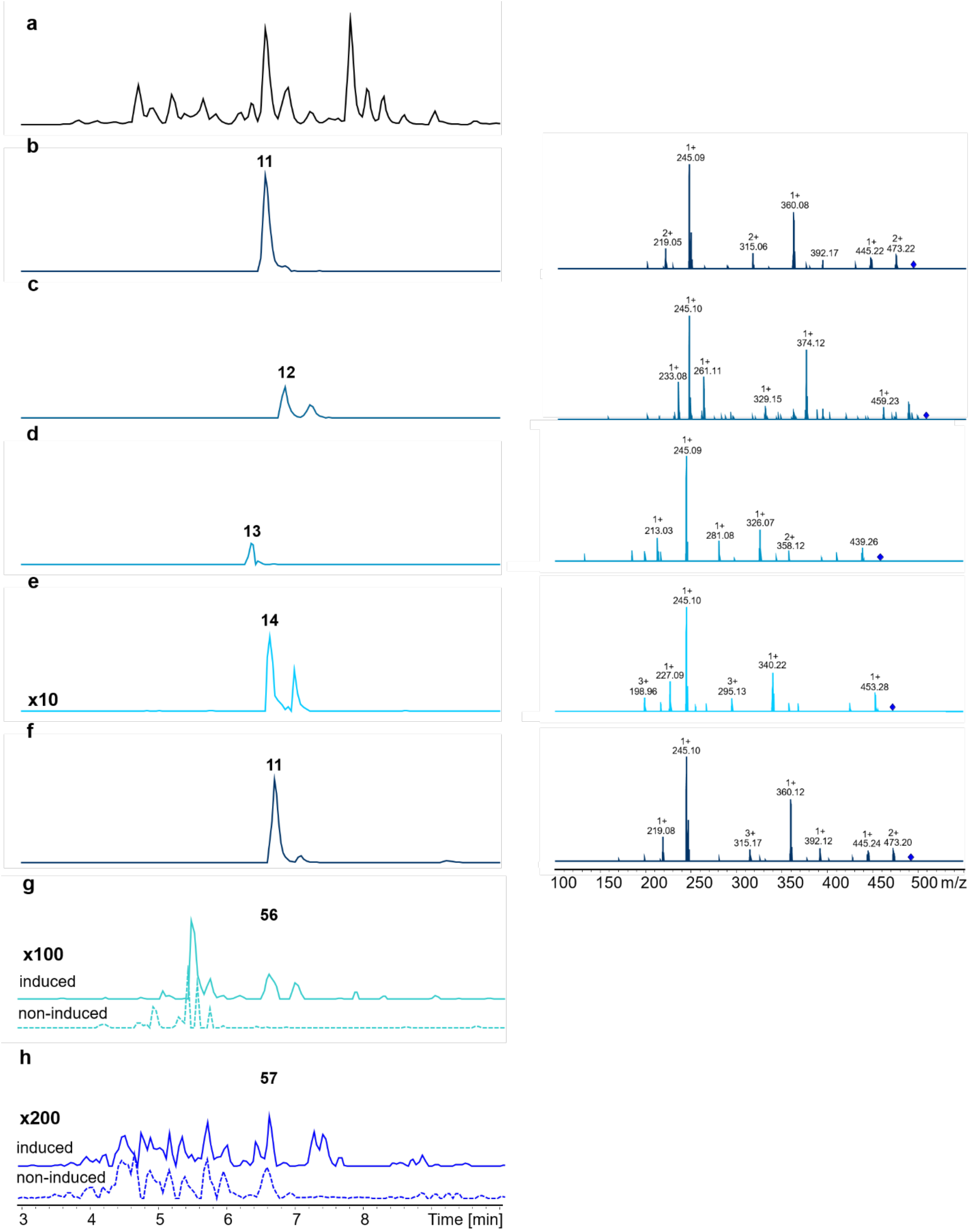
a
b
11
c
12
d
13
e
14
x10
f
11
g
56
x100
induced
non-induced
h
57
x200
induced
non-induced
3
4
5
6
7
8
Time [min]
1+ 245.09
1+ 360.08
2+ 219.05
2+ 315.06
392.17
1+ 445.22
2+ 473.22
1+ 245.10
1+ 374.12
1+ 233.08
1+ 261.11
1+ 329.15
1+ 459.23
1+ 245.09
1+ 213.03
1+ 281.08
1+ 326.07
2+ 358.12
439.26
1+ 245.10
1+ 227.09
3+ 198.96
3+ 295.13
1+ 340.22
1+ 453.28
1+ 245.10
1+ 219.08
3+ 315.17
1+ 360.12
1+ 392.12
1+ 445.24
2+ 473.20
100
150
200
250
300
350
400
450
500 m/z

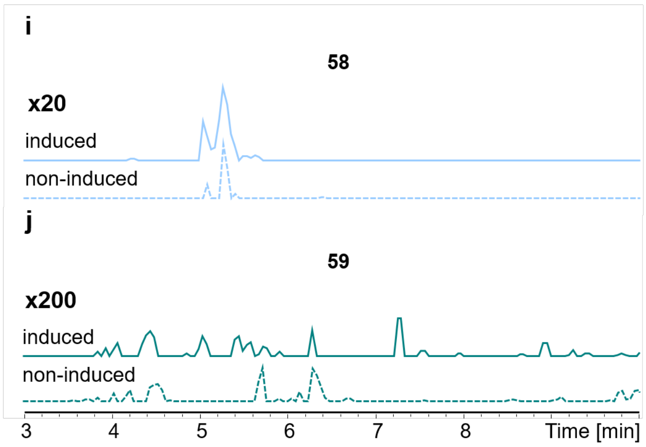

Figure S18. HPLC/MS data (Figure 1b) of compounds 11, 12, 13 and 14 produced in *E. coli* DH10B::*mtaA*. (a) BPC of an exemplary culture extract (b) EIC/MS^2 of **11** (*m/z* $[M+H]^+$ = 491.32, **NRPS-4**). (c) EIC/MS^2 data of **12** (*m/z* $[M+H]^+$ = 505.34, **NRPS-4**). (d) EIC/MS^2 data of **13** (*m/z* $[M+H]^+$ = 457.34, **NRPS-4**). (e) EIC/MS^2 data of **14** (*m/z* $[M+H]^+$ = 471.35, **NRPS-4**). (f) EIC/MS^2 data of synthetic **11** (*m/z* $[M+H]^+$ = 491.32). (g) EIC data of non-produced **56** (*m/z* $[M+H]^+$ = 473.32). (h) EIC data of non-produced **57** (*m/z* $[M+H]^+$ =487.32). (h) EIC data of non-produced **58** (*m/z* $[M+H]^+$ =439.33). (i) EIC data of non-produced **59** (*m/z* $[M+H]^+$ =453.34).

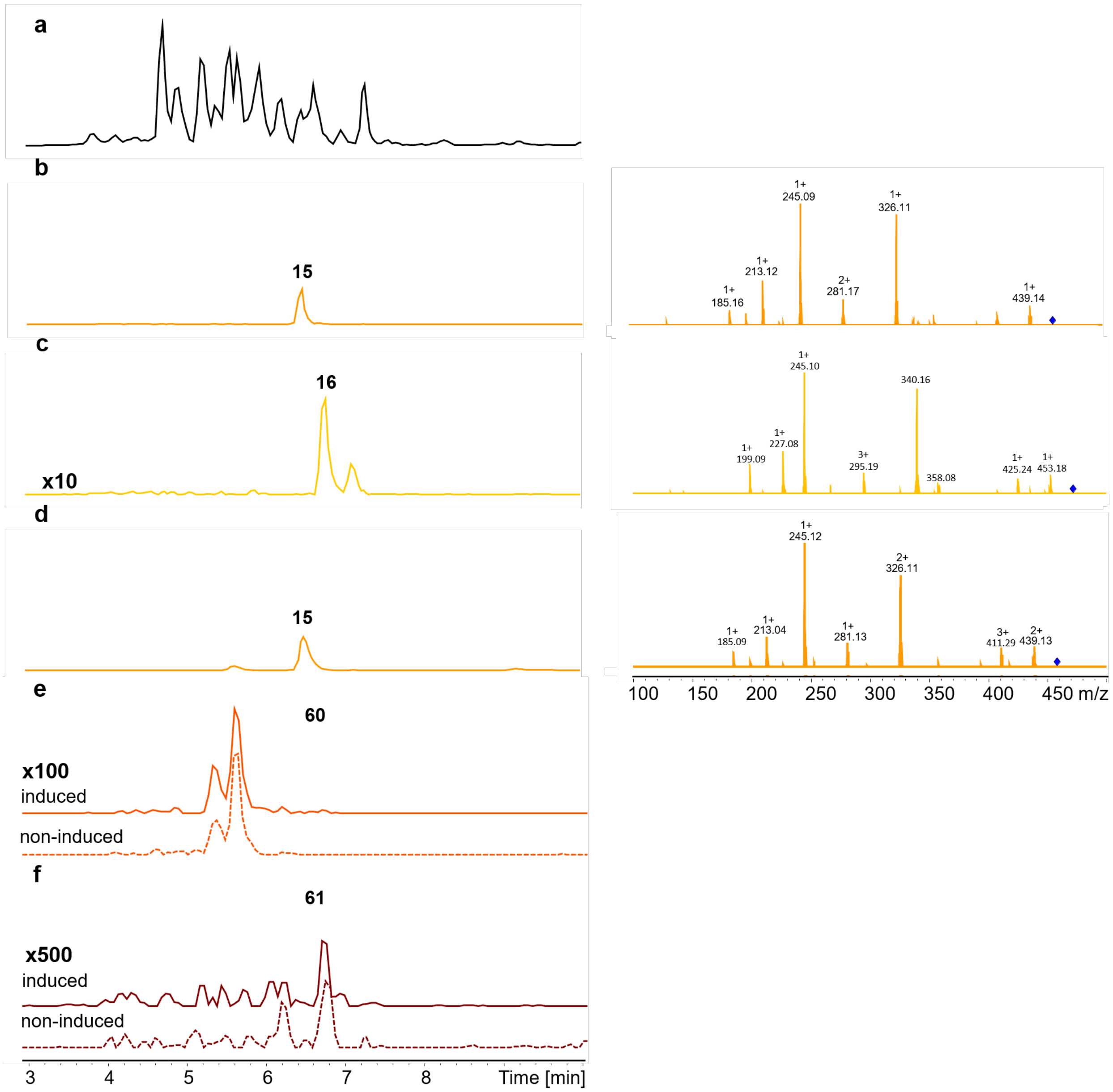

Figure S19. HPLC/MS data (Figure 1b) of compounds 15 and 16 produced in *E. coli* DH10B::*mtaA*. (a) BPC of an exemplary culture extract (b) EIC/MS^2 of **15** (*m/z* [M+H]$^+$ = 457.34, **NRPS-5**). (c) EIC/MS^2 data of **16** (*m/z* [M+H]$^+$ = 471.35, **NRPS-5**). (d) EIC/MS^2 data of synthetic **15** (*m/z* [M+H]$^+$ = 457.34). (e) EIC data of non-produced **60** (*m/z* [M+H]$^+$ =439.33). (f) EIC data of non-produced **61** (*m/z* [M+H]$^+$ = 453.32)

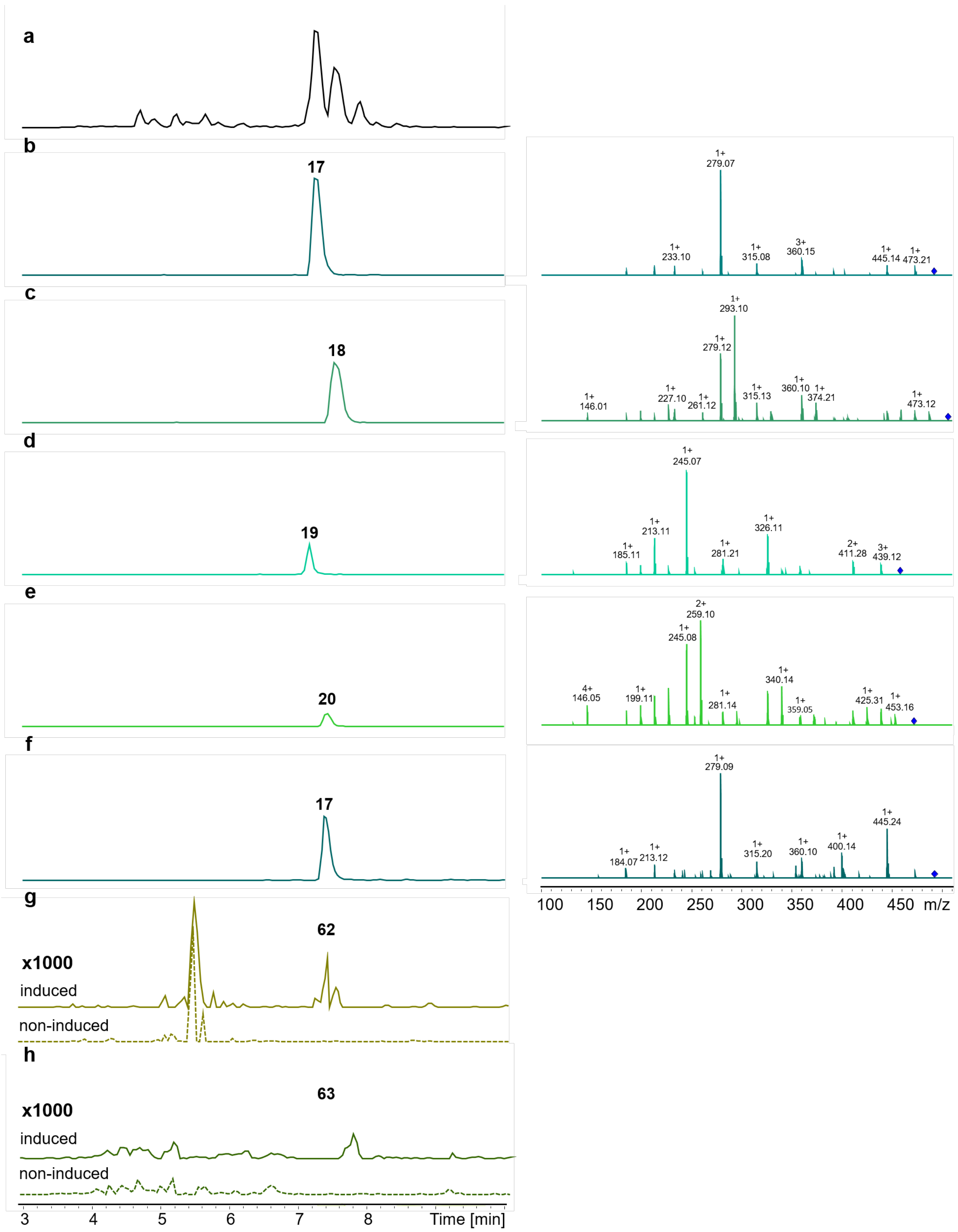
a
b
17
c
18
d
19
e
20
f
17
g
62
x1000
induced
non-induced
h
63
x1000
induced
non-induced
3
4
5
6
7
8
Time [min]
1+ 279.07
1+ 233.10
1+ 315.08
3+ 360.15
1+ 445.14
1+ 473.21
1+ 293.10
1+ 279.12
1+ 146.01
1+ 227.10
1+ 261.12
1+ 315.13
1+ 360.10
1+ 374.21
1+ 473.12
1+ 245.07
1+ 213.11
1+ 185.11
1+ 281.21
1+ 326.11
2+ 411.28
3+ 439.12
2+ 259.10
1+ 245.08
4+ 146.05
1+ 199.11
1+ 281.14
1+ 340.14
1+ 359.05
1+ 425.31
1+ 453.16
1+ 279.09
1+ 445.24
1+ 184.07
1+ 213.12
1+ 315.20
1+ 360.10
1+ 400.14
100
150
200
250
300
350
400
450
m/z

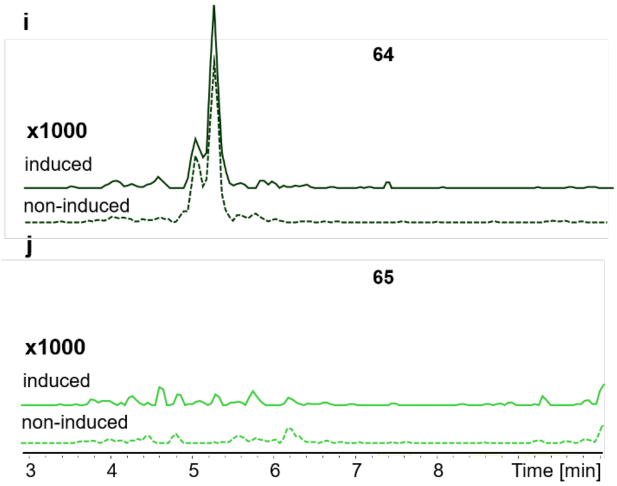

Figure S20. HPLC/MS data (Figure 1b) of compounds 17, 18, 19 and 20 produced in *E. coli* DH10B::*mtaA*. (a) BPC of an exemplary culture extract (b) EIC/MS^2 of **17** (*m/z* $[M+H]^+$ = 491.32, **NRPS-6**). (c) EIC/MS^2 data of **18** (*m/z* $[M+H]^+$ = 505.34, **NRPS-6**). (d) EIC/MS^2 data of **19** (*m/z* $[M+H]^+$ = 457.34, **NRPS-6**). (e) EIC/MS^2 data of **20** (*m/z* $[M+H]^+$ = 471.35, **NRPS-6**). (f) EIC/MS^2 of synthetic **17** (*m/z* $[M+H]^+$ = 491.32). (g) EIC data of **62** (*m/z* $[M+H]^+$ = 473.32). (h) EIC data of **63** (*m/z* $[M+H]^+$ = 487.32). (i) EIC data of **64** (*m/z* $[M+H]^+$ = 439.33). (j) EIC data of **65** (*m/z* $[M+H]^+$ = 453.34).

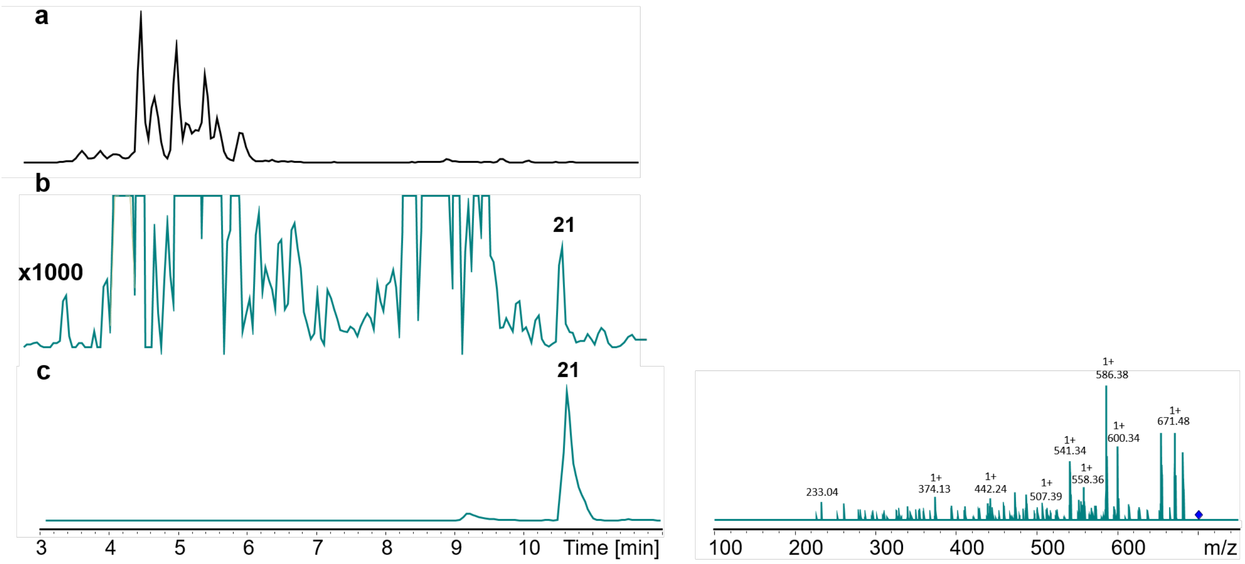

Figure S21. HPLC/MS data (Figure 1c) of compound 21 produced in *E. coli* DH10B::*mtaA*. (a) BPC of an exemplary culture extract (b) EIC of **21** (*m/z* $[M+H]^+$ = 699.48, **NRPS-7**). (c) EIC/MS^2 of synthetic **21** (*m/z* $[M+H]^+$ = 699.48).

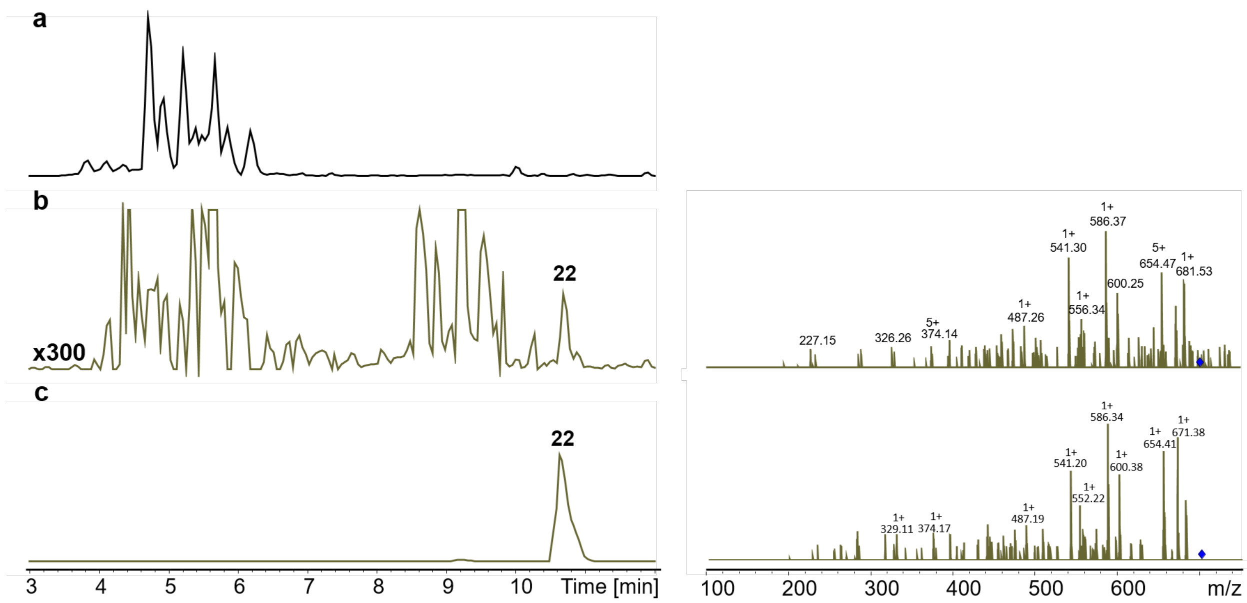

Figure S22. HPLC/MS data (Figure 1c) of compound 22 produced in *E. coli* DH10B::*mtaA*. (a) BPC of an exemplary culture extract (b) EIC/MS^2 of **22** (*m/z* $[M+H]^+$ = 699.48, **NRPS-8**). (c) EIC/MS^2 of synthetic **22** (*m/z* $[M+H]^+$ = 699.48).

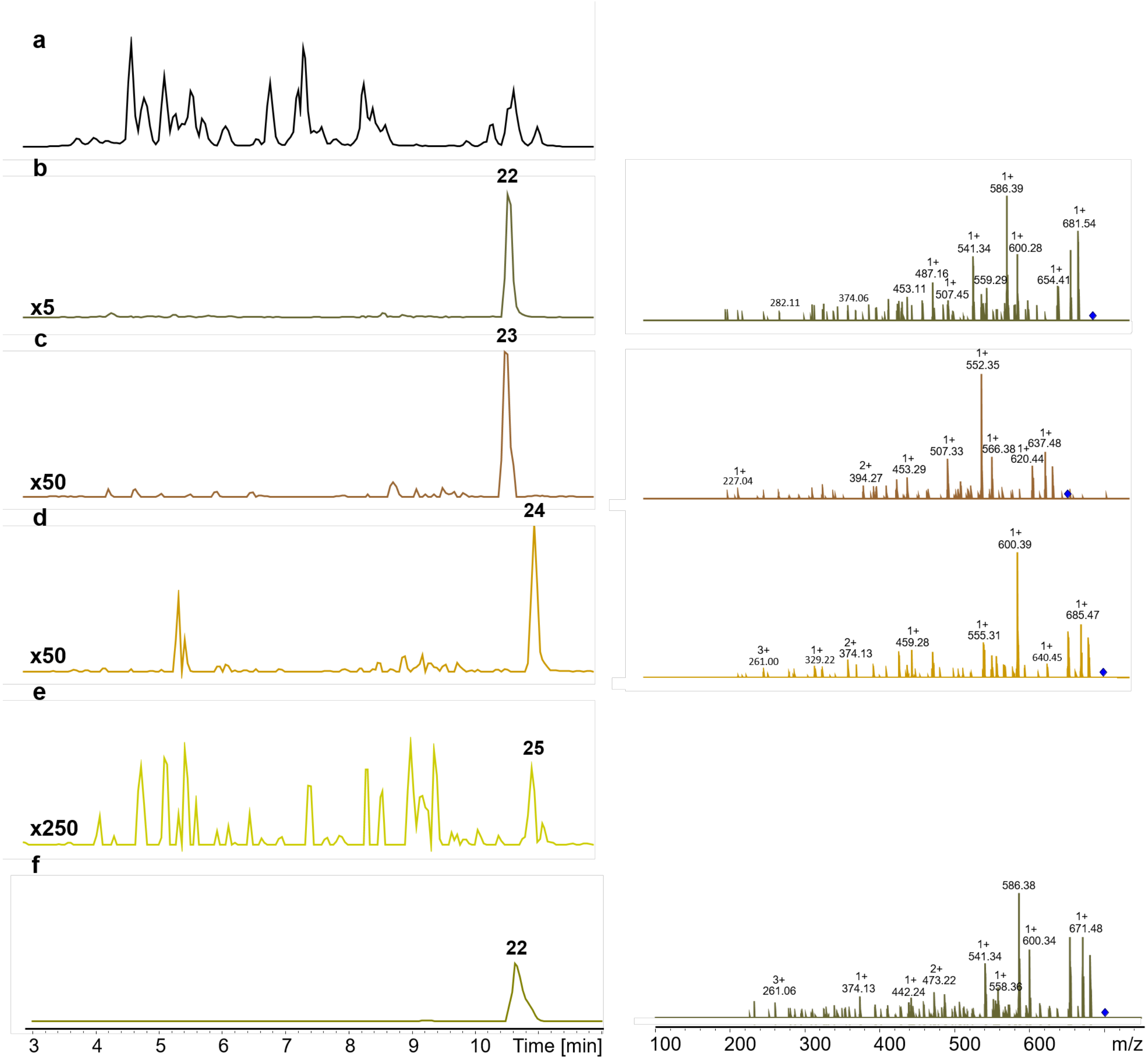

Figure S23. HPLC/MS data (Figure 1c) of compounds 22, 23, 24 and 25 produced in *E. coli* DH10B::*mtaA*. (a) BPC of an exemplary culture extract (b) EIC/MS^2 of **22** (*m/z* $[M+H]^+$ = 699.48, **NRPS-9**). (c) EIC/MS^2 data of **23** (*m/z* $[M+H]^+$ = 713.50, **NRPS-9**). (d) EIC/MS^2 data of **24** (*m/z* $[M+H]^+$ = 665.50, **NRPS-9**). (e) EIC data of **25** (*m/z* $[M+H]^+$ = 679.51). (f) EIC/MS^2 of synthetic **22** (*m/z* $[M+H]^+$ = 491.32).

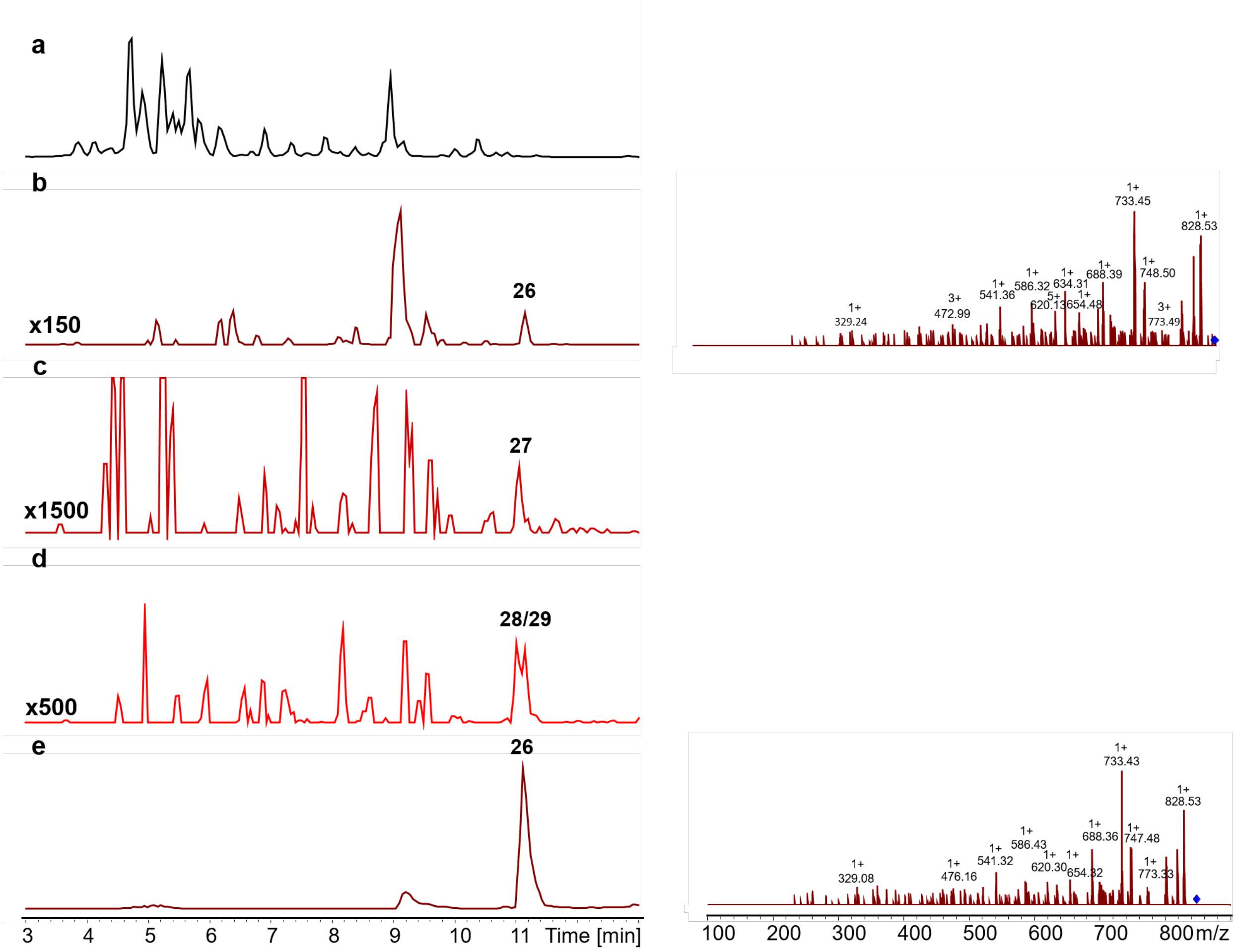

Figure S24. HPLC/MS data (Figure 1c) of compounds 26, 27 and 28/29 produced in *E. coli* DH10B::*mtaA*. (a) BPC of an exemplary culture extract (b) EIC/MS^2 of **26** (*m/z* $[M+H]^+$ = 846.60, **NRPS-11**). (c) EIC data of **27** (*m/z* $[M+H]^+$ = 778.58, **NRPS-11**). (d) EIC data of **28/29** (*m/z* $[M+H]^+$ = 812.56, **NRPS-11**). (e) EIC/MS^2 of synthetic **26** (*m/z* $[M+H]^+$ = 846.60).

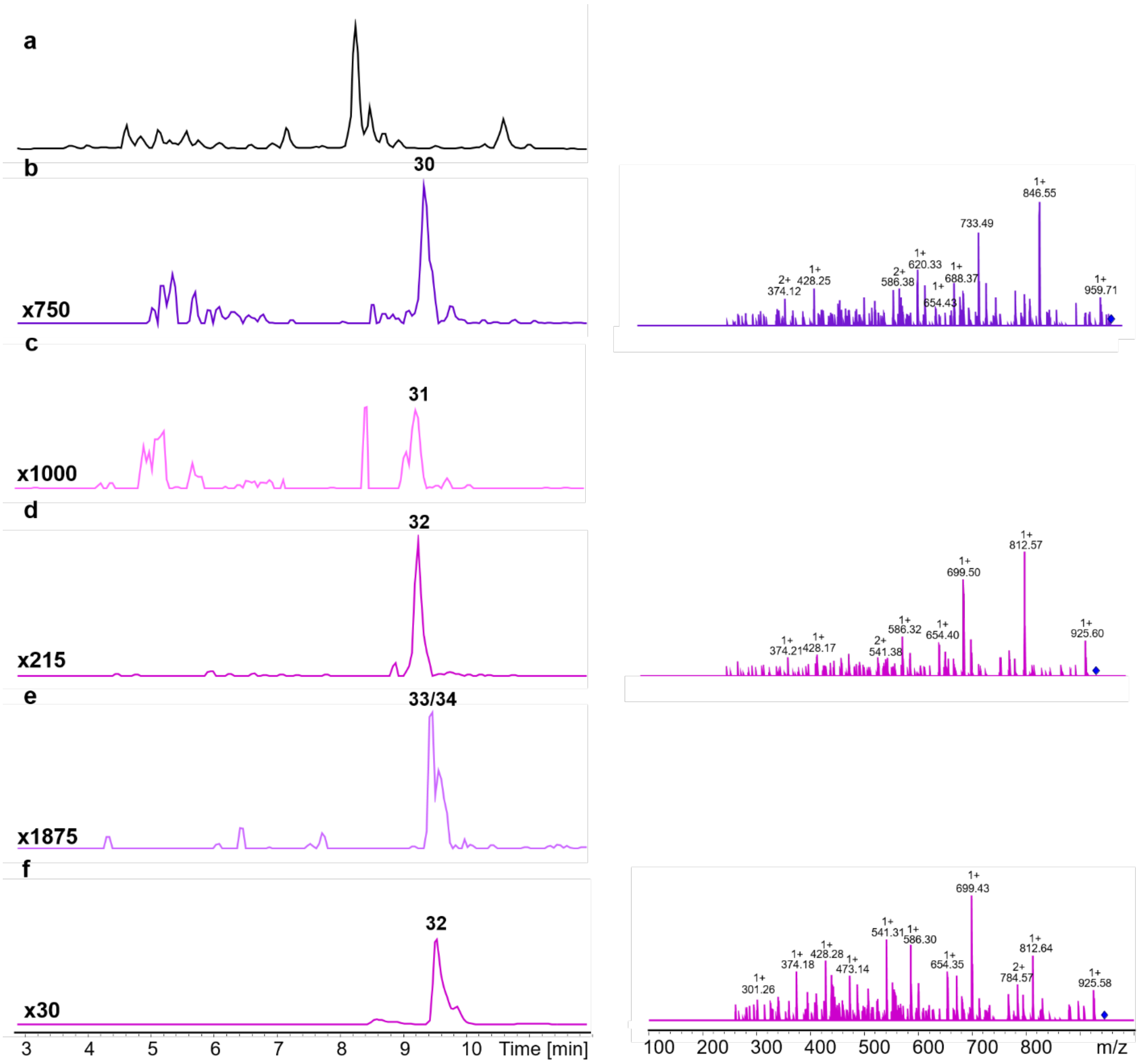

Figure S25. HPLC/MS data (Figure 1c) of compounds 30, 31, 32 and 33/34 produced in *E. coli* DH10B::*mtaA*. (a) BPC of an exemplary culture extract (b) EIC/MS^2 of **30** (*m/z* $[M+H]^+$ = 977.64, **NRPS-12**). (c) EIC data of **31** (*m/z* $[M+H]^+$ = 909.67, **NRPS-12**). (d) EIC data of **32** (*m/z* $[M+H]^+$ = 943.66, **NRPS-12**). (e) EIC data of **33/34** (*m/z* $[M+H]^+$ = 957.67, **NRPS-12**). (f) EIC/MS^2 of synthetic **32** (*m/z* $[M+H]^+$ = 943.66).

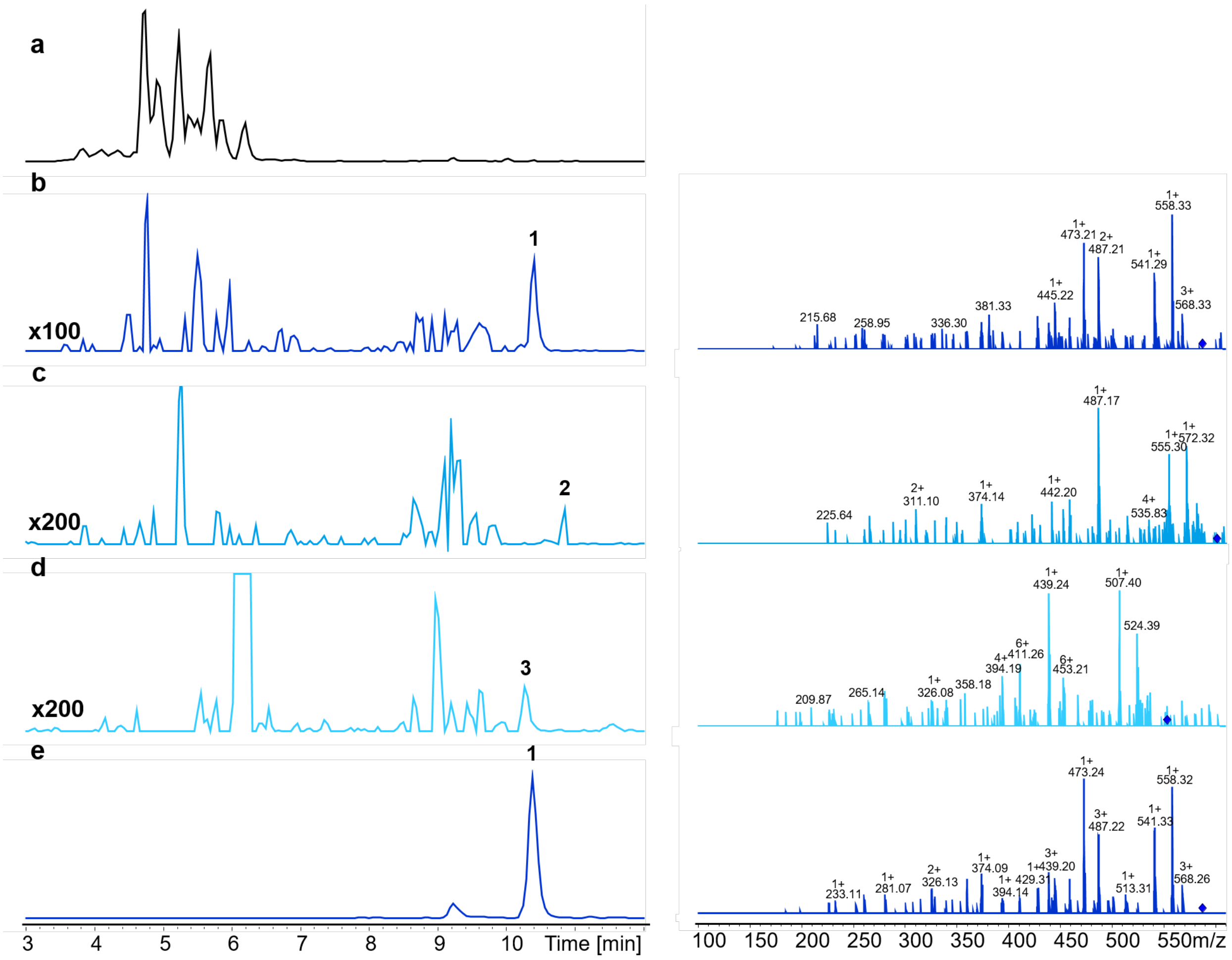

Figure S26. HPLC/MS data (Figure 1d) of compounds 1, 2 and 3 produced in *E. coli* DH10B::*mtaA*. (a) BPC of an exemplary culture extract (b) EIC/MS^2 of **1** (*m/z* $[M+H]^+$ = 586.40, **NRPS-13**). (c) EIC data of **2** (*m/z* $[M+H]^+$ = 600.41, **NRPS-13**). (d) EIC data of **3** (*m/z* $[M+H]^+$ = 552.41, **NRPS-13**). (e) EIC/MS^2 of synthetic **1** (*m/z* $[M+H]^+$ = 586.40).

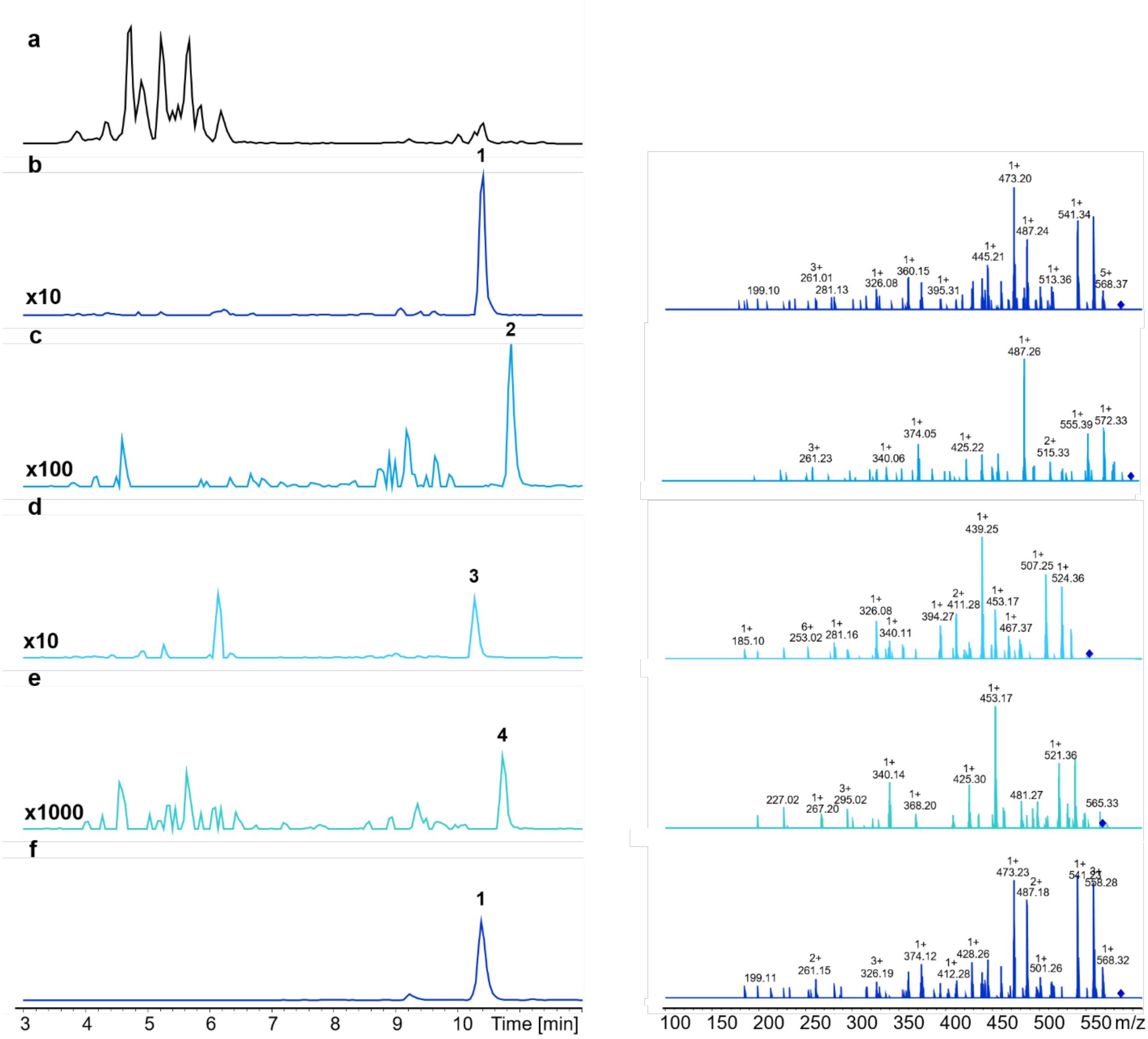

Figure S27. HPLC/MS data (Figure 1d) of compounds 1, 2, 3 and 4 produced in *E. coli* DH10B::*mtaA*. (a) BPC of an exemplary culture extract (b) EIC/MS^2 of **1** (*m/z* $[M+H]^+$ = 586.40, **NRPS-14**). (c) EIC data of **2** (*m/z* $[M+H]^+$ = 600.41, **NRPS-14**). (d) EIC data of **3** (*m/z* $[M+H]^+$ = 552.41, **NRPS-14**). (e) EIC data of **4** (*m/z* $[M+H]^+$ = 566.43, **NRPS-14**). (f) EIC/MS^2 of synthetic **1** (*m/z* $[M+H]^+$ = 586.40).

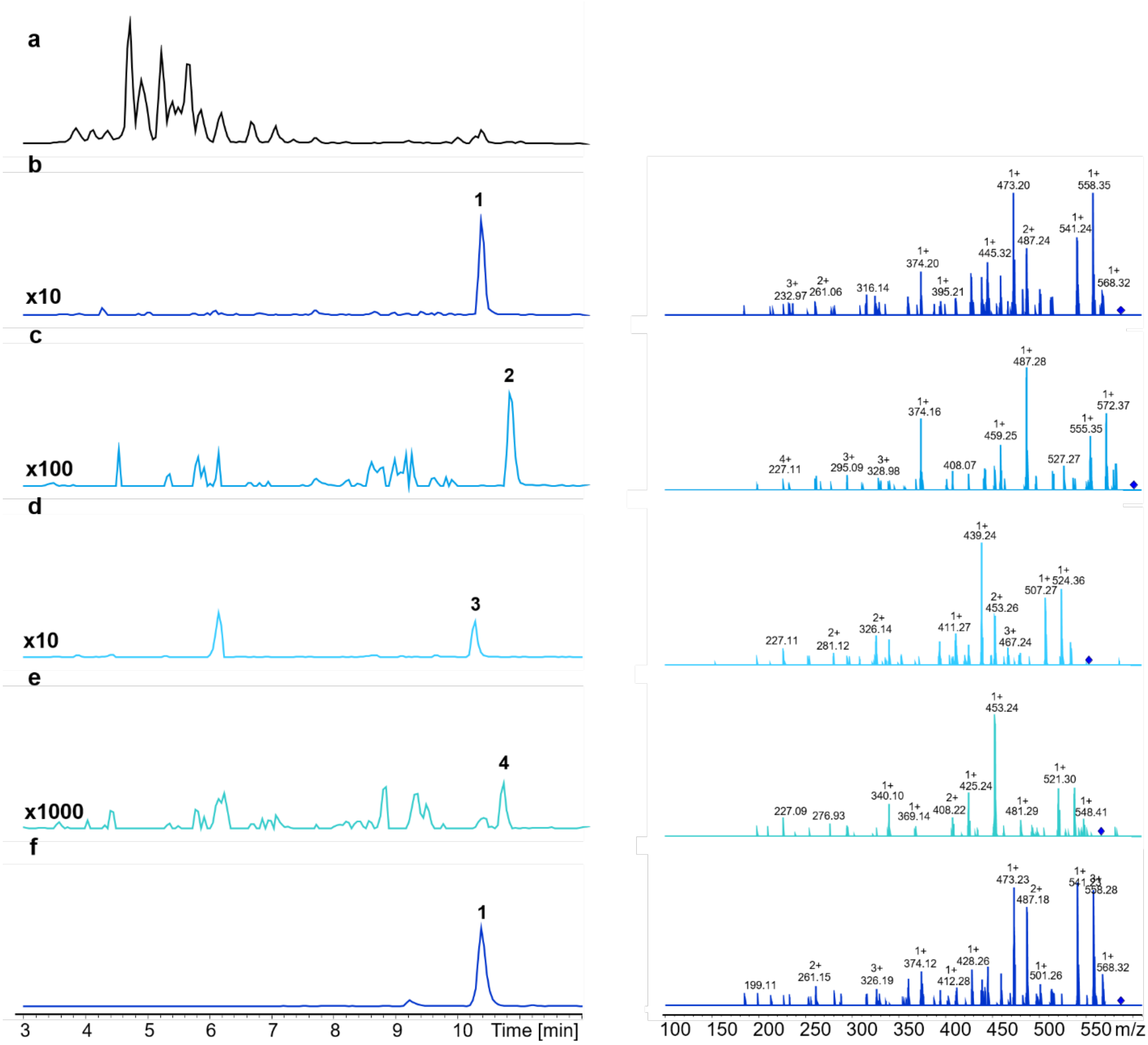

Figure S28. HPLC/MS data (Figure 1d) of compounds 1, 2, 3 and 4 produced in *E. coli* DH10B::*mtaA*. (a) BPC of an exemplary culture extract (b) EIC/MS^2 of **1** (*m/z* [M+H]$^+$ = 586.40, **NRPS-15**). (c) EIC data of **2** (*m/z* [M+H]$^+$ = 600.41, **NRPS-15**). (d) EIC data of **3** (*m/z* [M+H]$^+$ = 552.41, **NRPS-15**). (e) EIC data of **4** (*m/z* [M+H]$^+$ = 566.43, **NRPS-15**). (f) EIC/MS^2 of synthetic **1** (*m/z* [M+H]$^+$ = 586.40).

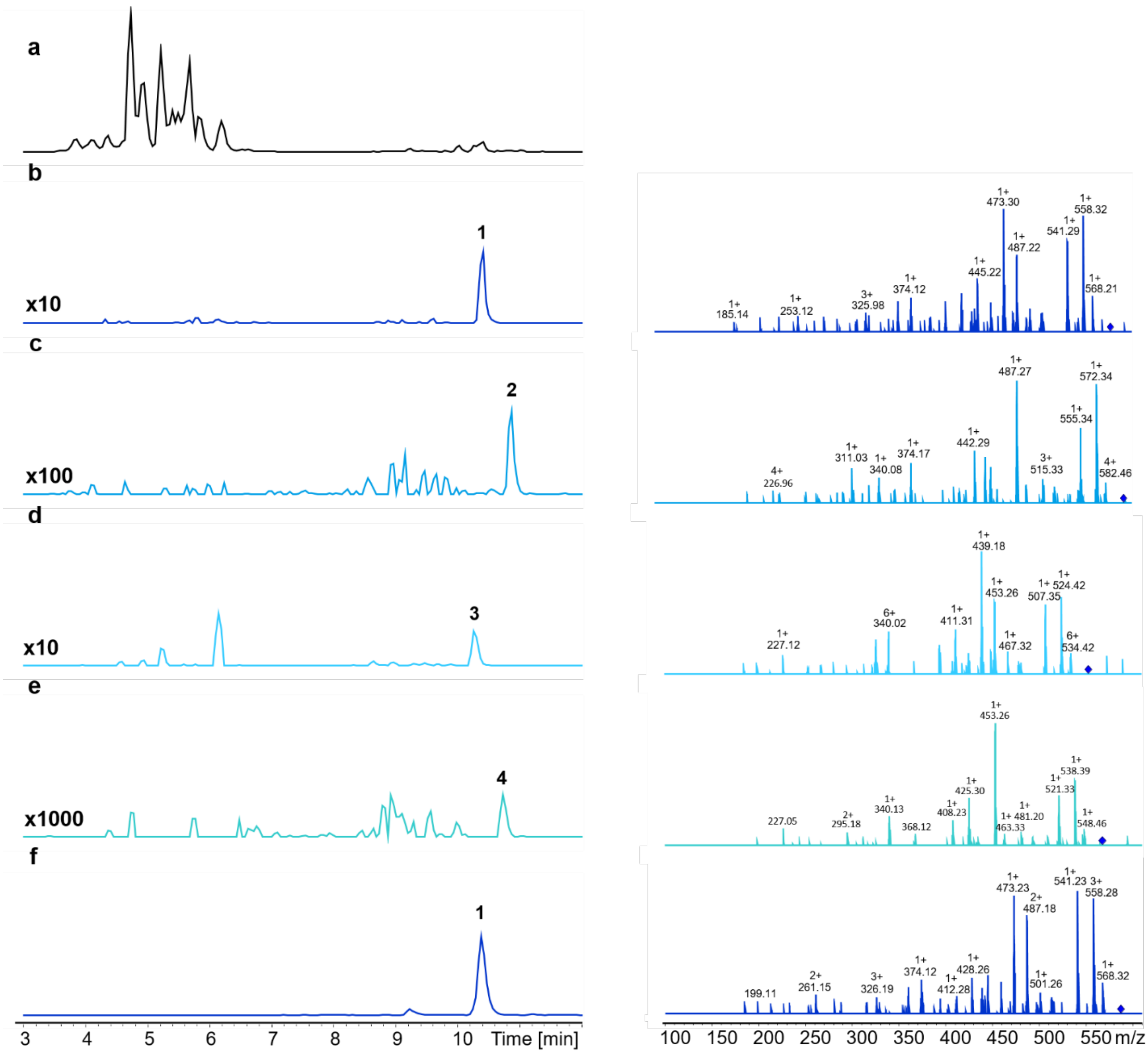

Figure S29. HPLC/MS data (Figure 1d) of compounds 1, 2, 3 and 4 produced in *E. coli* DH10B::*mtaA*. (a) BPC of an exemplary culture extract (b) EIC/MS^2 of **1** (*m/z* [M+H]$^+$ = 586.40, **NRPS-16**). (c) EIC data of **2** (*m/z* [M+H]$^+$ = 600.41, **NRPS-16**). (d) EIC data of **3** (*m/z* [M+H]$^+$ = 552.41, **NRPS-16**). (e) EIC data of **4** (*m/z* [M+H]$^+$ = 566.43, **NRPS-16**). (f) EIC/MS^2 of synthetic **1** (*m/z* [M+H]$^+$ = 586.40).

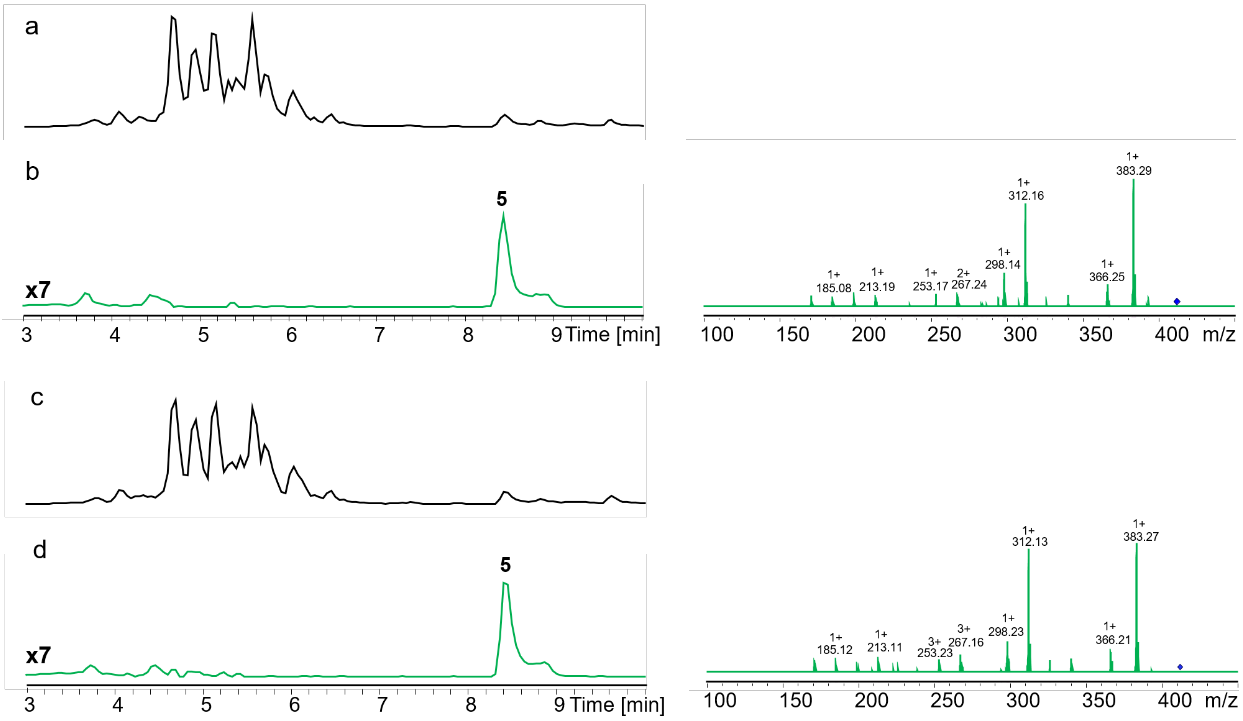

Figure S30. HPLC/MS data refers to Figure 2 (NRPS-18, NRPS-19) of compound 5 produced in *E. coli* DH10B::*mtaA*. (a) BPC of an exemplary culture extract (**NRPS-18**). (b) EIC/MS^2 data of **5** (*m/z* $[M+H]^+$ = 411.30, **NRPS-18**). (c) BPC of an exemplary culture extract (**NRPS-19**). (d) EIC/MS^2 data of **5** (*m/z* $[M+H]^+$ = 411.30, **NRPS-19**).

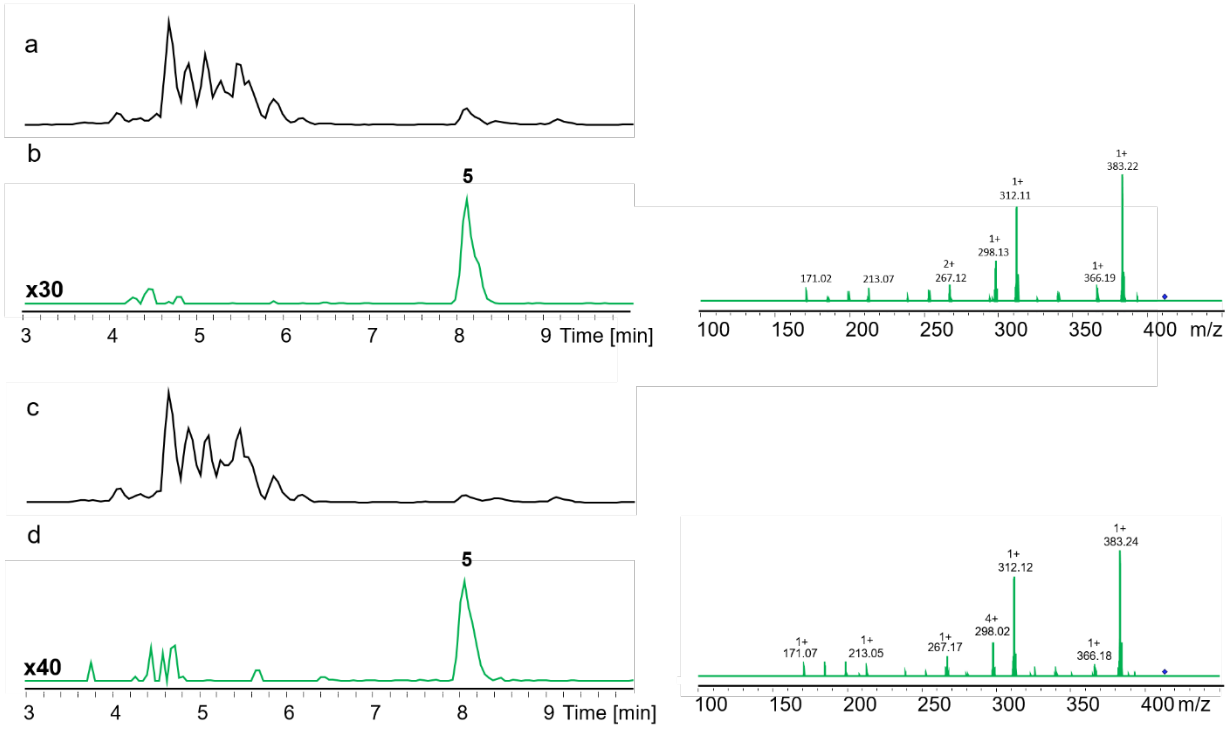

Figure S31. HPLC/MS data refers to Figure 2 (NRPS-20, NRPS-21) of compound 5 produced in *E. coli* DH10B::*mtaA*. (a) BPC of an exemplary culture extract (**NRPS-20**). (b) EIC/MS^2 data of **5** (*m/z* [M+H]$^+$ = 411.30, **NRPS-20**). (c) BPC of an exemplary culture extract (**NRPS-21**). (d) EIC/MS^2 data of **5** (*m/z* [M+H]$^+$ = 411.30, **NRPS-21**).

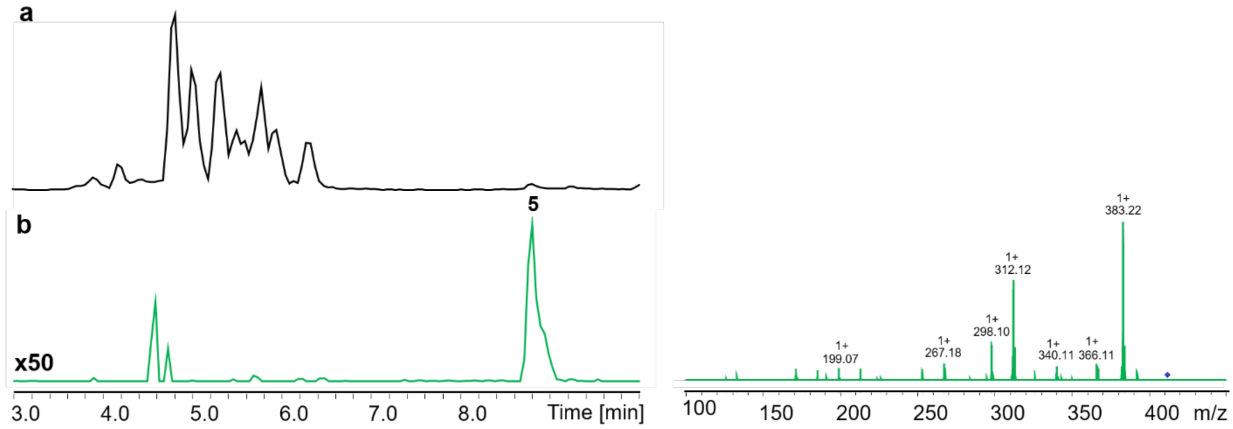

Figure S32. HPLC/MS data refers to Figure 4 (NRPS-22) of compound 5 produced in *E. coli* DH10B::*mtaA*. (a) BPC of an exemplary culture extract. (b) EIC/MS^2 data of **5** (*m/z* [M+H]$^+$ = 411.30).

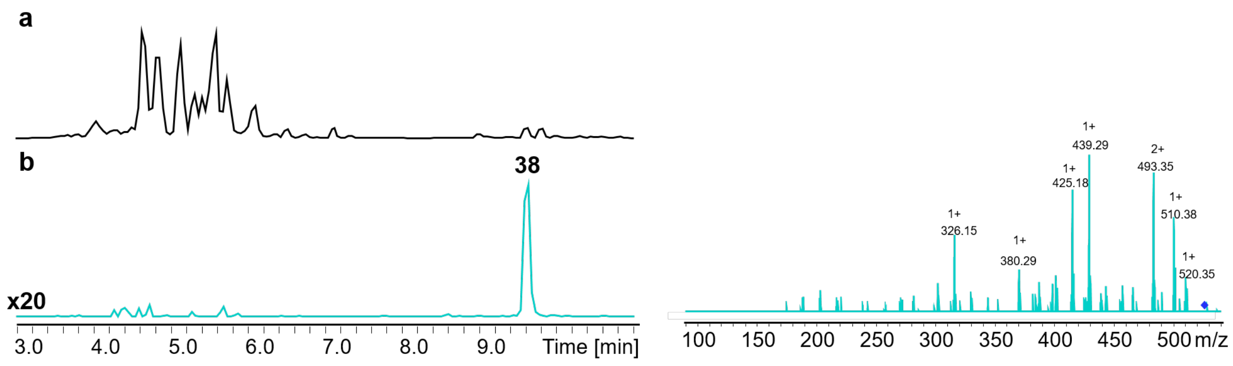

Figure S33. HPLC/MS data refers to Figure 4 (NRPS-23) of compound 38 produced in E. coli DH10B::mtaA. (a) BPC of an exemplary culture extract. (b) EIC/MS^2 data of **38** (m/z $[M+H]^+$ = 538.40).

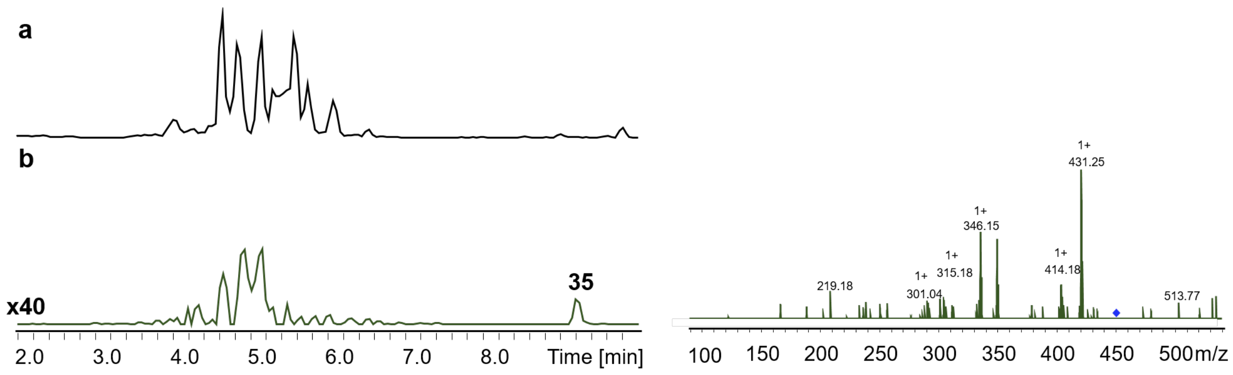

Figure S34. HPLC/MS data refers to Figure 4 (NRPS-24) of compound 35 produced in *E. coli* DH10B::*mtaA*. (a) BPC of an exemplary culture extract. (b) EIC/MS^2 data of **35** (*m/z* $[M+H]^+$ = 459.30).

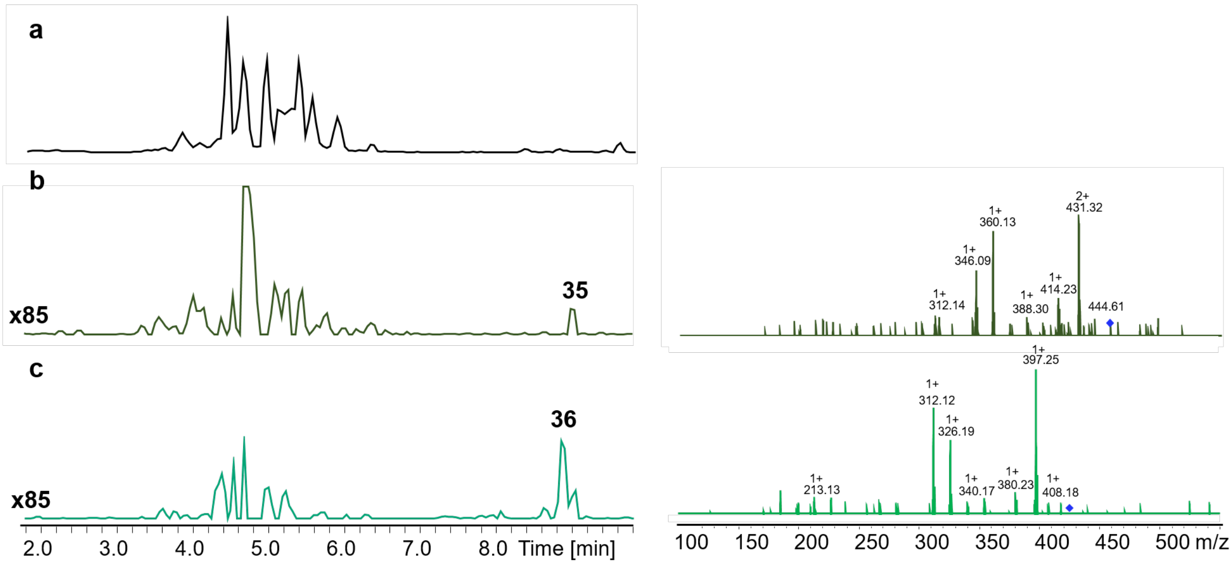

Figure S35. HPLC/MS data refers to Figure 4 (NRPS-25) of compounds 35 and 36 produced in *E. coli* DH10B::*mtaA*. (a) BPC of an exemplary culture extract. (b) EIC/MS^2 data of **35** (m/z [M+H]$^+$ = 459.30). (c) EIC/MS^2 data of **36** (m/z [M+H]$^+$ = 425.31).

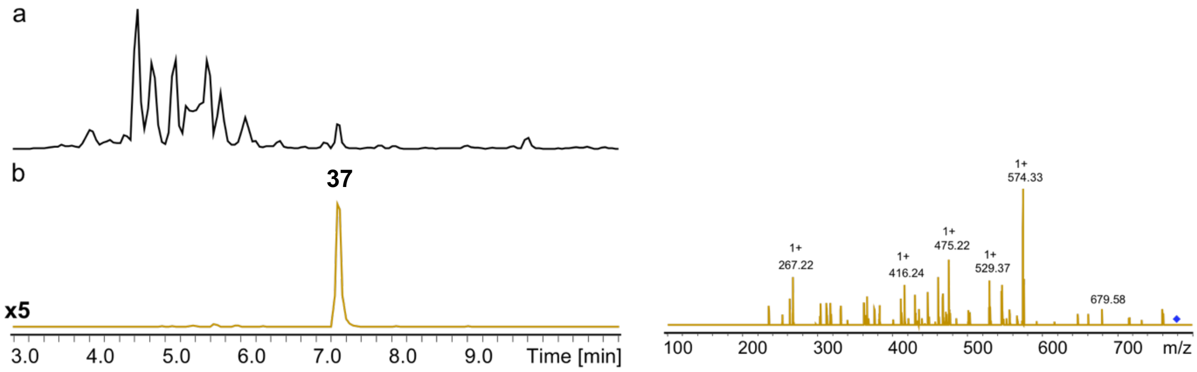

Figure S36. HPLC/MS data refers to Figure 4 (NRPS-26) of compound 37 produced in *E. coli* DH10B::*mtaA*. (a) BPC of an exemplary culture extract. (b) EIC/MS^2 data of **37** (*m/z* [M+H]$^+$ = 778.45).

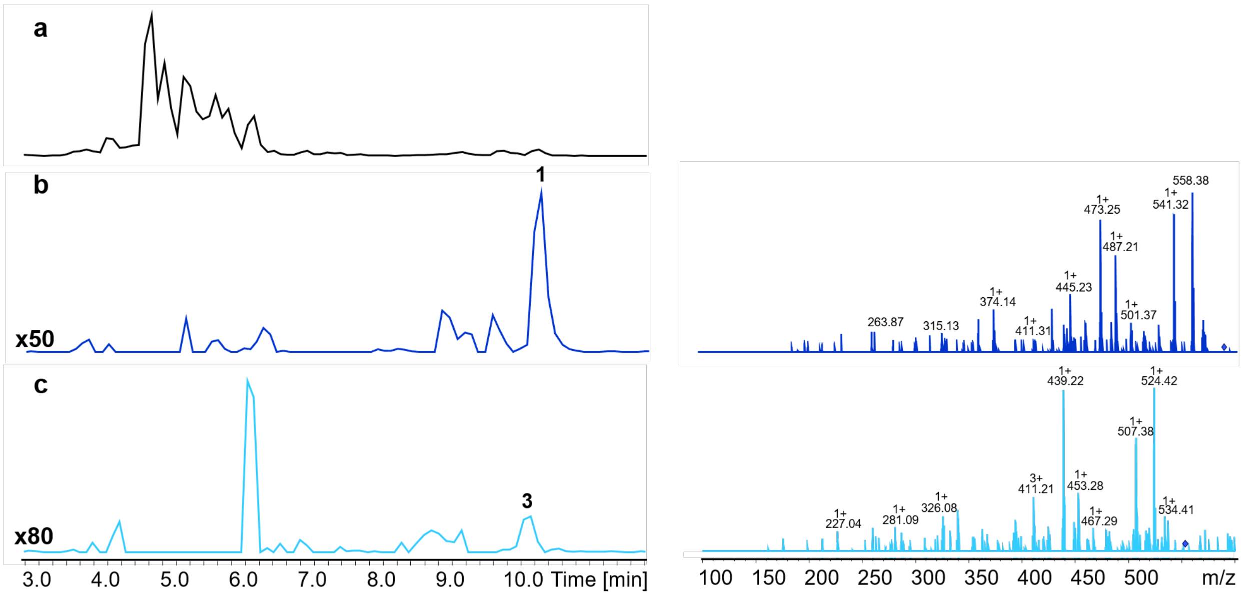

Figure S37. HPLC/MS data refers to Figure 4 (NRPS-27) of compounds 1 and 3 produced in *E. coli* DH10B::*mtaA*. (a) BPC of an exemplary culture extract. (b) EIC/MS^2 data of **1** (m/z $[M+H]^+$ = 586.40). (c) EIC/MS^2 data of **3** (m/z $[M+H]^+$ = 552.41).

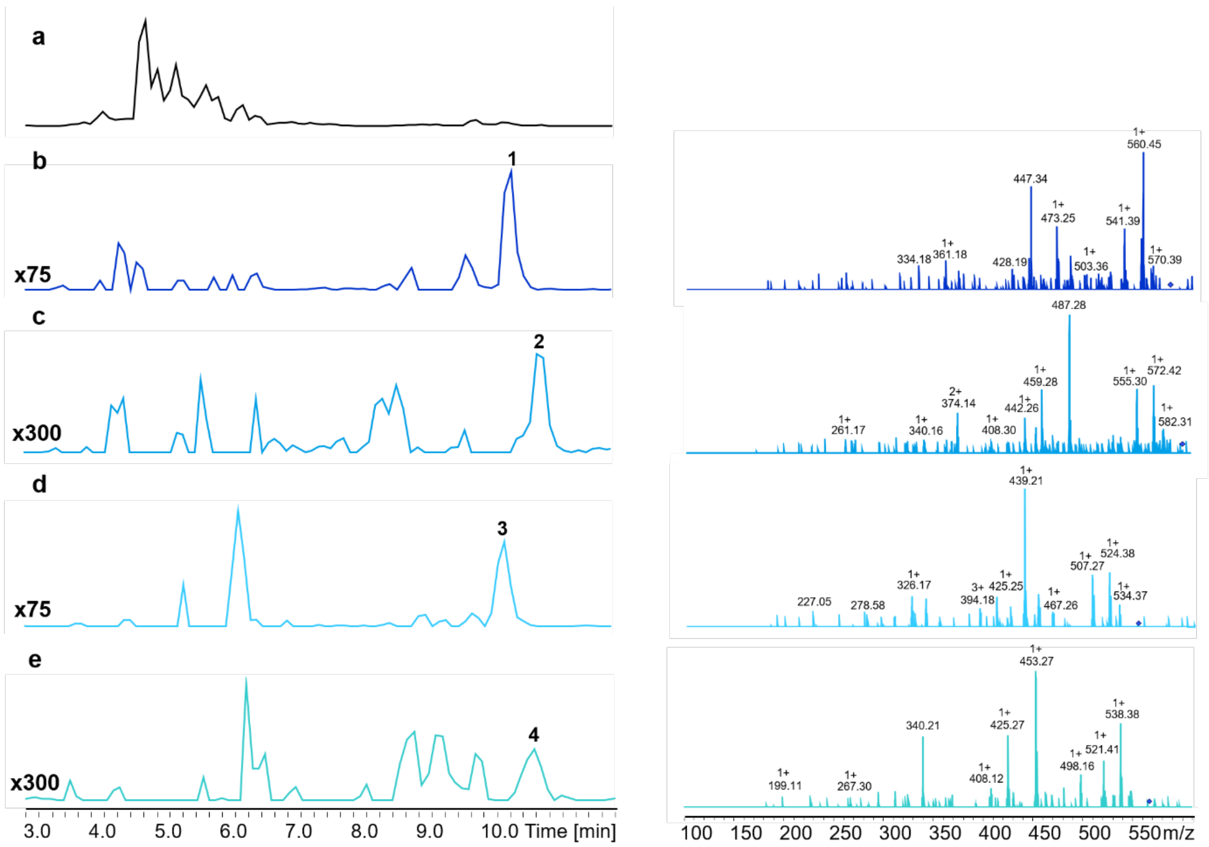

Figure S38. HPLC/MS data refers to Figure 4 (NRPS-28) of compounds 1, 2, 3 and 4 produced in *E. coli* DH10B::*mtaA*. (a) BPC of an exemplary culture extract. (b) EIC/MS^2 data of **1** (*m/z* $[M+H]^+$ = 586.40). (c) EIC/MS^2 data of **2** (*m/z* $[M+H]^+$ = 600.41). (d) EIC/MS^2 data of **3** (*m/z* $[M+H]^+$ = 552.41). (e) EIC/MS^2 data of **4** (*m/z* $[M+H]^+$ = 566.43).

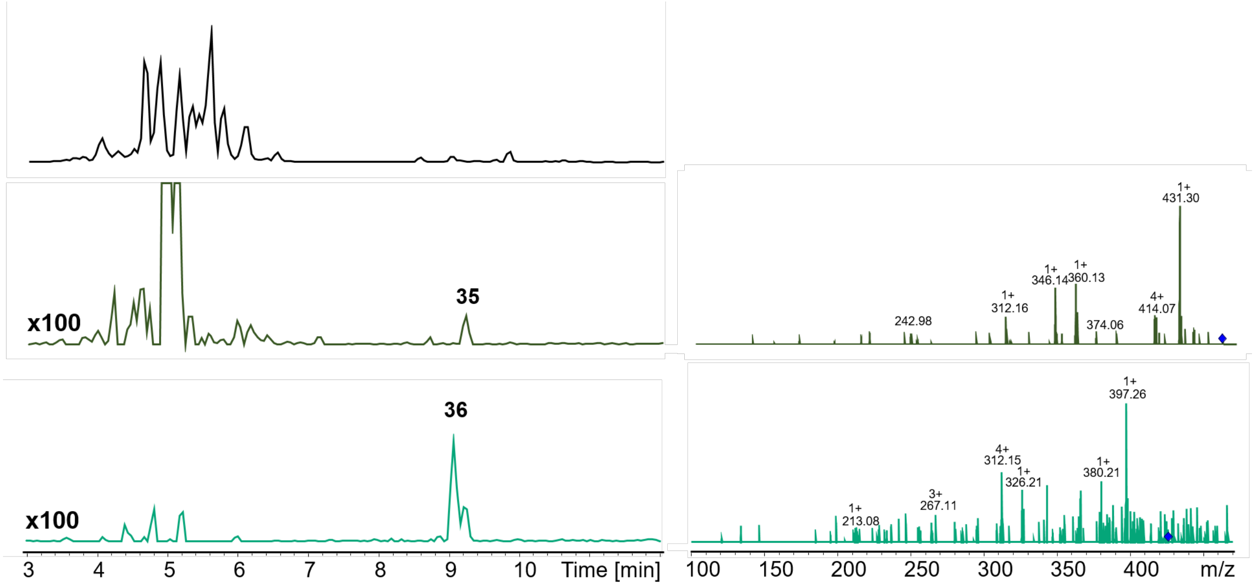

Figure S39. HPLC/MS data refers to Figure 4 (NRPS-29) of compounds 35 and 36 produced in *E. coli* DH10B::*mtaA*. (a) BPC of an exemplary culture extract. (b) EIC/MS^2 data of **35** (m/z $[M+H]^+$ = 459.30). (c) EIC/MS^2 data of **36** (m/z $[M+H]^+$ = 425.31).

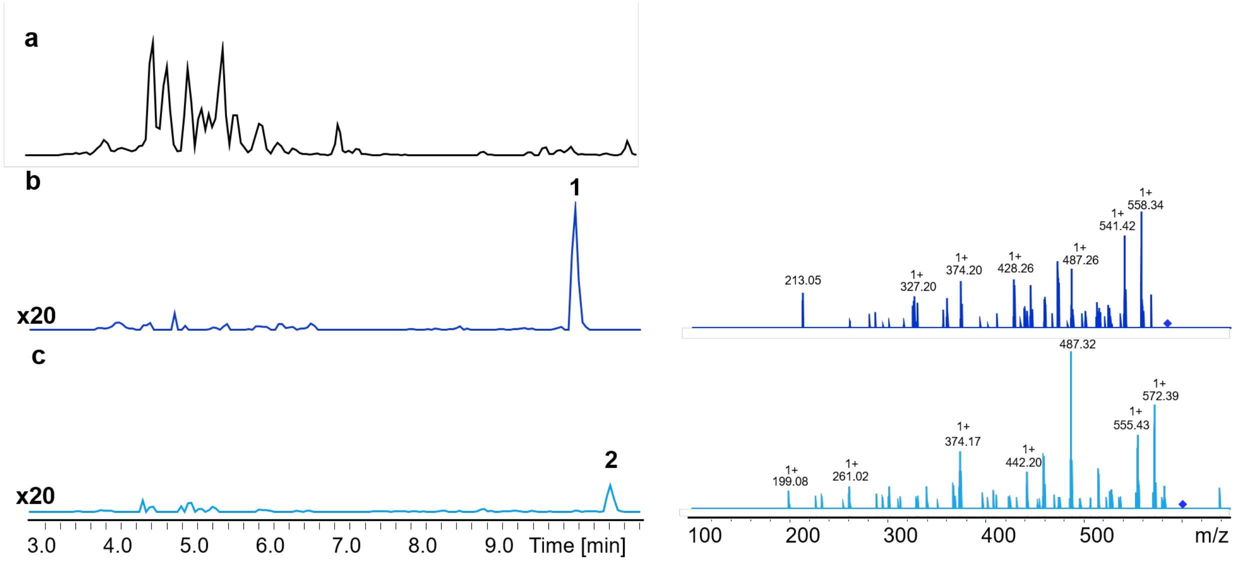

Figure S40. HPLC/MS data refers to Figure 4 (NRPS-30) of compounds 1 and 2 produced in *E. coli* DH10B::*mtaA*. (a) BPC of an exemplary culture extract. (b) EIC/MS^2 data of **1** (m/z $[M+H]^+$ = 586.40). (c) EIC/MS^2 data of **2** (m/z $[M+H]^+$ = 600.41).

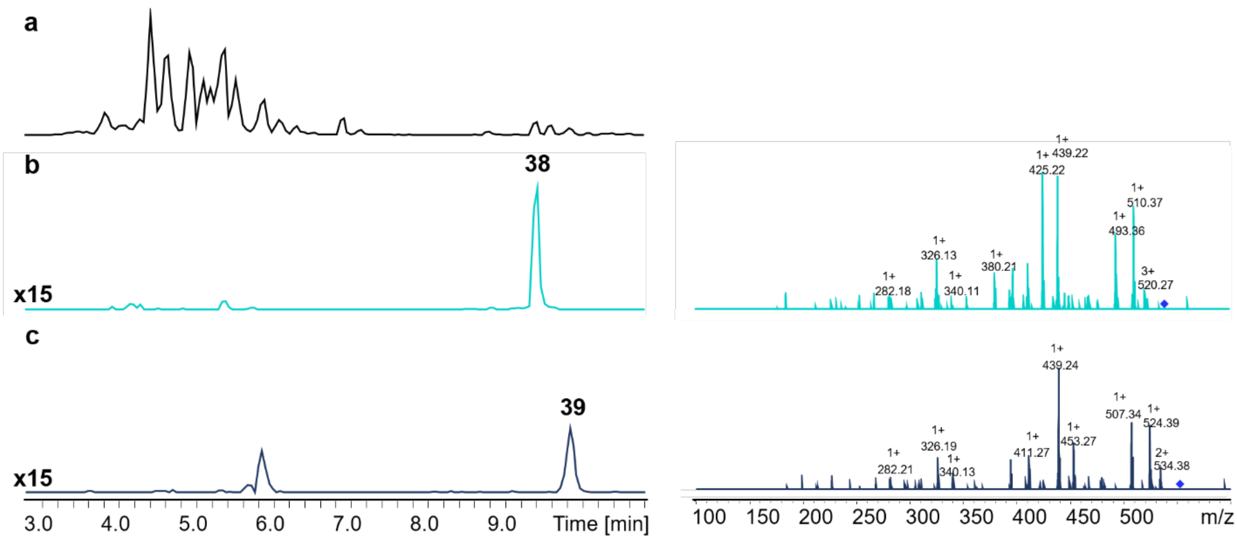

Figure S41. HPLC/MS data refers to Figure 4 (NRPS-31) of compounds 38 and 39 produced in *E. coli* DH10B::*mtaA*. (a) BPC of an exemplary culture extract. (b) EIC/MS[2] data of **38** (*m/z* $[M+H]^+$ = 588.40). (c) EIC/MS[2] data of **39** (*m/z* $[M+H]^+$ = 552.41).

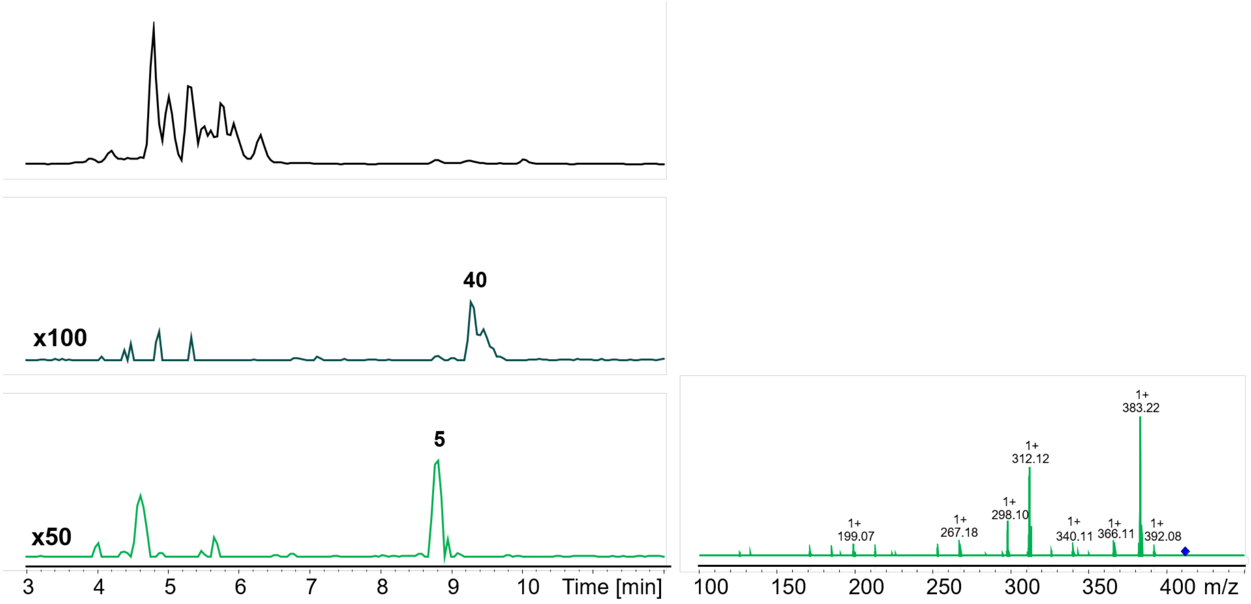

Figure S42. HPLC/MS data refers to Figure 4 (NRPS-32) of compounds 40 and 5 produced in *E. coli* DH10B::*mtaA*. (a) BPC of an exemplary culture extract. (b) EIC data of **40** (*m/z* $[M+H]^+$ = 425.31). (c) EIC/MS[2] data of **5** (m/z $[M+H]^+$ = 411.29).

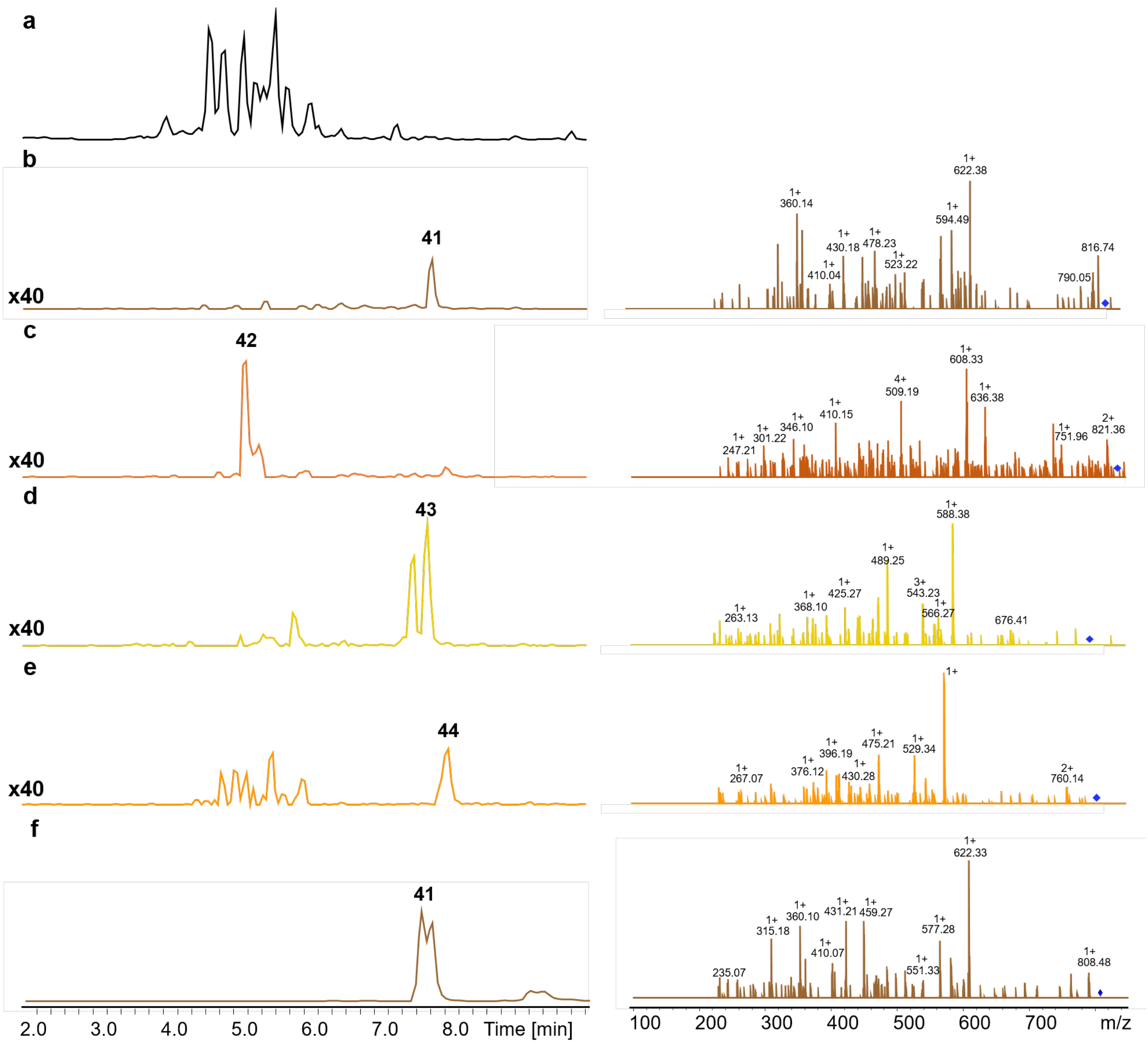

Figure S43. HPLC/MS data refers to Figure 4 (NRPS-33) of compounds 41, 42, 43 and 44 produced in *E. coli* DH10B::*mtaA*. (a) BPC of an exemplary culture extract. (b) EIC/MS^2 data of **41** (*m/z* $[M+H]^+$ = 826.45). (c) EIC/MS^2 data of **42** (*m/z* $[M+H]^+$ = 840.47). (d) EIC/MS^2 data of **43** (*m/z* $[M+H]^+$ = 792.47). (e) EIC/MS^2 data of **44** (*m/z* $[M+H]^+$ = 806.48). (f) EIC/MS^2 data of synthetic **41** (*m/z* $[M+H]^+$ = 826.45).

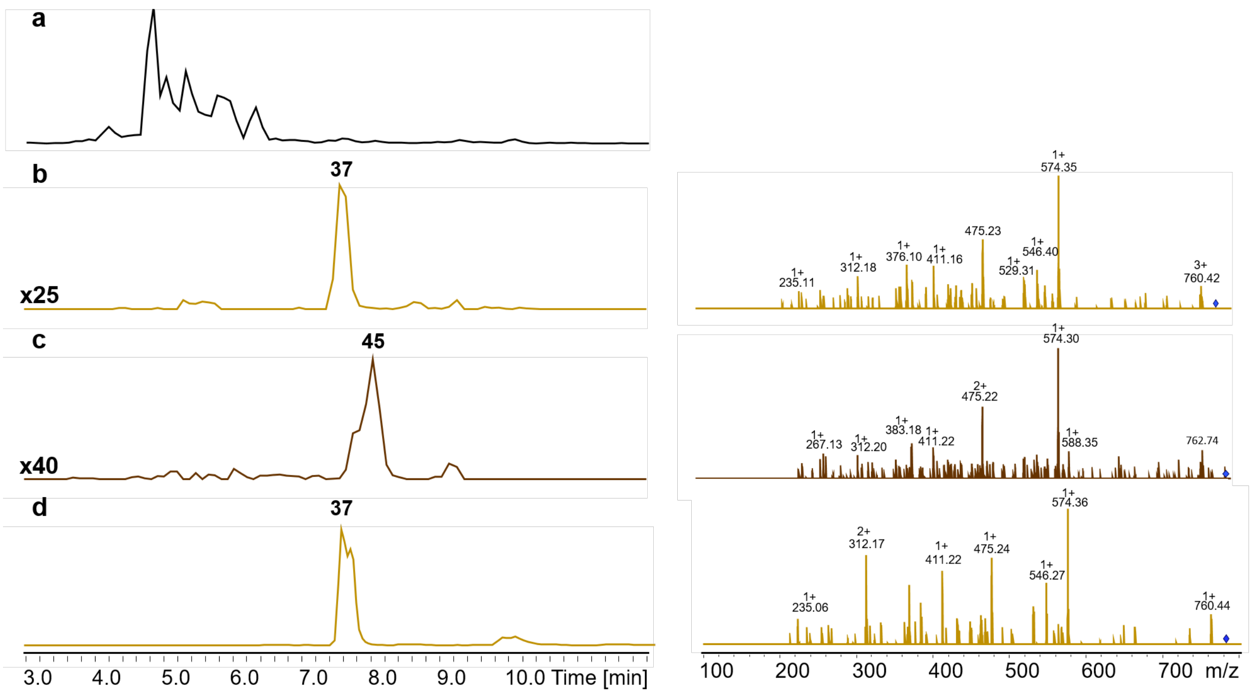

Figure S44. HPLC/MS data refers to Figure 4 (NRPS-34) of compounds 37 and 45 produced in *E. coli* DH10B::*mtaA*. (a) BPC of an exemplary culture extract. (b) EIC/MS^2 data of **37** (*m/z* $[M+H]^+$ = 778.45). (c) EIC/MS^2 data of **45** (*m/z* $[M+H]^+$ = 792.47). (d) EIC/MS^2 data of synthetic **37** (*m/z* $[M+H]^+$ = 778.45).

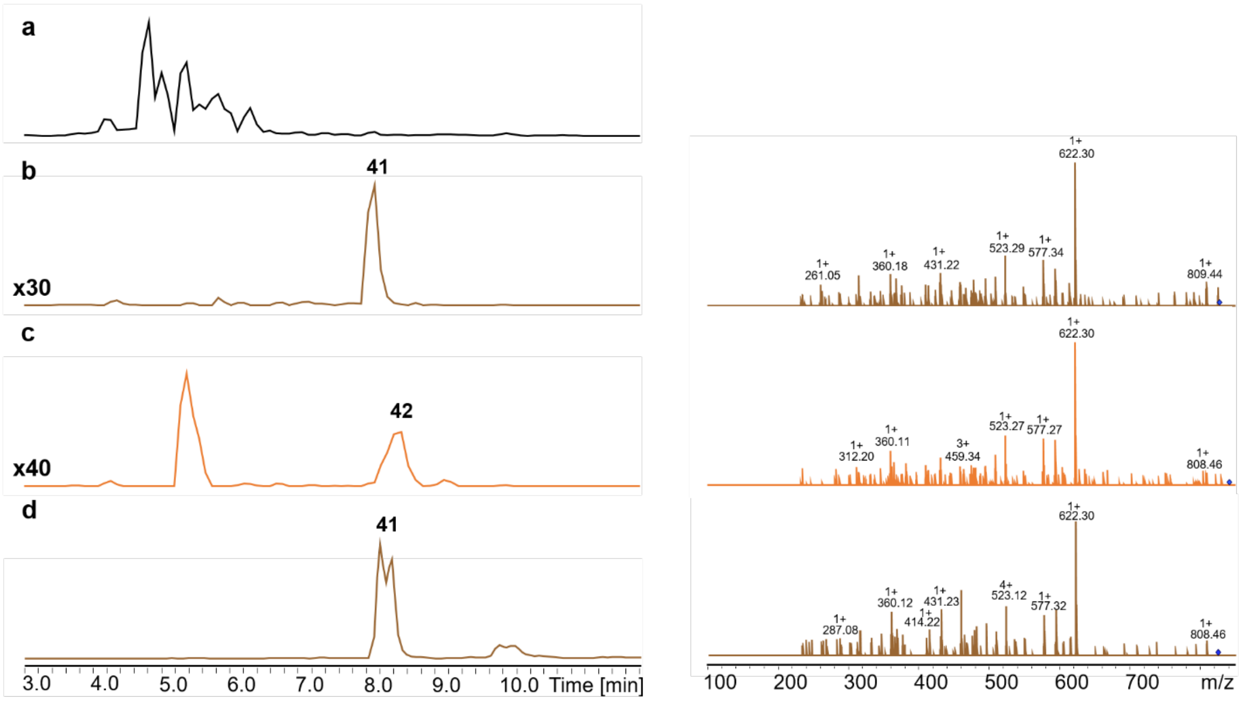

Figure S45. HPLC/MS data refers to Figure 4 (NRPS-35) of compounds 41 and 42 produced in *E. coli* DH10B::*mtaA*. (a) BPC of an exemplary culture extract. (b) EIC/MS^2 data of **41** (*m/z* $[M+H]^+$ = 826.45). (c) EIC/MS^2 data of **42** (*m/z* $[M+H]^+$ = 840.47). (d) EIC/MS^2 data of synthetic **41** (*m/z* $[M+H]^+$ = 826.45).

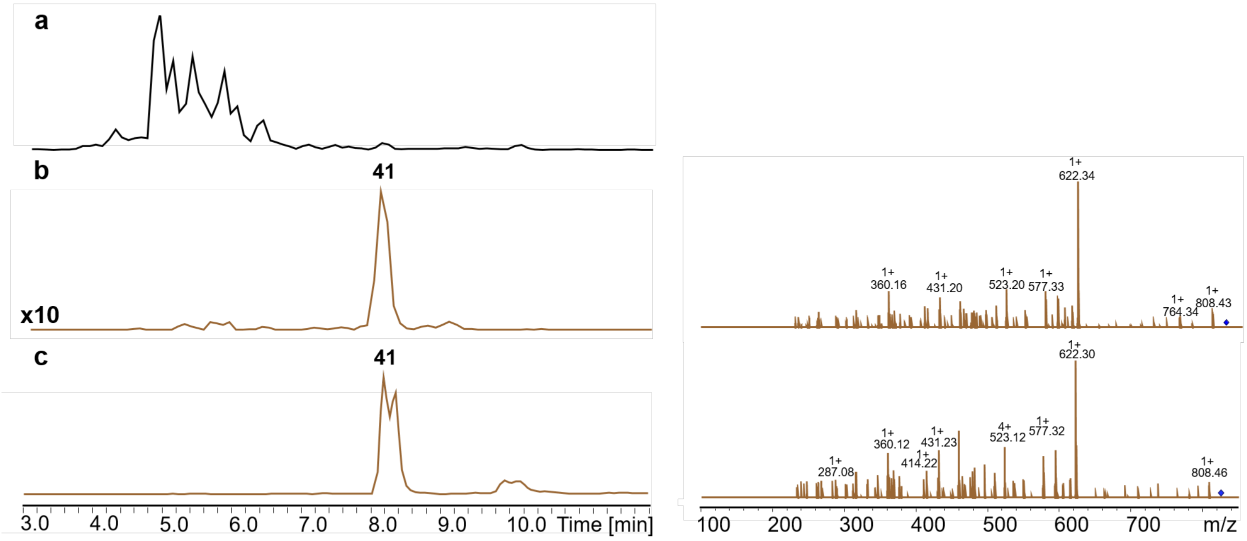

Figure S46. HPLC/MS data refers to Figure 4 (NRPS-36) of compound 41 produced in *E. coli* DH10B::*mtaA*. (a) BPC of an exemplary culture extract. (b) EIC/MS^2 data of **41** (*m/z* $[M+H]^+$ = 826.45). (c) EIC/MS^2 data of synthetic **41** (*m/z* $[M+H]^+$ = 826.45).

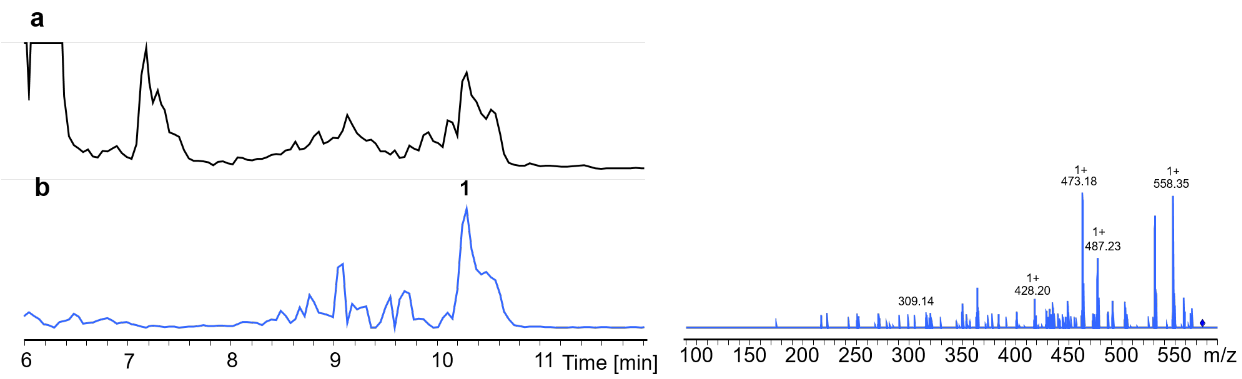

Figure S47. HPLC/MS data refers to Figure 4 (NRPS-37) of compound 1 produced in *E. coli* DH10B::*mtaA*. (a) BPC of an exemplary culture extract. (b) EIC/MS^2 data of **1** (*m/z* $[M+H]^+$ = 586.40).

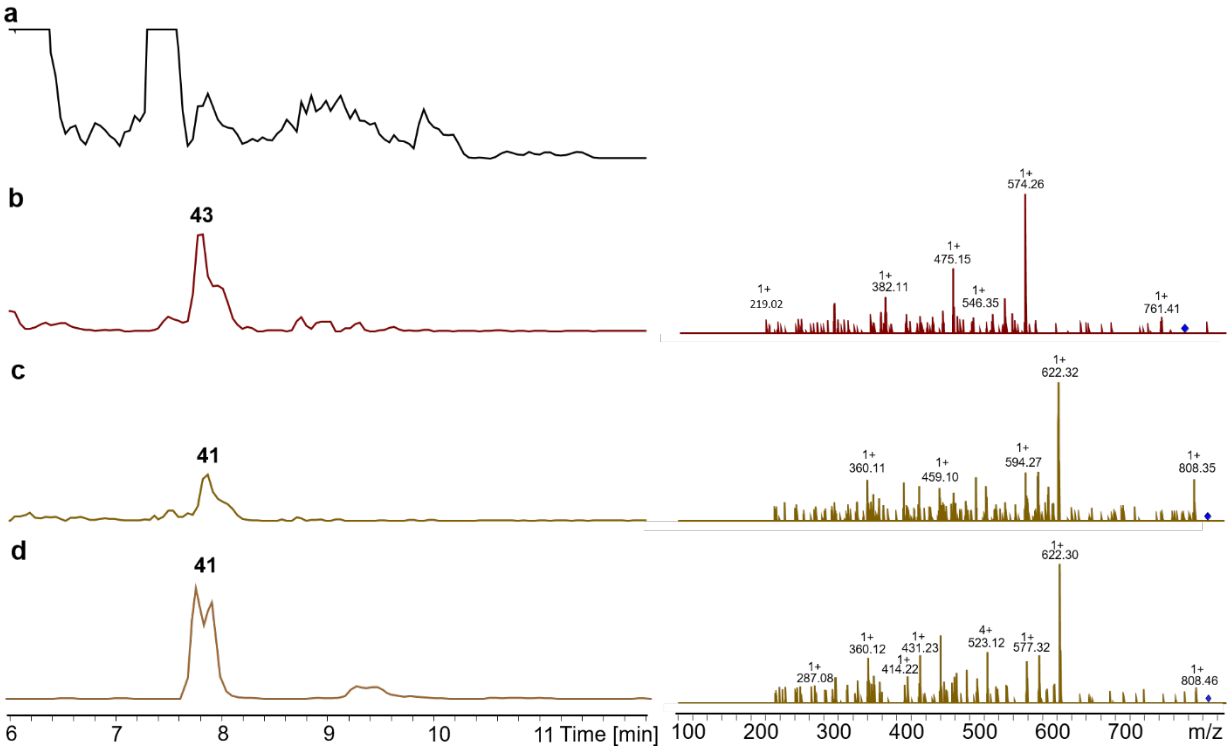

Figure S48. HPLC/MS data refers to Figure 4 (NRPS-38) of compounds 43 and 41 produced in *E. coli* DH10B::*mtaA*. (a) BPC of an exemplary culture extract. (b) EIC/MS^2 data of **43** (*m/z* $[M+H]^+$ = 792.47). (c) EIC/MS^2 data of **41** (*m/z* $[M+H]^+$ = 826.45). (d) EIC/MS^2 data of synthetic **41** (*m/z* $[M+H]^+$ = 826.45).

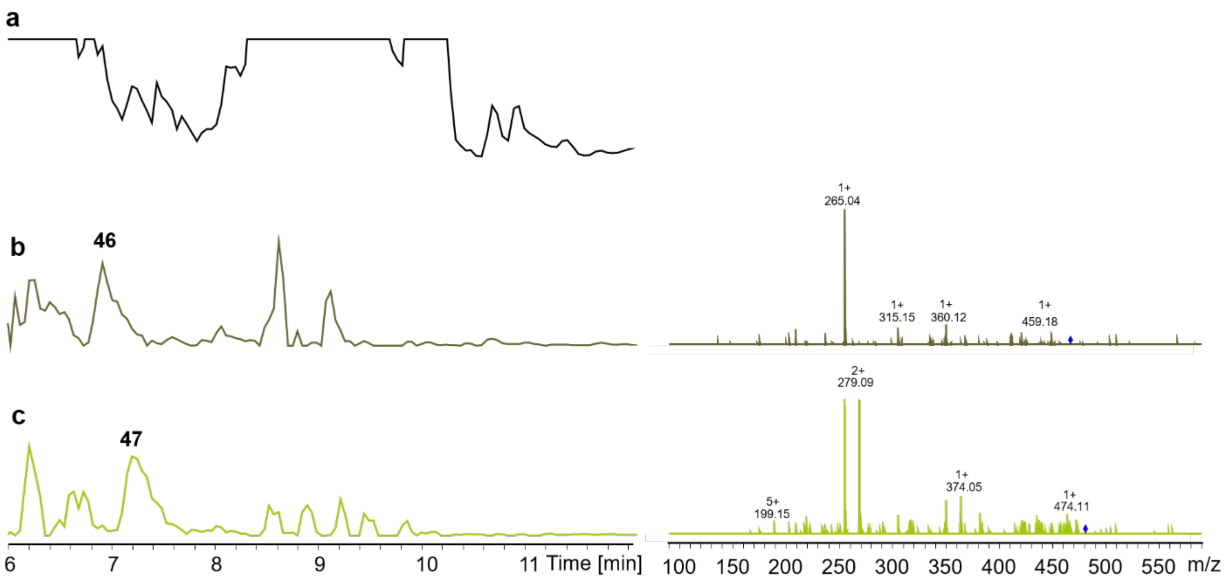

Figure S49. HPLC/MS data refers to Figure 4 (NRPS-39) of compounds 46 and 47 produced in *E. coli* DH10B::*mtaA*. (a) BPC of an exemplary culture extract. (b) EIC/MS^2 data of **46** (*m/z* $[M+H]^+$ = 476.30). (c) EIC/MS^2 data of **47** (*m/z* $[M+H]^+$ = 490.32).

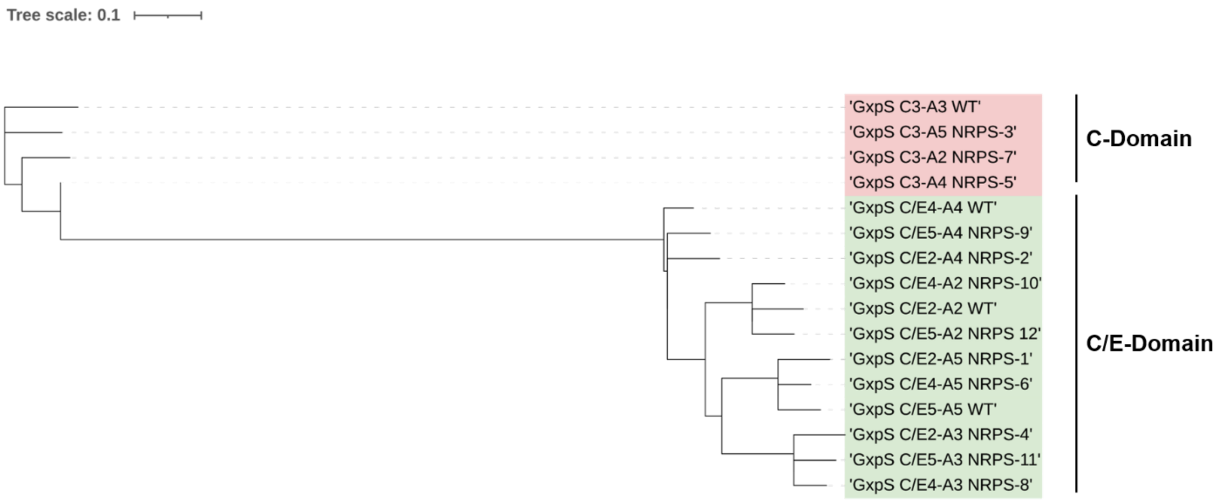

Figure S50. Phylogenetic tree of extracted C-A and C/E-A didomains from GxpS. WT C-A and C/E-A didomains were aligned with non-natural C-A and C/E-A didomains from NRPS-1 to -16. Alignment was used to build a tree applying RAxML. Results show a clear clustering of the C-domains into C- and C/E indicating the importance of C-domain types. A-domain specificity clustering only becomes clear on the second level. C-Domain type did not change for NRPS-9, -2, -10, -12, -1 and -6. C-domain changes from C to C/E applies for NRPS-4, -11 and -8 and changes from C/E to C applies for NRPS-3, -7 and -5.

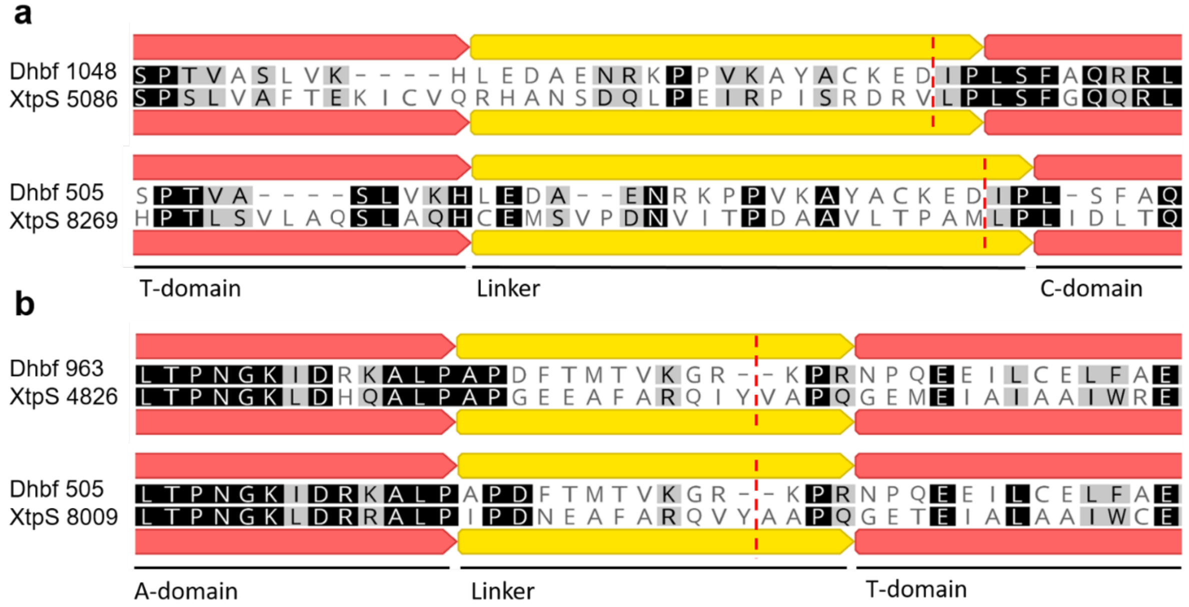

Figure S51. Alignments of XtpS linker sequences. (a) XtpS T_2-C_2 (XtpS 5086) and T_3-C_3 (XtpS 8269) were aligned to the linker region excised from Dhbf crystal structure (Protein Database ID: 5U89). (b) XtpS A_2-T_2 (XtpS 4826) and A_3-T_3 (XtpS 8009) were aligned to the linker region excised from Dhbf crystal structure. Red dashed lines indicate SYNZIP insertion points.

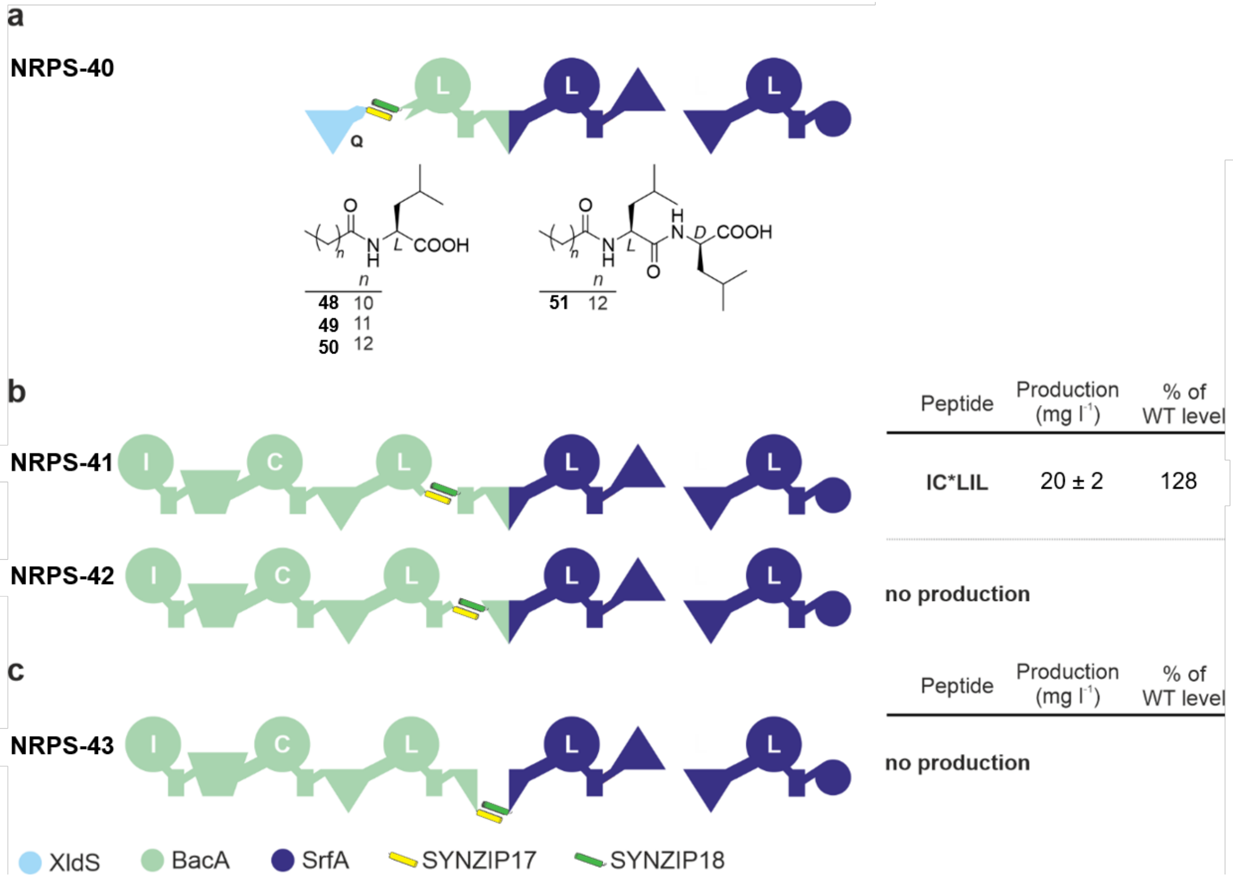

Figure S52. Additional examples of bipartite type S NRPS split in between and within RtpS modules[1].

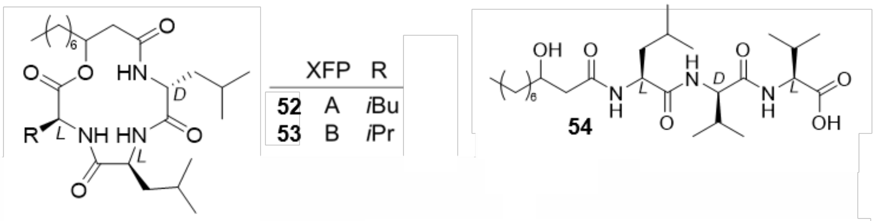

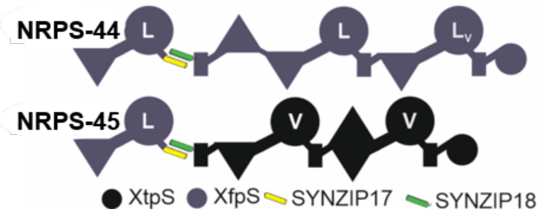

Peptide	Production (mg l^{-1})	% of WT level
cyclo[10:0(β-hydroxy)-LLL] **52**	0.30 ± 0.03	27 ± 3
cyclo[10:0(β-hydroxy)-LLV] **53**	0.30 ± 0.08	73 ± 17
10:0(β-hydroxy)-LVV **54**	0.06 ± 0.02	14 ± 5

Figure S53. Additional examples of bipartite type S NRPS split within modules of xefoampeptide-producing NRPS (XfpS)[8] and between XfpS and XtpS.

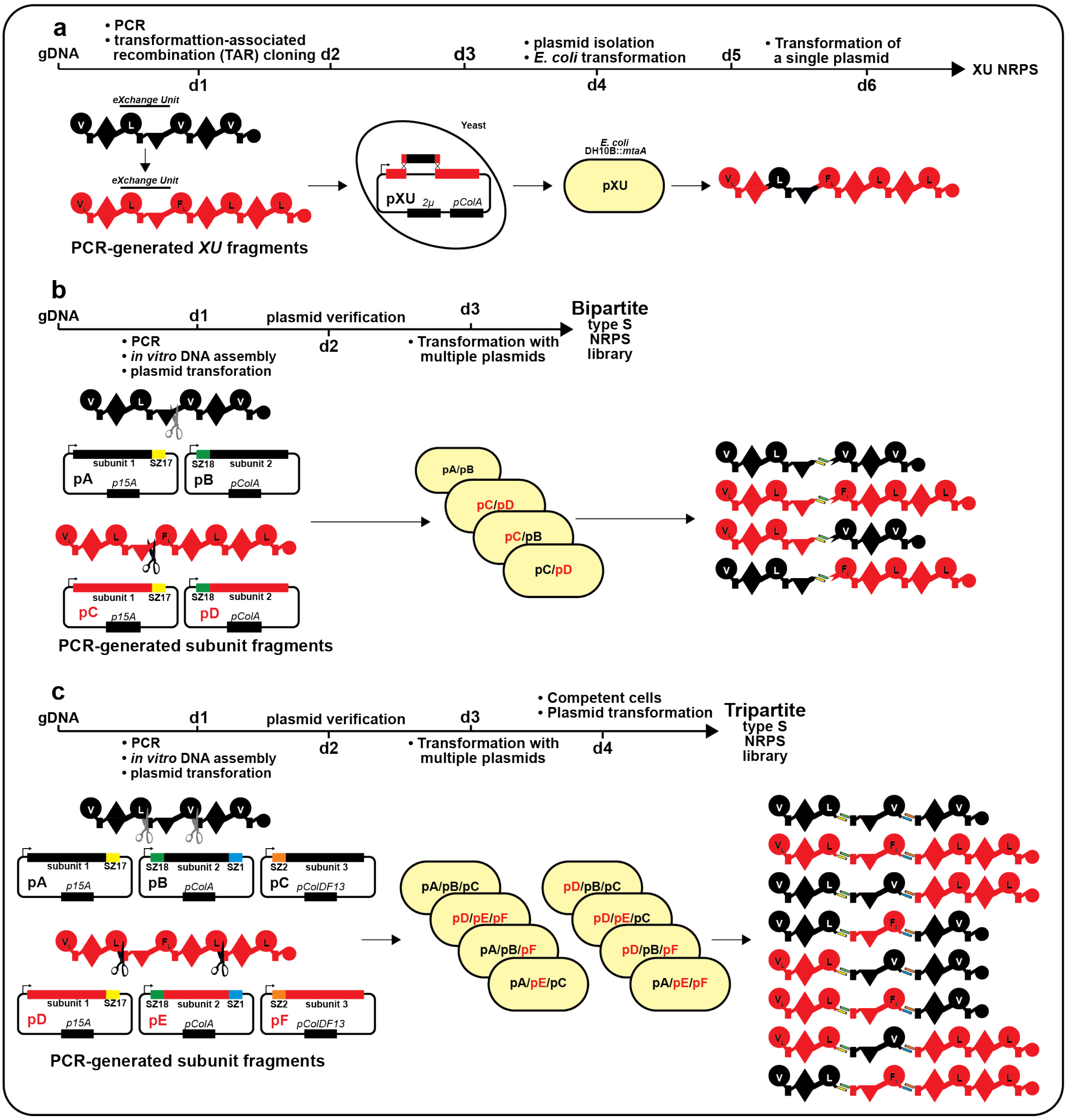

Figure S54. Extended comparison of workflows to generate chimeric NRPSs. (a) “Classic” Yeast-based TAR cloning approach. Here, two BGCs were used to generate one chimeric NRPS applying the XU concept. Ideally, cloning of one chimeric NRPS takes 6 days. (b) Generation of bipartite typeS NRPS libraries. Here, two BGCs were split within the XU concept fusion site and generated subunits 1 and 2 were assembled into two distinct plasmid BBs carrying differing ORIs and resistance cassettes. Subunit 1 plasmids and subunit 2 plasmids were co-transformed into *E. coli* DH10B::*mtaA* in different combinations resulting in four typeS NRPSs. Cloning can be achieved in only half the days (3 days) compared to TAR cloning. Furthermore, throughput of recombinant NRPS can be increased exponentially with the number of building blocks. (c) Generation of tripartite typeS NRPS libraries. Here, two BGCs were split twice within the A-T linker region. Subunits 1-3 were assembled into three distinct plasmids carrying differing ORIs and resistance cassettes. Subunit 1, subunit 2 und subunit 3 plasmids were co-transformed into *E. coli* DH10B::*mtaA* in different combinations. Cloning ca be archived within 4 days. Throughput increases more rapidly compared to bipartite typeS NRPS.

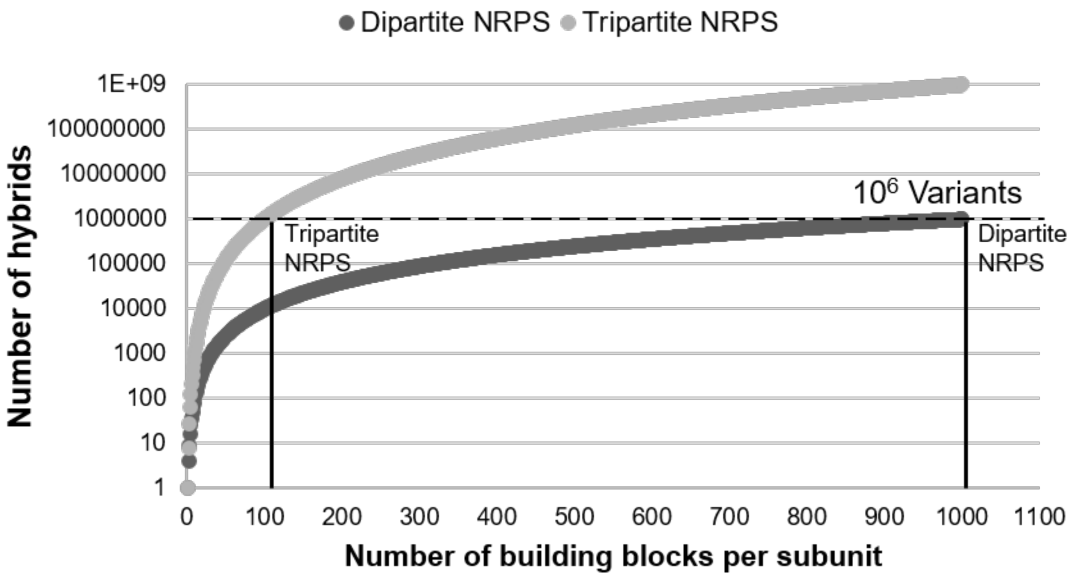

Figure S55. Comparing the throughput between di- and tripartite NRPSs. Number of hybrids increases exponentially with the number of building blocks per subunit for both di- and tripartite NRPSs. The number of hybrids increases more rapidly for dipartite NRPS. To build a library with 10^6 variants, 1000 building blocks per subunit (in total 2000) for dipartite NRPS and only 100 building blocks per subunit (in total 300) for tripartite NRPS are required.

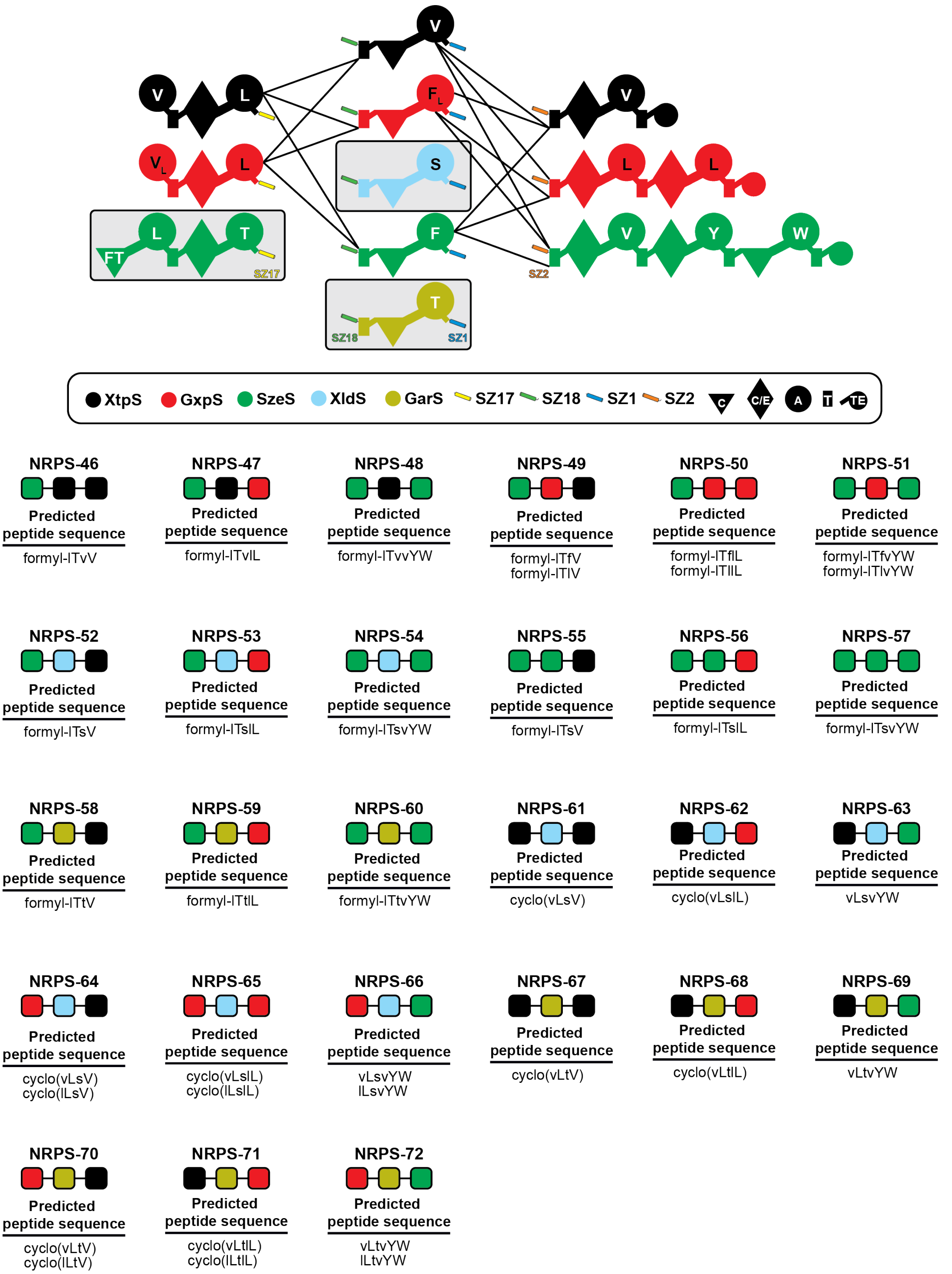

Figure S56. Remaining possible tripartite typeS NRPSs and their predicted peptide sequences. Grey boxes highlight non-functional building blocks.

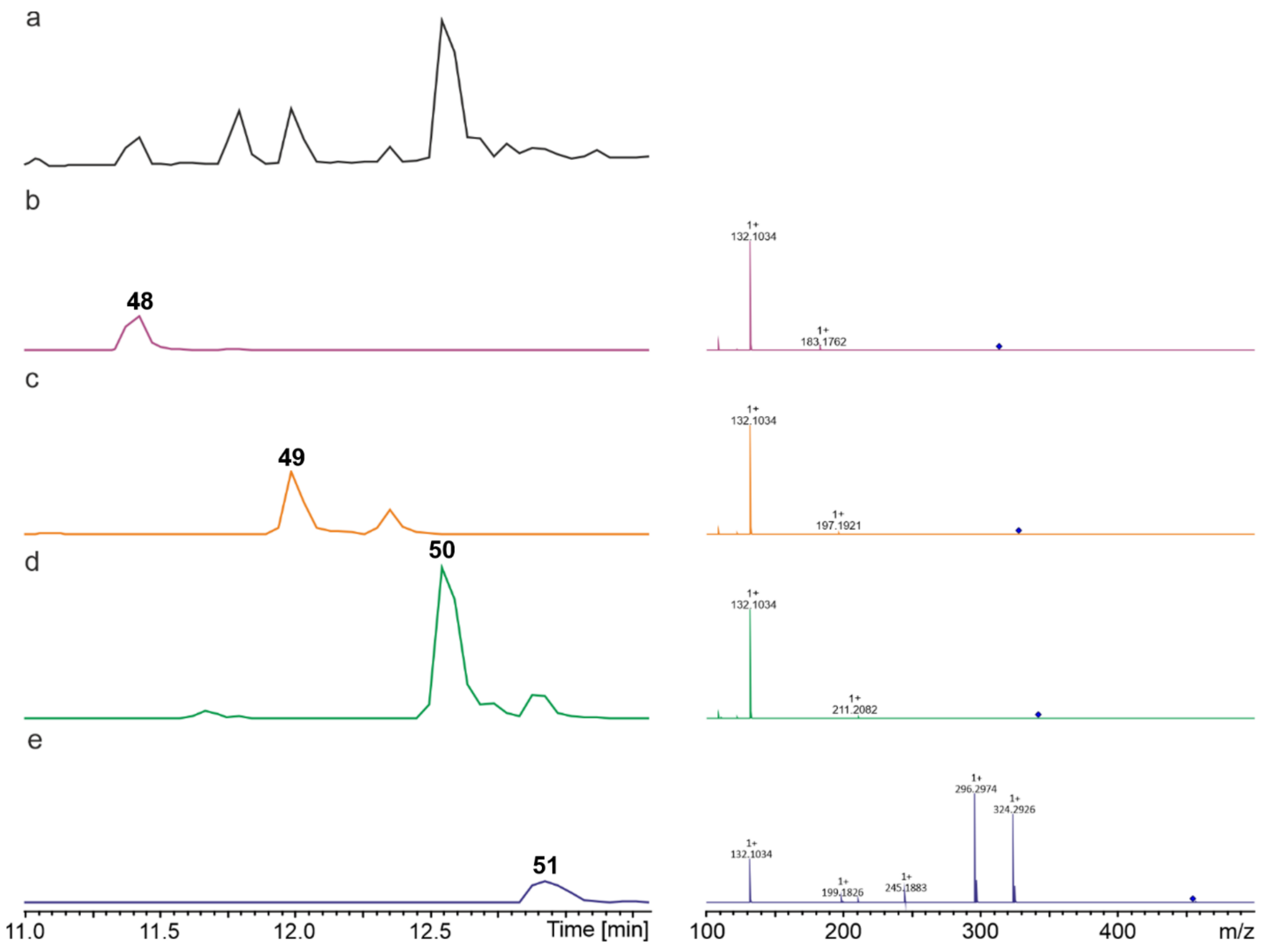

Figure S57. HPLC/MS data refers to Supplementary Figure 40 (NRPS-40) of compounds 48, 49, 50 and 51 produced in *E. coli* DH10B::*mtaA*. (a) BPC of an exemplary culture extract. (b) EIC/MS2 data of 48 (*m/z* $[M+H]^+$ = 314.27). (c) EIC/MS2 data of 49 (*m/z* $[M+H]^+$ = 328.29). (d) EIC/MS2 data of 50 (*m/z* $[M+H]^+$ = 342.20). (e) EIC/MS2 data of 51 (*m/z* $[M+H]^+$ = 455.38).

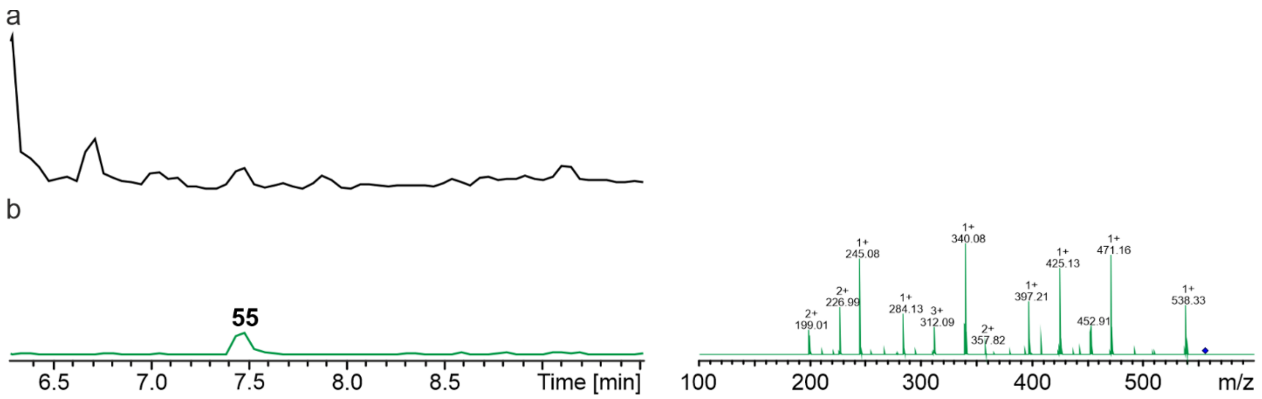

Figure S58. HPLC/MS data refers to Supplementary Figure 41 (NRPS-41) of compound 55 produced in *E. coli* DH10B::*mtaA*. (a) BPC of an exemplary culture extract. (b) EIC/MS^2 data of **55** (*m/z* $[M+H]^+$ = 556.35).

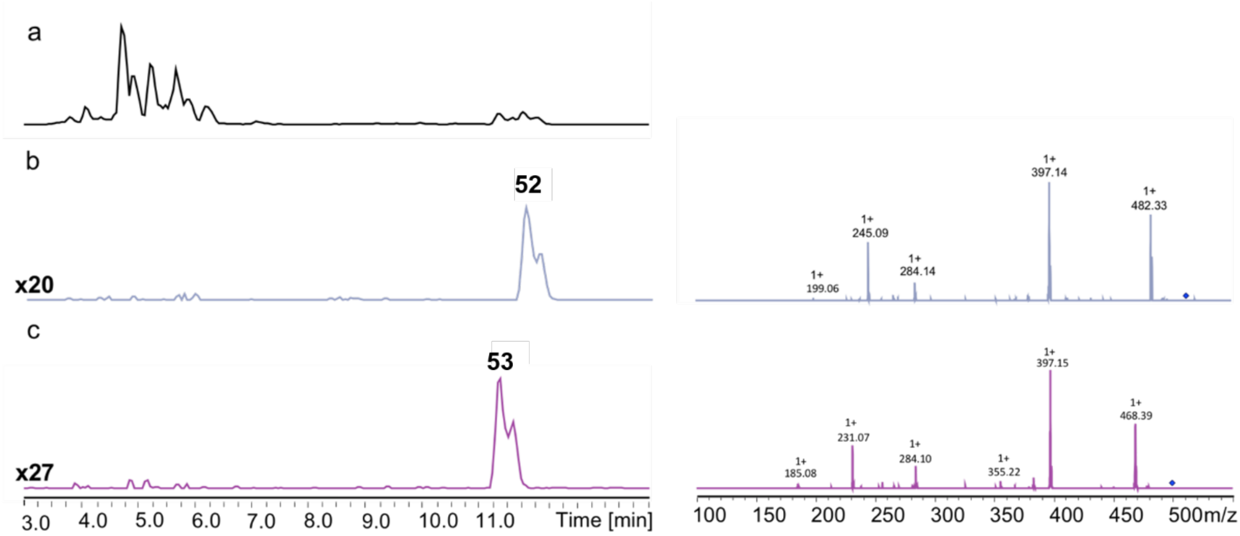

Figure S59. HPLC/MS data refers to Supplementary Figure 41 (NRPS-44) of compounds 52 and 53 produced in *E. coli* DH10B::*mtaA*. (a) BPC of an exemplary culture extract. (b) EIC/MS2 data of 52 (*m/z* $[M+H]^+$ = 510.39). (c) EIC/MS2 data of 53 (*m/z* $[M+H]^+$ = 496.37).

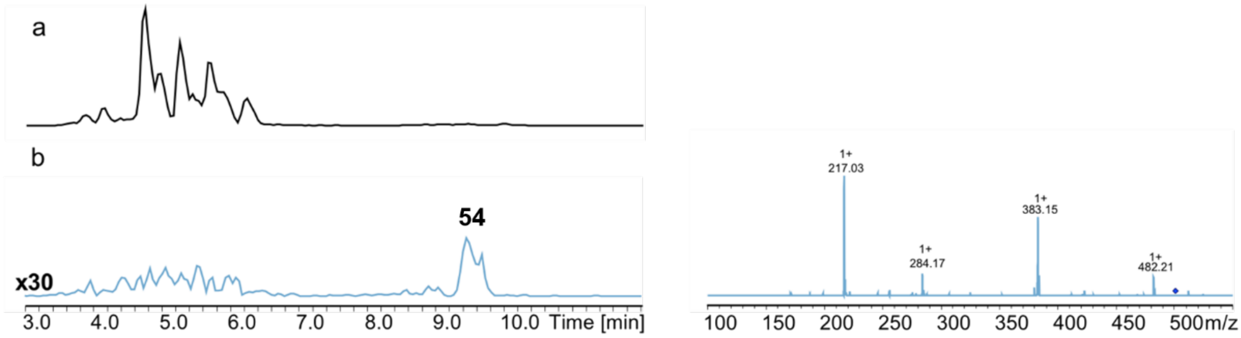

Figure S60. HPLC/MS data refers to Supplementary Figure 41 (NRPS-45) of compound 54 produced in *E. coli* DH10B::*mtaA*. (a) BPC of an exemplary culture extract. (b) EIC/MS2 data of 43 (*m/z* $[M+H]^+$ = 500.37).

4. Reference

[1] K. A. J. Bozhüyük, J. Watzel, N. Abbood, H. B. Bode, *Angew Chem Int Ed Engl* **2021**, *60*, 17531-17538.

[2] K. A. J. Bozhüyük, F. Fleischhacker, A. Linck, F. Wesche, A. Tietze, C. P. Niesert, H. B. Bode, *Nat Chem* **2018**, *10*, 275-281.

[3] F. I. Nollmann, C. Dauth, G. Mulley, C. Kegler, M. Kaiser, N. R. Waterfield, H. B. Bode, *Chembiochem* **2015**, *16*, 205-208.

[4] C. Kegler, F. I. Nollmann, T. Ahrendt, F. Fleischhacker, E. Bode, H. B. Bode, *Chembiochem* **2014**, *15*, 826-828.

[5] D. Hanahan, *J Mol Biol* **1983**, *166*, 557-580.

[6] O. Schimming, F. Fleischhacker, F. I. Nollmann, H. B. Bode, *Chembiochem* **2014**, *15*, 1290-1294.

[7] J. M. Chaston, G. Suen, S. L. Tucker, A. W. Andersen, A. Bhasin, E. Bode, H. B. Bode, A. O. Brachmann, C. E. Cowles, K. N. Cowles, C. Darby, L. de Léon, K. Drace, Z. Du, A. Givaudan, E. E. Herbert Tran, K. A. Jewell, J. J. Knack, K. C. Krasomil-Osterfeld, R. Kukor, A. Lanois, P. Latreille, N. K. Leimgruber, C. M. Lipke, R. Liu, X. Lu, E. C. Martens, P. R. Marri, C. Médigue, M. L. Menard, N. M. Miller, N. Morales-Soto, S. Norton, J. C. Ogier, S. S. Orchard, D. Park, Y. Park, B. A. Qurollo, D. R. Sugar, G. R. Richards, Z. Rouy, B. Slominski, K. Slominski, H. Snyder, B. C. Tjaden, R. van der Hoeven, R. D. Welch, C. Wheeler, B. Xiang, B. Barbazuk, S. Gaudriault, B. Goodner, S. C. Slater, S. Forst, B. S. Goldman, H. Goodrich-Blair, *PLoS One* **2011**, *6*, e27909.

[8] C. Kegler, H. B. Bode, *Angew Chem Int Ed Engl* **2020**, *59*, 13463-13467.
